# The risk associated with spinal manipulation: an overview of reviews

**DOI:** 10.1186/s13643-017-0458-y

**Published:** 2017-03-24

**Authors:** Sabrina Mai Nielsen, Simon Tarp, Robin Christensen, Henning Bliddal, Louise Klokker, Marius Henriksen

**Affiliations:** 1 The Parker Institute, Copenhagen University Hospital, Frederiksberg & Bispebjerg, Frederiksberg, 2000 Denmark; 2 Clinical Research Unit, The Parker Institute, Copenhagen University Hospital, Frederiksberg & Bispebjerg, Frederiksberg, 2000 Denmark; 3 Physiotherapy and Biomechanics Research Unit, The Parker Institute, Copenhagen University Hospital, Frederiksberg & Bispebjerg, Frederiksberg, 2000 Denmark

## Abstract

**Background:**

Spinal manipulative therapy (SMT) is a widely used manual treatment, but many reviews exist with conflicting conclusions about the safety of SMT. We performed an overview of reviews to elucidate and quantify the risk of serious adverse events (SAEs) associated with SMT.

**Methods:**

We searched five electronic databases from inception to December 8, 2015. We included reviews on any type of studies, patients, and SMT technique. Our primary outcome was SAEs. Quality of the included reviews was assessed using a measurement tool to assess systematic reviews (AMSTAR). Since there were insufficient data for calculating incidence rates of SAEs, we used an alternative approach; the conclusions regarding safety of SMT were extracted for each review, and the communicated opinion were judged by two reviewers independently as safe, harmful, or neutral/unclear. Risk ratios (RRs) of a review communicating that SMT is safe and meeting the requirements for each AMSTAR item, were calculated.

**Results:**

We identified 283 eligible reviews, but only 118 provided data for synthesis. The most frequently described adverse events (AEs) were stroke, headache, and vertebral artery dissection. Fifty-four reviews (46%) expressed that SMT is safe, 15 (13%) expressed that SMT is harmful, and 49 reviews (42%) were neutral or unclear. Thirteen reviews reported incidence estimates for SAEs, roughly ranging from 1 in 20,000 to 1 in 250,000,000 manipulations. Low methodological quality was present, with a median of 4 of 11 AMSTAR items met (interquartile range, 3 to 6). Reviews meeting the requirements for each of the AMSTAR items (i.e. good internal validity) had a higher chance of expressing that SMT is safe.

**Conclusions:**

It is currently not possible to provide an overall conclusion about the safety of SMT; however, the types of SAEs reported can indeed be significant, sustaining that some risk is present. High quality research and consistent reporting of AEs and SAEs are needed.

**Systematic review registration:**

PROSPERO CRD42015030068.

**Electronic supplementary material:**

The online version of this article (doi:10.1186/s13643-017-0458-y) contains supplementary material, which is available to authorized users.

## Background

Spinal manipulative therapy (SMT) is a manual treatment where a vertebral joint is passively moved between the normal range of motion and the limits of its anatomic range, though a universally accepted definition does not seem to exist [1]. SMT often involves a high-velocity, low-amplitude thrust, a technique in which the joints are adjusted rapidly, often accompanied by popping sounds [2, 3].

The use of SMT dates back to 400 BCE, but during the centuries, SMT has switched between being accepted and abandoned by the medical profession [4]. Today, SMT is included in many guidelines for primary care, such as the management of non-specific low back pain [5], and several evidence-based guidelines exist on the practice of SMT [6–10]. SMT is widely used; it has been estimated that 12% of adults in the USA and Canada are attending chiropractors each year, with 80% of the visits involving SMT [11, 12], and use of SMT has been increasing in the past several decades [13]. Various professional groups are performing SMT including chiropractors, osteopaths and manual therapists [14]. SMT is used for a wide range of diseases and conditions with frequent indications being neck and back pain [13]. Patient satisfaction is high [13], but the evidence on the effectiveness of SMT from randomized controlled trials (RCTs) is often unconvincing [14–17].

As with all interventions, there are risks associated with SMT. Possible harmful outcomes of SMT includes, but are not limited to, headache, radiating discomfort and fatigue [18], which are often transient, but also more serious events such as death, stroke, paralysis and fractures [19–22]. What the patients define as mild, moderate and major AEs depend on the severity of the pain or symptom, the impact on their function, the duration and by ruling out other causes for the AEs [23]. Currently, the knowledge about the risk of harms associated with SMT is fragmented since an enormous amount of literature exists on the topic, but with different conclusions. For instance, two retrospective population-based studies have suggested an association between vertebrobasilar strokes and chiropractic care (which usually involves spinal manipulation), but also a similar association with primary care physician visits [24, 25]. Another study concluded that SMT is independently associated with vertebral artery dissection [26]. Thus, uncertainty arises when single studies are reviewed, and there is a need for an overview of the field. To our knowledge, no one has provided a complete overview of what is known about the safety of SMT. Therefore, we performed an overview of reviews to elucidate and quantify the risk of serious adverse events (SAEs) associated with SMT regardless of the indications for the treatment.

## Methods

A brief protocol was registered in the International Prospective Register of Systematic Reviews (PROSPERO: CRD42015030068) prior to the initiation of this overview [see protocol in Additional file 1]. This review was reported according to PRISMA harms [27] [see the completed checklist in Additional file 2].

### Literature search

We searched Cochrane Database of Systematic Reviews, Cochrane Database of Abstracts of Reviews of Effects (DARE), Cochrane Health Technology Assessment Database (HTA), MEDLINE via PubMed (from 1966) and EMBASE via Ovid (from 1974). The original search was conducted on December 8, 2015 and updated on January 10, 2017, and no date restrictions were used. Our main search terms consisted of the terms spinal adjustment, chiropractic, and spine -, spinal -, lumbar -, back -, neck -, cervical -, thrust -, or osteopath manipulation, in addition to the MeSH term ‘Manipulation, Chiropractic’. Our systematic review filter included the terms Cochrane, CENTRAL, MEDLINE, EMBASE, pubmed, search, systematic review, meta-analysis, comparative effectiveness, indirect - and mixed treatment comparison, and systematic literature [see Additional file 3, showing the search strategy used]. References from relevant reviews, overviews of reviews and relevant national clinical guidelines were checked to identify additional relevant reviews.

### Study selection

We included official health technology assessment reports and peer-reviewed reviews of studies of any type (including cohorts, case reports, etc.) that examine individuals receiving SMT. We did not require the SMT to be within a certain definition but relied on the definitions used by the review authors. No restrictions were put on the age, nationality, gender or health status of the population, or length of follow-up of the study. The control could be sham, placebo, any or none. At least an abstract in English, Danish, Swedish or Norwegian had to be available. For inclusion in the synthesis, data on AEs was required.

In order to ensure that the included reviews were conducted in a systematic manner, a criterion for inclusion was to include the following two items from a measurement tool to assess systematic reviews (AMSTAR): ‘were two or more electronic sources searched?’ and ‘was the scientific quality of the included studies assessed and documented?’ [28, 29], as done by other overview authors [30, 31]. Since no commonly accepted quality assessment tool exists for case reports, case series, cross-sectional studies or surveys, quality assessments of these study types were not required.

One reviewer (SMN) screened titles and abstracts, and subsequently reviewed full texts to identify relevant reviews for the overview. A second reviewer (MH) was consulted when the basis for decision making was not clear. We contacted authors of studies that could not be retrieved in full text.

### Data extraction

The same reviewer (SMN) performed the data extraction, and the same second reviewer (MH) was consulted, when the basis for decision making was not clear. When possible, we extracted only data for patients receiving SMT, when other interventions were included in a review.

The primary outcome was SAEs defined as conditions requiring hospital admission (or mortality) [32], and the secondary outcome was any AEs reported. AEs were defined as ‘any untoward occurrence that may present during treatment’ [32]. If the severity of an AE was not defined in the review, one reviewer (MH) rated the severity of the reported AEs, and when the basis for rating was unclear, another reviewer (HB) was consulted. No attempt was made to contact authors of reviews or primary studies to obtain missing data.

It was pre-specified in our protocol that the AEs and SAEs should be summarized for each review with a subsequent synthesis and meta-analysis. However, the available data on AEs and SAEs were too heterogeneously and insufficiently reported. Instead, we appraised the communicated opinions of each review concerning the safety of SMT based on their conclusions regarding the AEs and SAEs. This was done by two reviewers independently (SMN, LK), who judged the communicated opinions as either ‘safe’, ‘neutral/unclear’ or ‘harmful’, based on the qualitative impression the reviewers had when reading the conclusions. The reviewers had no opinion about the safety/harmfulness of SMT before commencing the judgements. Cohen’s weighted Kappa was calculated for the agreement between the reviewers, with a value of 0.40–0.59 indicating ‘fair agreement’, 0.60–0.74 indicating ‘good agreement’ and ≥0.75 indicating ‘excellent agreement’ [33]. Disagreements were resolved by a third reviewer (MH).

### Quality assessment

One reviewer (SMN) assessed the methodological quality of each review using the AMSTAR tool [28, 29]. AMSTAR consists of 11 criteria, where each was given one of the ratings: ‘yes’ (clearly done), ‘can’t answer’ (unclear if completed), ‘no’ (clearly not done) or ‘not applicable’. A second reviewer (MH) was consulted when the basis for decision making was not clear. We calculated a summary score by awarding each ‘yes’ with one point for each review [28]. A score of 0–4 is often classified as low quality, 5–8 as moderate quality and 9–11 as high [34].

We did not assess the quality of the evidence presented by each of the reviews. However, if a quality of evidence assessment (such as a GRADE assessment) was reported in the reviews, the approach and result were extracted.

### Data analysis

To get an ‘objective’ measure of our confidence in the subjectively judged communicated opinions, we assessed whether a pattern of communicated opinions could be identified according to methodological quality of the reviews (i.e. AMSTAR). This was done by calculating a risk ratio (RR) of a review communicating the opinion ‘safe’ when meeting the requirements for each AMSTAR item, and a RR of the opinion of a review communicating ‘harmful’ when meeting the requirements for each AMSTAR item. The decision to conduct this assessment and subsequent analyses were, however, done post hoc.

Risk estimates for SAEs reported in the reviews are presented in a separate table, and a matrix was constructed showing which studies the estimates from each review were based on. All statistical analyses were performed using the statistical software R, version 3.2.3 (R Foundation for Statistical Computing).

## Results

### Study selection

**Fig. 1:**
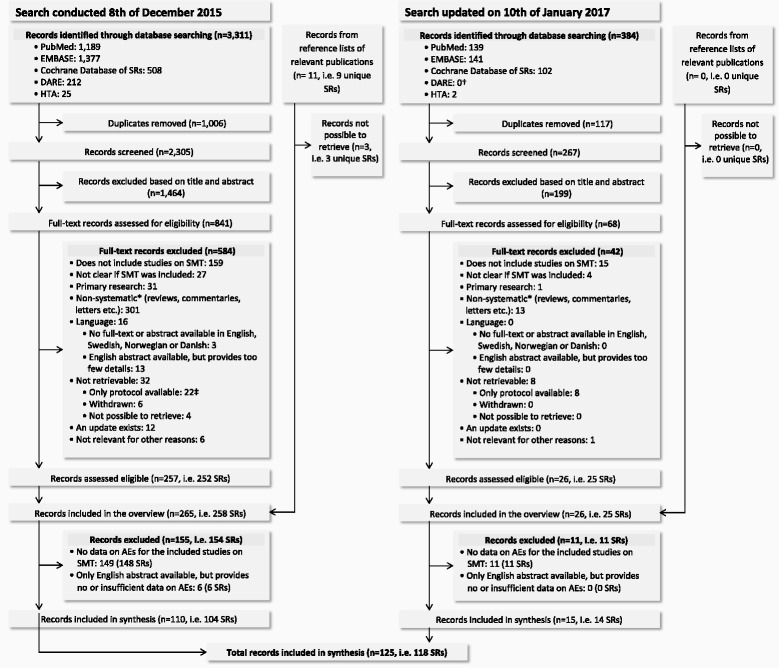
Flow diagram. AEs = adverse events; DARE = Cochrane Database of Abstracts of Reviews of Effects; HTA = Cochrane Health Technology Assessment Database; RCT = randomized controlled trial; SMT = spinal manipulative therapy; SRs = systematic reviews. *Non-systematic: does not report to have searched at least two electronic databases or does not document an assessment of the quality of the included studies (case reports, case series, cross-sectional studies and surveys were not required to have been quality assessed). † The DARE database stopped updating March 2015. ‡ Four of these protocols resulted in a systematic review which was retrieved in the updated search

The reviewer screened 2305 records and identified 841 potentially eligible records (Fig. 1). Thirteen authors were contacted regarding studies that could not be retrieved in full-text. Twelve authors responded of which 9 were able to provide full-text versions. Reviewing full-texts resulted in 257 records describing 252 reviews eligible for the overview [see Additional file 4 for a list of the excluded reviews]. From reference lists, we further identified 8 records on 6 eligible reviews. In total, 265 records describing 258 reviews were included in the overview [see Additional file 5 for a list of the 258 included studies]; of these, 110 records describing 104 reviews were included in the synthesis. The updated search resulted in screening of 267 additional records, identifying 68 potentially eligible records. Of these, 26 records describing 25 reviews were eligible for the overview, and 15 records describing 14 reviews were included in the synthesis. In total, 283 reviews were included in the overview, of which 118 reviews were included in the synthesis.

### Characteristics of the included reviews

The main characteristics of the 118 reviews included are presented in Table 1 [see Table, Additional file 6, which shows further study characteristics]. The included reviews consisted of 13 Cochrane reviews [14–17, 35–46], 41 other reviews including only RCTs [47–87], 53 reviews including study types other than RCTs [88–140], 3 guidelines [9, 141–143] and 8 health technology assessments [144–154].

**Table 1:** Summary of findings for spinal manipulative therapy

Year	Authors	Main objectives	Number of pts receiving intervention	Any AEs reported	Any SAEs reported	Estimate for the incidence of SAEs	Communicated opinion
2016	Blanchette, M. A. et al. [80]	Effect	460	Yes	No	No	Safe
2016	Cerritelli, F. et al. [81]	Effect	31	No	No	No	Neutral/unclear
2016	Chou, R. et al. [154]	Both	>1100	Yes	No	No	Neutral/unclear
2016	Church, E. W. et al. [137]	AEs	5934	Unclear	Yes	No	Safe
2016	Hall, H. et al. [83]	Effect	249	No	No	No	Neutral/unclear
2016	Page Matthew, J. et al. [46]	Both	117	No	No	No	Safe
2016	Ruddock, J. K. et al. [84]	Effect	261	Yes	No	No	Neutral/unclear
2016	Ruffini, N. et al. [139]	Effect	52	No	No	No	Neutral/unclear
2016	Varatharajan, S. et al. [85]	Effect	129	Yes	No	No	Safe
2016	Wearing, J. et al. [140]	Effect	55	Yes	No	No	Safe
2016	Wong, J. J. et al. [86]	Effect	369	Yes	No	No	Safe
2016	Yao, M. et al. [87]	Effect	1135	Yes	Yes	No	Safe
2015	Cicchintti L. et al. [116]	Effect	NA	Yes	No	No	Safe
2015	Franke, H. et al. [82]	Both	67	Yes	No	No	Safe
2015	Gross A. J. et al. [36]	Effect	NA	Yes	No	No	Safe
2015	Liddle S. D. & Pennick V. [35]	Effect	289	Unclear	No	No	Safe
2015	Posadzki, P. et al. [138]	Effect	NA	Unclear	Unclear	No	Neutral/unclear
2015	Puentedura E. J. & O'Grady W. H. [134]	AEs	10	Yes	Yes	No	Harmful
2015	Southerst D. et al. [49]	Effect	98	Yes	No	No	Safe
2015	Yuan Q.-L. et al. [48]	Effect	208	No	No	No	Safe
2015	Zhu L. et al. [47]	Effect	NA	No	No	No	Neutral/unclear
2014	Bryans R. et al. [9]	Both	513	Yes	No	No	Safe
2014	Clar C. et al. [101]	Effect	NA	Yes	Yes	No	Neutral/unclear
2014	Close C. et al. [53]	Effect	NA	Yes	No	No	Neutral/unclear
2014	Franke H. et al. [52]	Effect	779	Yes	No	No	Safe
2014	Kizhakkeveettil A. et al. [51]	Effect	1799	Unclear	Unclear	No	Neutral/unclear
2014	Page M. J. et al. [37]	Effect	4	No	No	No	Safe
2014	Sutton D. et al. [50]	Effect	813	Yes	No	No	Safe
2014	Todd A. J. et al. [98]	AEs	>34,605	Yes	Yes	Yes	Harmful
2014	Tuchin P. [130]	AEs	9	Yes	No	No	Neutral/unclear
2014	Yin P. et al. [93]	AEs	94	Yes	Yes	No	Harmful
2014	Young J. L. et al. [105]	Effect	539	Yes	No	No	Safe
2013	Brantingham J. W. et al. [97]	Effect	109	Yes	Unclear	No	Safe
2013	Hebert J. J. et al. [103]	AEs	77	Yes	Yes	No	Neutral/unclear
2013	Huisman P. A. et al. [57]	Effect	350	Yes	No	No	Neutral/unclear
2013	Parkinson L. et al. [109]	Effect	>520	No	No	No	Safe
2013	Posadzki P. et al. [56]	Effect	>448	Yes	No	No	Neutral/unclear
2013	Scholten-Peeters G. G. M. et al. [55]	Effect	626	Yes	No	No	Safe
2013	Schroeder J. et al. [54]	Effect	195	Yes	Unclear	No	Safe
2013	Wynd S. et al. [126]	AEs	901	Unclear	Yes	No	Neutral/unclear
2013	Yang M. et al. [38]	Effect	39	Yes	No	No	Safe
2012	Brantingham J. W. et al. [102]	Effect	>109	Yes	No	No	Safe
2012	Dobson D. et al. [40]	Effect	116	No	No	No	Neutral/unclear
2012	Furlan A. D. et al. [148, 149]	Both	NA	Yes	Yes	No	Neutral/unclear
2012	Gleberzon B. J. et al. [106]	Effect	NA	Yes	Unclear	No	Safe
2012	Haynes M. J. et al. [125]	AEs	NA	Unclear	Yes	No	Neutral/unclear
2012	Kuczynski J. J. et al. [60]	Effect	268	Yes	No	No	Safe
2012	Lin J. H. et al. [59]	Effect	283	No	No	No	Neutral/unclear
2012	Posadzki P. & Ernst E. [58]	Effect	NA	Yes	No	No	Neutral/unclear
2012	Puentedura E. J. et al. [123]	AEs	134	Yes	Yes	No	Harmful
2012	Rubinstein S. M. et al. [15, 39]	Effect	1195	Yes	No	No	Safe
2012	Stuber K. A. et al. [132]	AEs	NA	Yes	Yes	No	Neutral/unclear
2011	Brantingham J. et al. [104]	Effect	>266	No	No	No	Neutral/unclear
2011	Cross K. et al. [65]	Effect	187	Yes	No	No	Safe
2011	Huang T. et al. [43]	Effect	131	Yes	No	No	Safe
2011	Lystad R. P. et al. [112]	Effect	NA	Yes	No	No	Safe
2011	Posadzki P. & Ernst E. [63]	Effect	NA	Yes	No	No	Harmful
2011	Posadzki P. & Ernst E. [62]	Effect	NA	Yes	No	No	Harmful
2011	Posadzki P. & Ernst E. [61]	Effect	NA	Unclear	Unclear	No	Neutral/unclear
2011	Posadzki P. & Ernst E. [64]	Effect	NA	Yes	No	No	Neutral/unclear
2011	Rubinstein S. M. et al. [14, 42]	Effect	2435	Yes	No	No	Safe
2011	Walker B. F. et al. [41, 44]	Effect	NA	Yes	No	No	Neutral/unclear
2010	Carlesso L. C. et al. [96]	AEs	NA	Yes	No	No	Neutral/unclear
2010	Carnes D. et al. [88]	AEs	25,179	Yes	Yes	Yes	Safe
2010	Ernst E. [128]	AEs	26	Unclear	Yes	No	Harmful
2010	Hahne A. J. et al. [66]	Effect	NA	No	No	No	Safe
2010	Kaminskyj A. et al. [108]	Effect	NA	Yes	No	No	Neutral/unclear
2010	Shin B.-C. et al. [95]	AEs	18	Yes	Yes	No	Harmful
2009	Boudreau R. et al. [147]	Effect	>52	Yes	Yes	No	Safe
2009	Boudreau R. & Spry C. [151]	Effect	1	No	No	No	Safe
2009	Brurberg K. G. et al. [145]	Effect	>695	Yes	No	No	Safe
2009	Gouveia L. O. et al. [99]	AEs	>2838	Yes	Yes	Yes	Harmful
2009	Hunt K. J. et al. [67]	Effect	NA	Yes	No	No	Safe
2009	Khorsan B. et al. [94]	Effect	>297	Unclear	Yes	No	Neutral/unclear
2009	Reiman M. P. et al. [110]	Effect	>76	Yes	Unclear	No	Neutral/unclear
2008	Miley M. L. et al. [127]	AEs	NA	Unclear	Yes	Yes	Harmful
2008	Stuber K. J. & Smith D. L. [107]	Effect	285	No	No	No	Neutral/unclear
2008	Vernon H. & Humphreys B. K. [68]	Effect	178	Yes	No	No	Safe
2007	Chou R. & Huffman L. H. [141, 143]	Effect	NA	Yes	Yes	Yes	Safe
2007	Ernst E. [118]	AEs	>924	Yes	Yes	No	Harmful
2007	Gross A. R. et al. [71]	Effect	NA	Unclear	No	No	Safe
2007	Hawk C. et al. [100]	Effect	NA	Yes	No	No	Safe
2007	Luijsterburg P. A. J. et al. [70]	Effect	175	No	No	No	Neutral/unclear
2007	Vernon H. & Humphreys B. K. [69]	Effect	701	Yes	No	No	Neutral/unclear
2007	Vernon H. et al. [113]	Effect	593	Yes	No	No	Safe
2006	Gemmell H. & Miller P. [111]	Effect	>79	Unclear	Unclear	No	Safe
2006	Proctor M. et al. [16]	Effect	>162	Yes	Unclear	No	Safe
2006	Snelling N. J. [135]	Both	>214	Yes	Yes	Yes	Neutral/unclear
2005	Brown A. et al. [146]	Effect	NA	Yes	Yes	No	Safe
2005	Ernst E. [133]	AEs	14	Yes	Yes	No	Harmful
2005	Hondras M. A. et al. [17]	Effect	NA	No	No	No	Safe
2005	Lisi A. J. et al. [120]	Effect	183	Yes	No	No	Neutral/unclear
2005	Rubinstein S. M. et al. [91]	AEs	7	Unclear	Yes	No	Neutral/unclear
2004	Brønfort G. et al. [45]	Effect	85	Yes	No	No	Safe
2004	Ernst E. [89]	AEs	340	Yes	Yes	No	Neutral/unclear
2004	Lenssinck M.-L. B. et al. [72]	Effect	NA	Yes	No	No	Safe
2004	Oduneye F. [152]	Effect	128	Yes	No	No	Neutral/unclear
2004	Oliphant D. [115]	AEs	NA	Yes	Yes	Yes	Safe
2003	Ernst E. [90]	AEs	2	Yes	Yes	No	Harmful
2002	Ernst E. [119]	AEs	>4	Yes	Yes	No	Neutral/unclear
2002	Ernst E. [136]	AEs	42	Yes	Yes	No	Harmful
2002	Gerritsen A. A. M. et al. [74]	Effect	45	Yes	No	No	Safe
2002	Gross A. R. et al. [142]	Both	NA	Yes	Yes	Yes	Neutral/unclear
2002	Gross A. R. et al. [73]	Effect	NA	Yes	No	No	Neutral/unclear
2002	Stevinson C. & Ernst E. [124]	AEs	>2357	Yes	Yes	Yes	Neutral/unclear
2001	Bronfort G. et al. [76]	Effect	400	Yes	No	No	Safe
2001	Ernst E. [129]	AEs	>2016	Yes	No	No	Neutral/unclear
2001	Ernst E. & Harkness E. [75]	Effect	NA	Yes	No	No	Neutral/unclear
2000	Ernst E. [77]	Effect	NA	Yes	No	No	Neutral/unclear
2000	Magee D. J. et al. [117]	Effect	10	No	No	No	Safe
1999	Fabio R. P. D. [121]	Both	177	Yes	Yes	No	Harmful
1999	Haldeman S. M. et al. [122]	AEs	115	Unclear	Yes	No	Safe
1999	Vernon H. et al. [78]	Effect	176	Yes	No	No	Neutral/unclear
1996	Aker P. D. et al. [79]	Effect	NA	Yes	No	No	Neutral/unclear
1996	Assendelft W. J. J. et al. [131]	AEs	>1795	Yes	Yes	Yes	Neutral/unclear
1996	Hurwitz E. L. et al. [144, 150]	Effect	>935	Yes	Yes	Yes	Neutral/unclear
1995	Dabbs V. & Lauretti W. J. [114]	AEs	NA	Unclear	Yes	Yes	Safe
1992	Shekelle P. G. et al. [92]	Effect	>1500	Unclear	Yes	Yes	Neutral/unclear

When ‘Number of patients in total’ has ‘>’ in front, the actual number of patients is higher since incomplete data were provided by the review

*AEs* adverse events, *NA* no data available, *pts* patients, *SAEs* serious adverse events

The vast majority of the reviews investigated SMT (either as the only intervention or as a separate subgroup). Some of these reviews further specified SMT as cervical, thoracic or lumbar SMT (21 reviews [46, 47, 49, 54, 57, 65, 91, 96, 103, 105, 114, 115, 119, 121, 123, 125–127, 134, 136, 150]). Other reviews did not further specify than ‘manipulation’ (10 reviews [36, 66, 70–73, 79, 93, 101, 107]), ‘osteopathic manipulative treatment/therapy’ (8 reviews [38, 52, 56, 64, 81, 82, 116, 139]), and ‘chiropractic care/interventions’ (5 reviews [67, 98–100, 137]).

The populations most frequently studied were patients with cervical pain, low back pain or headache (based on a word count after categorization by the authors; Table 2). For 81 of the reviews, the main aim was to investigate efficacy (benefit), for 29 of the reviews, the main aim was to investigate AEs, and for the remaining 8, the aim was to investigate both.

**Table 2:** The patient populations most frequently studied in the included reviews (listed after frequency shown in brackets)

1. Cervical pain (25)	
2. Low back pain (18)	
3. Headache (16)	
4. Children/adolescents (6)	
5. Asthma (4)	
6. Cervical radiculopathy (4)	
7. Musculoskeletal (various) (4)	
8. Pregnant (4)	
9. Dysmenorrhea (3)	
10. Lumbar radiculopathy (3)	
11. Pelvic pain (3)	
12. Carpal tunnel syndrome (2)	
13.Phobia (2)	
14. Cervical trauma (1)	
15. Chronic inflammatory disease (1)	
16. Chronic obstructive pulmonary disease (1)	
17. Colic (1)	
18. Diversity of complaints (1)	
19. Dizziness (1)	
20. Frozen shoulder (1)	

A word count of the reported AEs and SAEs showed that the most frequently used term describing AEs/SAEs in the reviews was stroke (counted after categorization by the authors; Table 3). However, it should be noted that a very common subject in the discussion sections was the poor reporting of AEs in the primary studies and the possible risk of underreporting. Thirteen of the reviews reported estimates for the incidence of SAEs, and also here, many of the reviews noted that these were rough estimates [see Table, Additional file 6, which includes conclusions extracted from each reviews].

**Table 3:** The terms describing the adverse events and serious adverse events most frequently used in the reviews (listed after frequency shown in brackets)

1. Stroke (36)	
2. Headache (34)	
3. Vertebral artery dissection (29)	
4. Increased pain (22)	
5. Fatigue (18)	
6. Aggravation of symptoms (17)	
7. Death (17)	
8. Radiculopathy (17)	
9. Soreness (16)	
10. Spinal cord injury (16)	
11. Cauda equine syndrome (15)	
12. Disc herniation (13)	
13. Vertebral fracture (12)	
14. Discomfort (11)	
15. Minor side effects (10)	
16. Stiffness (10)	
17. Dizziness (9)	
18. Nausea (9)	
19. Vertebral dislocation (8)	
20. Neck-stiffness (7)	

### The methodological quality of included reviews

None of the reviews met the requirements for all 11 AMSTAR items (Table 4). The median number of ‘yes’ was 4 (interquartile range, 3 to 6), with a minimum and maximum of 0 and 9 ‘yes’ respectively. Only very few reviews had combined (e.g. in meta-analysis or other means of synthesis) the findings of AEs and SAEs or done this in an appropriate way; hence, item 9 was not applicable in most cases. One of the reviews made an attempt to assess the publication bias specifically for AEs and/or SAEs; hence, this one review met the requirements for item 10.

**Table 4:** Methodological quality of included reviews assessed with AMSTAR

Year	Authors	1	2	3	4	5	6	7	8	9	10	11	Total score^a^
2016	Blanchette, M. A. et al. [80]	Yes	Yes	Yes	Yes	No	Yes	Yes	Yes	NA	No	No	7
2016	Cerritelli, F. et al. [81]	No	Yes	Yes	Yes	No	Yes	Yes	Yes	NA	No	No	6
2016	Chou, R. et al. [153]	Yes	Yes	Yes	Yes	Yes	Yes	Yes	No	NA	No	Yes	8
2016	Church, E. W. et al. [137]	No	No	Yes	No	No	No	Yes	Yes	Yes	Yes	No	5
2016	Hall, H. et al. [83]	Yes	Yes	Yes	No	Yes	Yes	Yes	Yes	NA	No	No	7
2016	Page Matthew, J. et al. [46]	Yes	Yes	Yes	Yes	Yes	Yes	Yes	Yes	NA	No	Yes	9
2016	Ruddock, J. K. et al. [84]	Yes	Yes	Yes	No	Yes	Yes	Yes	Yes	NA	No	No	7
2016	Ruffini, N. et al. [139]	No	Yes	Yes	Yes	Yes	Yes	Yes	Yes	NA	No	No	7
2016	Varatharajan, S. et al. [85]	Yes	No	No	No	No	Yes	Yes	No	NA	No	No	3
2016	Wearing, J. et al. [140]	No	Yes	Yes	No	No	Yes	Yes	Yes	NA	No	No	5
2016	Wong, J. J. et al. [86]	Yes	No	No	No	No	Yes	Yes	No	NA	No	No	3
2016	Yao, M. et al. [87]	Yes	Yes	Yes	Yes	No	Yes	Yes	Yes	NA	No	No	7
2015	Cicchintti L. et al. [116]	No	Yes	Yes	Yes	No	Yes	Yes	Yes	NA	No	Yes	7
2015	Franke, H. et al. [82]	No	Unclear	Yes	Yes	Yes	Yes	Yes	Yes	NA	No	No	6
2015	Gross A. J. et al. [36]	Yes	Yes	Yes	Yes	Yes	Yes	No	No	Yes	No	No	7
2015	Liddle S. D. & Pennick V. [35]	No	Yes	Yes	Yes	Yes	Yes	Yes	Yes	NA	No	Yes	8
2015	Posadzki, P. et al. [138]	Yes	No	Yes	No	No	Yes	Yes	No	NA	No	No	4
2015	Puentedura E. J. & O'Grady W. H. [134]	No	No	Yes	No	No	Yes	No	No	No	NA	No	2
2015	Southerst D. et al. [49]	Yes	Yes	Yes	Yes	No	Yes	Yes	Yes	NA	No	No	7
2015	Yuan Q.-L. et al. [48]	No	Unclear	Yes	Yes	No	Yes	Yes	Yes	NA	No	No	5
2015	Zhu L. et al. [47]	No	Yes	Yes	Yes	No	Yes	Yes	Yes	NA	No	No	6
2014	Bryans R. et al. [9]	No	No	Yes	No	No	Yes	Yes	No	Yes	No	No	4
2014	Clar C. et al. [101]	No	Yes	Yes	No	No	No	Yes	Yes	NA	No	No	4
2014	Close C. et al. [53]	Yes	No	Yes	Yes	No	Yes	Yes	Yes	NA	No	No	6
2014	Franke H. et al. [52]	No	Yes	Yes	Yes	Yes	Yes	Yes	Yes	NA	No	No	7
2014	Kizhakkeveettil A. et al. [51]	No	Yes	Yes	No	No	Yes	Yes	No	NA	No	No	4
2014	Page M. J. et al. [37]	Yes	Yes	Yes	Yes	Yes	Yes	Yes	Yes	NA	No	Yes	9
2014	Sutton D. et al. [50]	Yes	Yes	Yes	No	No	Yes	Yes	Yes	NA	No	No	6
2014	Todd A. J. et al. [98]	No	No	Yes	Yes	No	Yes	No	No	NA	No	No	3
2014	Tuchin P. [130]	No	No	Yes	No	No	Yes	No	No	NA	NA	No	2
2014	Yin P. et al. [93]	No	Yes	No	No	No	Yes	No	No	NA	NA	No	2
2014	Young J. L. et al. [105]	No	No	No	No	No	Yes	Yes	Yes	NA	No	No	3
2013	Brantingham J. W. et al. [97]	No	No	Yes	Yes	No	Yes	Yes	Yes	NA	No	No	5
2013	Hebert J. J. et al. [103]	No	No	Yes	No	No	Yes	No	No	NA	NA	No	2
2013	Huisman P. A. et al. [57]	No	No	Yes	No	Yes	Yes	Yes	Yes	NA	No	No	5
2013	Parkinson L. et al. [109]	No	Yes	No	No	No	Yes	Yes	Yes	NA	No	No	4
2013	Posadzki P. et al. [56]	Yes	Yes	Yes	No	No	Yes	Yes	Yes	NA	No	No	6
2013	Scholten-Peeters G. G. M. et al. [55]	No	Yes	Yes	No	No	Yes	Yes	Yes	NA	No	No	5
2013	Schroeder J. et al. [54]	No	No	No	No	Yes	Yes	Yes	Yes	NA	No	No	4
2013	Wynd S. et al. [126]	No	Yes	No	No	Yes	No	No	No	No	No	No	2
2013	Yang M. et al. [38]	Yes	Yes	Yes	No	Yes	Yes	Yes	Yes	NA	No	Yes	8
2012	Brantingham J. W. et al. [102]	No	No	No	No	No	Yes	Yes	Yes	NA	No	No	3
2012	Dobson D. et al. [40]	Yes	Yes	Yes	Yes	Yes	Yes	Yes	Yes	NA	No	Yes	9
2012	Furlan A. D. et al. [148, 149]	No	Yes	Yes	No	No	Yes	Yes	Yes	NA	No	No	5
2012	Gleberzon B. J. et al. [106]	No	No	Yes	No	No	Yes	Yes	Yes	NA	No	No	4
2012	Haynes M. J. et al. [125]	Yes	No	Yes	No	No	No	Yes	Yes	NA	NA	No	4
2012	Kuczynski J. J. et al. [60]	No	Yes	No	No	No	Yes	Yes	No	NA	No	No	3
2012	Lin J. H. et al. [59]	No	Yes	Yes	No	No	Yes	Yes	Yes	NA	No	No	5
2012	Posadzki P. & Ernst E. [58]	Yes	No	Yes	No	No	Yes	Yes	Yes	NA	No	No	5
2012	Puentedura E. J. et al. [123]	No	Yes	Yes	No	No	Yes	No	No	Yes	NA	No	4
2012	Rubinstein S. M. et al. [15, 39]	Yes	No	Yes	Yes	Yes	Yes	Yes	Yes	Yes	No	Yes	9
2012	Stuber K. A. et al. [132]	No	No	Yes	No	Yes	No	Yes	No	NA	NA	No	3
2011	Brantingham J. et al. [104]	No	No	No	No	No	Yes	Yes	Yes	NA	No	No	3
2011	Cross K. et al. [65]	No	Yes	Yes	No	Yes	Yes	Yes	Yes	NA	No	No	6
2011	Huang T. et al. [43]	Yes	Yes	Yes	No	Yes	Yes	Yes	Yes	NA	No	No	7
2011	Lystad R. P. et al. [112]	No	No	Yes	No	Yes	Yes	Yes	Yes	NA	No	No	5
2011	Posadzki P. & Ernst E. [63]	No	No	Yes	No	No	Yes	Yes	Yes	NA	No	No	4
2011	Posadzki P. & Ernst E. [62]	No	No	Yes	No	No	Yes	Yes	Yes	NA	No	No	4
2011	Posadzki P. & Ernst E. [61]	No	No	Yes	No	No	Yes	Yes	Yes	NA	No	Yes	5
2011	Posadzki P. & Ernst E. [64]	No	Yes	Yes	Yes	No	Yes	Yes	Yes	NA	No	No	6
2011	Rubinstein S. M. et al. [14, 42]	Yes	Yes	Yes	Yes	Yes	Yes	Yes	Yes	NA	No	Yes	9
2011	Walker B. F. et al. [41, 44]	Yes	Yes	Yes	Yes	Yes	Yes	Yes	Yes	NA	No	No	8
2010	Carlesso L. C. et al. [96]	No	Yes	No	Yes	No	Yes	Yes	Yes	Yes	No	No	6
2010	Carnes D. et al. [88]	No	No	Yes	No	No	No	Yes	No	No	No	No	2
2010	Ernst E. [128]	No	No	Yes	No	No	Yes	No	No	NA	NA	No	2
2010	Hahne A. J. et al. [66]	No	Yes	Yes	No	No	Yes	Yes	Yes	NA	No	No	5
2010	Kaminskyj A. et al. [108]	No	No	Yes	Yes	Yes	Yes	Yes	Yes	NA	No	No	6
2010	Shin B.-C. et al. [95]	No	Unclear	Yes	Yes	No	Yes	No	No	NA	NA	No	3
2009	Boudreau R. et al. [147]	No	No	No	No	No	Yes	No	No	NA	No	No	1
2009	Boudreau R. & Spry C. [151]	No	Unclear	No	No	Yes	Yes	No	No	NA	No	No	2
2009	Brurberg K. G. et al. [145]	No	No	Yes	No	Yes	No	Yes	Yes	NA	No	No	4
2009	Gouveia L. O. et al. [99]	No	No	Yes	No	No	Yes	No	No	No	NA	No	2
2009	Hunt K. J. et al. [67]	No	Yes	Yes	No	No	Yes	Yes	Yes	NA	No	No	5
2009	Khorsan B. et al. [94]	No	No	Yes	No	No	Yes	Yes	Yes	NA	No	No	4
2009	Reiman M. P. et al. [110]	No	No	Yes	Yes	No	Yes	Yes	Yes	NA	No	No	5
2008	Miley M. L. et al. [127]	No	No	No	No	No	No	No	No	No	No	No	0
2008	Stuber K. J. & Smith D. L. [107]	No	No	Yes	Yes	Yes	Yes	Yes	Yes	NA	No	No	6
2008	Vernon H. & Humphreys B. K. [68]	No	No	Yes	No	No	Yes	Yes	Yes	NA	No	No	4
2007	Chou R. & Huffman L. H. [141, 143]	No	Unclear	Yes	No	Yes	Yes	Yes	Yes	No	No	No	5
2007	Ernst E. [118]	No	No	Yes	No	No	Yes	No	No	NA	NA	No	2
2007	Gross A. R. et al. [71]	No	No	No	Yes	No	No	Yes	No	NA	No	No	2
2007	Hawk C. et al. [100]	No	No	Yes	No	No	Yes	Yes	Yes	NA	No	No	4
2007	Luijsterburg P. A. J. et al. [70]	No	No	No	No	No	Yes	Yes	Yes	NA	No	No	3
2007	Vernon H. & Humphreys B. K. [69]	No	Unclear	No	No	No	Yes	Yes	Yes	NA	No	No	3
2007	Vernon H. et al. [113]	No	No	Yes	No	Yes	Yes	Yes	Yes	NA	No	No	5
2006	Gemmell H. & Miller P. [111]	No	No	Yes	No	No	Yes	Yes	Yes	NA	No	No	4
2006	Proctor M. et al. [16]	Yes	Yes	Yes	Yes	Yes	Yes	Yes	Yes	NA	No	No	8
2006	Snelling N. J. [135]	No	No	Yes	No	No	Yes	Yes	Yes	NA	No	No	4
2005	Brown A. et al. [146]	No	Yes	Yes	No	Yes	Yes	Yes	Yes	NA	No	No	6
2005	Ernst E. [133]	No	No	Yes	No	No	Yes	No	No	NA	NA	No	2
2005	Hondras M. A. et al. [17]	No	Yes	Yes	Yes	Yes	Yes	Yes	Yes	NA	No	No	7
2005	Lisi A. J. et al. [120]	No	No	Yes	No	No	Yes	Yes	Yes	NA	No	No	4
2005	Rubinstein S. M. et al. [91]	No	Yes	Yes	No	No	Yes	No	No	NA	No	No	3
2004	Brønfort G. et al. [45]	Yes	Yes	Yes	No	Yes	Yes	Yes	Yes	NA	No	No	7
2004	Ernst E. [89]	No	No	Yes	No	No	Yes	No	No	NA	No	No	2
2004	Lenssinck M.-L. B. et al. [72]	No	Yes	Yes	No	Yes	Yes	Yes	Yes	NA	No	No	6
2004	Oduneye F. [152]	No	No	No	No	No	Yes	No	No	NA	No	No	1
2004	Oliphant D. [115]	No	No	Yes	No	No	Yes	Yes	No	No	No	No	3
2003	Ernst E. [90]	No	No	Yes	No	No	Yes	No	No	NA	No	No	2
2002	Ernst E. [119]	No	No	Yes	No	No	No	No	No	NA	No	No	1
2002	Ernst E. [136]	No	No	Yes	No	No	Yes	No	No	NA	NA	No	2
2002	Gerritsen A. A. M. et al. [74]	No	Yes	Yes	No	No	Yes	Yes	Yes	NA	No	No	5
2002	Gross A. R. et al. [142]	No	Yes	Yes	No	No	Yes	Yes	Yes	No	No	No	5
2002	Gross A. R. et al. [73]	No	Yes	No	No	No	Yes	Yes	Yes	NA	No	No	4
2002	Stevinson C. & Ernst E. [124]	No	No	Yes	No	No	Yes	No	No	No	No	No	2
2001	Bronfort G. et al. [76]	No	No	No	No	Yes	Yes	Yes	Yes	NA	No	No	4
2001	Ernst E. [129]	No	No	No	No	No	Yes	No	No	NA	No	No	1
2001	Ernst E. & Harkness E. [75]	No	No	Yes	No	No	Yes	Yes	Yes	NA	No	No	4
2000	Ernst E. [77]	No	No	No	No	No	Yes	No	No	NA	No	No	1
2000	Magee D. J. et al. [117]	No	Unclear	Yes	No	No	Yes	Yes	Yes	NA	No	No	4
1999	Fabio R. P. D. [121]	No	No	Yes	No	Yes	Yes	No	No	No	No	No	3
1999	Haldeman S. M. et al. [122]	No	No	Yes	No	No	Yes	No	No	NA	No	No	2
1999	Vernon H. et al. [78]	No	No	Yes	No	No	Yes	Yes	Yes	NA	No	No	4
1996	Aker P. D. et al. [79]	No	Unclear	Unclear	Yes	No	No	Yes	Yes	NA	No	No	3
1996	Assendelft W. J. J. et al. [131]	No	No	Unclear	Unclear	No	No	No	No	No	No	No	0
1996	Hurwitz E. L. et al. [144, 150]	No	No	No	No	No	No	Yes	Yes	No	No	No	2
1995	Dabbs V. & Lauretti W. J. [114]	No	No	No	No	No	Yes	No	No	No	No	No	1
1992	Shekelle P. G. et al. [92]	No	No	Yes	Yes	No	Yes	Yes	Yes	NA	No	No	5
	Total number of ‘yes’ for each item	25	47	92	33	35	105	89	78	6	1	10	

*AMSTAR* A Measurement Tool to Assess Systematic Reviews, *NA* not applicable

^a^The total score is the number of ‘yes’ for each review. It was calculated giving one point for each ‘yes’ given for the 11 items. 1. Was an 'a priori' design provided? 2. Was there duplicate study selection and data extraction? 3. Was a comprehensive literature search performed? 4. Was the status of publication (i.e. grey literature) used as an inclusion criterion? 5. Was a list of studies (included and excluded) provided? 6. Were the characteristics of the included studies provided? 7. Was the scientific quality of the included studies assessed and documented? 8. Was the scientific quality of the included studies used appropriately in formulating conclusions? 9. Were the methods used to combine the findings of studies for AEs appropriate? 10. Was the likelihood of publication bias assessed for AEs? 11. Was the conflict of interest included?

Furthermore, very few reviews rated the quality of the evidence for AEs and/or SAEs, with GRADE being the most frequently used tool.

### Serious adverse events

The estimates for the incidence of SAEs (Table 5) were heterogeneous, as they had different units (e.g. per number of manipulations, per visits or no unit), were based on different patient types, and were obtained from different types of studies [see Table, Additional file 7, showing which studies the estimates for the incidence of SAEs are based on].

**Table 5:** Estimates for the incidence of serious adverse events following spinal manipulative therapy

Year	Author	Estimates
2014	Todd A. J. et al. [98]	From a SR: 1 SAE in 250 million pediatric visits. From a discussion paper: 0 SAEs reported in >30,000 treatments by medical manipulators.
2010	Carnes D. et al. [88]	From a pCohort: 14 cases of ‘unbearably severe side effects’ in 4712 treatments (0.13%). Upper risk rate for ‘serious adverse events’ of approximately 0.01% (3/28,109 consultations). Their estimation from all pCohorts: Upper 95% CI incidence risk rate of major adverse events of 0.007% (0/42,451) after treatment or 0.01% (0/22,833) per patient. From RCTs: No ‘major adverse events’ in the 31 RCTs (which included 2281 participants who received manual therapy and 2779 who received other therapies). Upper incidence rate of major adverse events of ~0.13% (0/2301) after manual therapy treatment.
2009	Gouveia L. O. et al. [99]	Their own synthesis (*based on surveys*): Between 5 strokes in 100,000 manipulations to 1.46 SAEs in 10,000,000 manipulations and 2.68 deaths in 10,000,000 manipulations.
2008	Miley M. L. et al. [127]	From a CC (*which they consider the best available estimate*): Approximately 1.3 cases of VAD or occlusion attributable to CMT would be observed within 1 week of manipulative therapy for every 100,000 persons <45 years of age receiving CMT. From reviews: Published estimates of the incidence of VAD and stroke after range from 1 in 5.8 million to 1 in 5000.
2007	Chou R. & Huffman L. H. [141, 143]	From SRs: <1 SAE per 1 million patient visits.
2006	Snelling N. J. [135]	From a SR: 1 additional disc herniation or CES in 3.7 million manipulations (in pts, with lumbar disc herniation).
2004	Oliphant D. [115]	From their own estimation: <1 worsening LDH or CES in 3,72 million manipulations (in pts. with lumbar disc herniation), 1 worsening lumbar disc herniation or CES in 1,78 million manipulations (including manipulations under anesthesia; in pts. with lumbar disc herniation). From other reviews: 1 CES in 128 million manipulations (given the quality score 84%), 1 CES in 100 million manipulations (given the quality score 86%), <1 (CES or herniation) in 1 million manipulations (given the quality score 74%), 1 LDH or CES in 2,789,709 manipulations (1 LDH in 8,369,129 manipulations, and 1 CES in 4,184,564 manipulations) (given the quality score 32%). From a retrospective study: ‘They stated they were 95% confident that the risk of complication of manipulation for patients with back pain and sciatica was between 0% and 5%.’ From a prospective study: ‘A prospective evaluation of 2000 patients attending a chiropractic college clinic failed to reveal even one major complication’, ‘1000 new patients and 4700 treatments and found no permanent complications’. From surveys: 1 minor or transient complication but no serious or permanent complications in 38,137 lumbar spinal manipulations. From pooling the prospective and retrospective studies together: 0 major, serious, or permanent complications in >2100 patients (>13,100 treatments). 0 complications in 117 patients diagnosed as having LDH (>2000 spinal manipulation of probable disc herniations).
2002	Gross A. R. et al. [142]	From SRs: 1 serious complication in 20,000 to 5 serious complications in 10,000,000 cervical spine manipulations (rated as low accuracy and level V evidence), 1 stroke from cervical manipulation in 100,000 (0.001%). From a survey: 1 CVA in 228,050 manipulations, 1 CVA in 1.3 million, 5 CVA in one million.
2002	Stevinson C. & Ernst E. [124]	Their own summarisation: ‘Estimates of the incidence of serious complications range from 1 per 2 million manipulations to 1 per 400,000’. From reviews and a letter: 1 SAE per 1–2 million treatments. From surveys: 1 sleight neurologic complication per 40,000 manipulations, 1 severe complication per 400,000 manipulations, 1 stroke per 1,300,000 treatments of cervical SMT. From insurance claim data referred to in a SR: 1 stroke per 2 million manipulations. From a CC: 1.3 VBA within 1 week of treatment in 100,000 pts <45 years receiving chiropractic treatment.
1996	Assendelft W. J. J. et al. [131]	Their own conclusion (*partly based on the articles not appearing in their result section*): From 1 VBA in 20,000 patients to 1 VBA in 1 million cervical manipulations. <1 CES in 1 million treatments. From a SR: No complications in 1500 patients treated with manipulation in clinical trials. From surveys: 1 slight neurological complication in 40,000 cases, 1 important complication in 400,000 manipulative procedures, 1 VBA in 228,050 manipulations, <5 strokes in 100.000 patients receiving neck manipulations.
1996	Hurwitz E. L. et al. [150]	From their own estimation: 5–10 VBA or other complications (spinal cord compression, vertebral fracture, tracheal rupture, diaphragm paralysis, internal carotid hematoma, cardiac arrest) in 10,000,000 manipulations, 3–6 major impairment (paralysis, neurologic deficit, other permanent functional impairment) in 10,000,000 manipulations, <3 deaths in 10,000,000 manipulations. From surveys: 1 serious complication in 400,000 to >1 million manipulations, 1 CVA accident in 3.85 million cervical spine manipulations. *They compare the incidence rates with NSAID consumption (0.39–3.2 serious gastrointestinal event in 1000 subjects) and cervical spine surgery (15.6 neurologic complications (spinal cord or nerve root injury, recurrent laryngeal nerve palsy, dural leak, and injury to cervical sympathetic nerve trunk (Horner's syndrome)) in 1000 surgeries and 6.9 deaths in 1000 surgeries.*
1995	Dabbs V. & Lauretti W. J. [114]	Their own summarisation: 0.5–2 strokes in one million cervical manipulations performed, 1 serious vascular complication in 100.000 patients who undergo a course of treatment (10–15 sessions of cervical manipulation over the course of a year) with cervical manipulation, or 0.001%, 1 death in 400.000 pts. treated, or an ‘overall death rate of 0.0025% per course of treatment for patients with neck pain who are treated with cervical manipulation.’ *They compare this with a risk of 0.4% for getting serious gastrointestinal ulcers requiring hospitalization because of NSAID use, and a risk of 0.04% for death from gastrointestinal bleeding caused by NSAID use.* Their own calculation based on insurance company data: <1 stroke in 2 million cervical manipulations. From surveys: 1 serious complication in 400.000 cervical manipulations (no reported deaths), 1 complication in 518.000 manipulations, 1 stroke in 500.000 cervical manipulations, no serious incidence in >500.000 manipulations, 2–3 ‘more-or-less serious incidents’ in one million treatments. From reports: no vertebral artery injury or stroke in 5 million cervical manipulations, no significant complications in 168.000 cervical manipulations. From a review: 1–2 strokes in one million manipulations.
1992	Shekelle P. G. et al.[92]	Their own estimation: <1 case of CES in 100 million lumbar spinal manipulations.

*CC* case-control study, *CES* cauda equina syndrome, *CMT* cervical manipulative therapy, *CVA* cerebrovascular accident, *LDH* lumbar disc herniation, *NSAID* non-steroidal anti-inflammatory drug, *pCohort* prospective cohort study, *RCT* randomized controlled trial, *SAE* serious adverse event, *SMT* spinal manipulative therapy, *SR* systematic review, *VAD* vertebral artery dissection, *VBA* vertebrobasilar accident

When not distinguishing between the different types of SMT treatments and assuming that one treatment or visit equals one manipulation, and leaving out the minority of estimates not specifying the units or using per patient as the unit, the estimates for the incidence of SAEs ranges from 1 in 20,000 manipulations to 1 in 250,000,000 manipulations (Table 6).

**Table 6:** Estimates of the incidences of serious adverse events (some scaled for comparability)

Death	
1 in >3330.000–3,730,000 manipulations	
Stroke	
1 in 20,000–2,000,000 manipulations	
Vertebrobasilar accident (VBA)	
1 in 228,050–1,000,000 manipulations	
Cerebrovascular accident (CVA)	
1 in 228,050– 3,850,000 manipulations	
Lumbar disc herniation (LDH)	
1 in 8,369,129 manipulations^a^	
Cauda equina syndrome (CES)	
1 CES in >1,000,000–128,000,000 manipulations	
CES or LDH	
1 in >1,000,000–3,720,000 manipulations	
‘Serious adverse events’	
1 in 1,000,000–250,000,000 manipulations	
‘Serious complication’	
1 in 20,000–2,000,000 manipulations	

^a^Only one estimate was available

Based on the conclusions of the reviews regarding AEs and SAEs, 54 reviews (46%) expressed that SMT is safe, 15 (13%) expressed that SMT is harmful and 49 reviews (42%) were neutral or unclear regarding the safety of SMT, with a fair agreement between the two reviewers (Cohens Weighted Kappa, 0.50).

The calculations of RRs show a higher chance of a review communicating that SMT is safe, when having a higher methodological quality, compared to reviews of lower methodological quality (statistically significant for the AMSTAR items 5, 7 and 8; Table 7). And vice versa, there is a lower chance of a review communicating that SMT is harmful, when it has a lower methodological quality.

**Table 7:** The risk ratio of having the opinion that spinal manipulative therapy is safe or harmful, respectively, if a ‘yes’ was obtained in the individual AMSTAR items (118 reviews)

	Risk ratio (RR)					
	RR (95% CI) for communicating that SMT is safe		*P* values	RR (95% CI) for communicating that SMT is harmful		*P* values
AMSTAR #1	1.4	(1.0 to 2.1)	0.109	Not estimable^b^		–
AMSTAR #2	1.4	(1.0 to 2.1)	0.091	0.2	(0.1 to 1.0)	0.025
AMSTAR #3	1.0	(0.6 to 1.6)	0.964	1.8	(0.4 to 7.6)	0.386
AMSTAR #4	1.2	(0.8 to 1.8)	0.436	0.4	(0.1 to 1.7)	0.178
AMSTAR #5	1.8	(1.2 to 2.5)	0.005	0.2	(0.0 to 1.2)	0.038
AMSTAR #6	1.5	(0.7 to 3.6)	0.252	1.7	(0.2 to 12.1)	0.566
AMSTAR #7	3.2	(1.4 to 7.2)	<0.001	0.1	(0.0 to 0.2)	<0.001
AMSTAR #8	1.8	(1.1 to 3.0)	0.014	0.1	(0.0 to 0.3)	<0.001
AMSTAR #9	2.2	(0.8 to 5.8)	0.152	0.5	(0.1 to 3.9)	0.528
AMSTAR #10	2.0	(1.6 to 2.4)	0.331	Not estimable^a^		–
AMSTAR #11	1.6	(1.0 to 2.5)	0.109	Not estimable^b^		–

For descriptions of each AMSTAR item, see footnote for Table 4

*AMSTAR* A Measurement Tool to Assess Systematic Reviews, *CI* confidence interval, *RR* risk ratio, *SMT* spinal manipulative therapy

^a^No SRs had a ‘yes’ for this item

^b^No SRs had a ‘yes’ for this item and communicated ‘safe’

### Reviews specifically investigating adverse events

When only considering the subset of reviews, where the objective was to investigate AEs (37 reviews), then 8 reviews (22%) expressed that SMT is safe, 13 reviews (35%) expressed that SMT is harmful and 16 reviews (43%) were neutral or unclear regarding the safety of SMT. Hence, there is a tendency that a bigger proportion of these reviews are expressing that SMT is harmful compared to the full sample of reviews. The calculations of RRs did not obtain enough power to show any statistically significant RRs [see Table, Additional file 8, which shows the calculations of RRs]. The possibility of a causal relationship between SMT and SAEs was specifically investigated in six of the included reviews [89, 90, 118, 124, 127, 133] (Table 8). Five of these had for each case report or case series assessed the likelihood of causality [89, 90, 118, 124, 133]. In all cases, ‘certain’ was not the single most used rating. Miley et al. [127] used another approach and concluded weak to moderate strength of evidence for a causal relationship between cervical SMT and vertebral artery dissection, and expressed that comprehensive prospective studies are needed to further examine this relationship.

**Table 8:** Assessments of the likelihood of the causal relationship between spinal manipulative therapy and serious adverse events in reviews based on case reports and case series

Rating of causal relationship	Ernst 2007 [118]	Ernst 2005 [133]	Ernst 2004 [89]	Ernst 2003 [90]	Stevinson 2002 [124]
‘Certain’, *n* (%)	8 (21.6%)	6 (42.9%)	12 (30%)	0 (0%)	5 (22.7%)
‘Likely’, *n* (%)	18 (48.6%)	6 (42.9%)	16 (40%)	0 (0%)	14 (63.6%)
‘Possible’, *n* (%)	8 (21.6%)	2 (14.3%)	9 (22.5%)	2 (100%)	0 (0%)
‘Not assessable’ or ‘???’, *n* (%)	3 (8.2%)	0 (0%)	3 (7.5%)	0 (0%)	3 (13.6%)
Total, *n* (%)	37 (100%)	14 (100%)	40 (100%)	2 (100%)	22 (100%)

## Discussion

In this overview, the included reviews did not provide sufficient data for synthesis, and therefore it is currently not possible to provide an overall estimate for the risk of SAEs associated with SMT. Of the few reviews providing estimates for the incidence of SAEs, no reliable single estimate was provided, and it was not possible to identify any agreement regarding the safety of SMT across the included reviews. Interestingly, we found indications that reviews with higher methodological quality generally used language suggesting SMT to be safer (or less harmful). However, when analysing this across the reviews whose objective was to investigate safety, this could not be replicated. In the few reviews assessing the likelihood of a causal relationship between SMT and SAEs, this relationship was not in all cases certain. However, it should be noted that these assessments were based on case reports and case series, which cannot determine causality.

This overview is to our knowledge, the most comprehensive overview conducted on SMT, by including more than 100 reviews on SMT, and the only one with a sole focus on the safety aspects of SMT. Our intention was to provide an overview of all SAEs from SMT regardless of the indications for the treatment, but our overview especially covers patients with cervical pain, low back pain and headache, which were the most frequently studied populations. The most frequently mentioned AEs/SAEs across the 118 reviews ranged from minor events, such as soreness, to significant events, such as spinal cord injury and death. While some of these events may to a large extent be unpredictable [155] and have major impact on not only the individual but also the SMT provider and society, it is not possible to ascertain the risk-benefit balance based on the current evidence [156]. We strongly encourage efforts to illuminate the risk-benefit ratio reliably, since this would be of value when comparing SMT with other treatment options. Some of our included reviews indicate that NSAIDs involve a substantially higher risk of SAEs (including death) than SMT [114, 150], but they did not take into account the possible benefits.

General limitations in overviews are that recently published primary studies or studies not included in reviews cannot be included, the included reviews may overlap, and that the overviews rely on the methodological quality of the included reviews, which again rely on the methodological quality of the primary studies [157]. Considering the low methodological quality of the included reviews, the communicated opinions could possibly be influenced by the background of the authors [158], and by lack of independence between the reviews, i.e. several reviews were written by the same author. A major limitation of this overview was the limited data on AEs and SAEs hindering a synthesis. On the level of reviews, poor reporting of AEs is present [159]; however, even high quality reviews may fail to provide reliable estimates due to poor reporting in the primary studies, and this was frequently highlighted in the discussions of the included reviews. In primary studies, underreporting may be expected for retrospective studies or poorly controlled prospective studies. Including only RCTs would provide an insufficient population size for detecting SAEs reliably, and it has been shown that even in RCTs, AEs and SAEs are poorly reported [126, 160] and underreported [96, 161]. Gorrell et al. [162] found that out of 368 RCTs on SMT, only 140 (38%) reported on AEs. This under-reporting will directly affect the reviews including the studies resulting in a underestimation of the risk. On the other hand, over-reporting may be present, since the different study types (ranging from case reports to RCTs) provide various levels of evidence, and therefore confounding and chance cannot be ruled out as possible explaining factors for some of the observed SAEs associated with SMT.

Our methodological approach has limitations too. Our inclusion criteria were slightly heterogeneous across reviews. We relied on the definitions of SMT used by the review authors, which varied between the reviews. Some of the reviews mixed SMT with other interventions under a common category such as ‘manual treatment’ or ‘manipulation’ without reporting on only the SMT subgroup. Even when authors describe interventions such as SMT, these may not always include high-velocity, low-amplitude thrusts. In that case, the intervention is less likely to result in SAEs and may influence their and our conclusion about safety by making (high-velocity, low-amplitude thrust-type) SMT appear more safe. Further, we did not require a quality assessment to have been conducted for case reports, case series, cross-sectional studies and surveys, which may have facilitated the inclusion of reviews including only these types of studies. Our judgements regarding the expressed opinions in the reviews were not based on any criteria but based on subjective interpretation and therefore not reproducible even though there was fair agreement between the reviewers. Other limitations include the absence of a double study selection, data extraction and quality assessment, and a very brief protocol. These methodological compromises were taken due to limited time resources. However, our search strategy was broad, and we applied a thorough study selection making us confident that we have identified the vast majority of the relevant scientific literature on SMT and we find it unlikely that more thorough study selection and extraction procedures would result in different conclusions.

## Conclusions

This overview has indeed demonstrated how extensive the literature on SMT is. Unfortunately, the majority of reviews are non-systematic and of poor quality. The available evidence showed a broad range of communicated opinions and very variable estimates of SAE incidence. Reviews with less methodological flaws typically communicated that SMT may be safe; however, the methodological quality was in general low and the included reviews very heterogeneous. Furthermore, for the subset of reviews whose objective was to investigate safety, this could not be replicated. Research of high quality, with sufficient sample size and an appropriate comparison group is needed to obtain reliable risk estimates. Furthermore, reviews suggested that a causal relationship between SMT and SAEs was often not certain. However, the types of SAEs reported were indeed significant, sustaining that there is some risk present; sometimes SMT may even lead to death or permanent disability.

## Additional files

Additional file 1: Protocol for the overview. (PDF 283 kb)

Additional file 2: PRISMA harm checklist. (PDF 28 kb)

Additional file 3: Search strategy. (PDF 179 kb)

Additional file 4: Reference lists for the excluded reviews. (PDF 365 kb)

Additional file 5: Reference lists for the included reviews in the overview. (PDF 177 kb)

Additional file 6: Table showing further study characteristics including conclusions extracted from each reviews. (PDF 416 kb)

Additional file 7: Table showing which studies the estimates for the incidence of SAEs are based on. (DOCX 25 kb)

Additional file 8: Table showing the calculations of RRs of having the opinion that spinal manipulative therapy is safe or harmful, respectively, if a ‘yes’ was obtained in the individual AMSTAR items, for the 33 reviews, whose objective was to investigate adverse events. (PDF 186 kb)

## Abbreviations

AEs: Adverse events
AMSTAR: A measurement tool to assess systematic reviews
DARE: Cochrane database of abstracts of reviews of effects
HTA: Cochrane health technology assessment database
PROSPERO: The International Prospective Register of Systematic Reviews
RCTs: Randomized controlled trials
RRs: Risk ratios
SAEs: Serious adverse events
SMT: Spinal manipulative therapy
