# Supplementary material for: The risk associated with spinal manipulation: an overview of reviews
Source: Syst Rev. 2017 Mar 24;6:64. doi: 10.1186/s13643-017-0458-y (PMC5366149; doi:10.1186/s13643-017-0458-y)
Supplement: Supplementary file 1 — Protocol for the overview. (PDF 283 kb) [file 13643_2017_458_MOESM1_ESM.pdf]

Protocol for an overview of reviews of spinal manipulation interventions

Sabrina Mai Nielsen, MSc[1], Simon Tarp, MSc, PhD[1], Robin Christensen, MSc, PhD[1], Henning Bliddal, MD, DMSc[1], Marius Henriksen, PT, PhD[1,2]

Affiliations:

1: The Parker Institute, Copenhagen University Hospital, Bispebjerg Frederiksberg, Copenhagen, Denmark. http://www.parkerinst.dk/

2: Department of Physical and Occupational Therapy, Copenhagen University Hospital, Bispebjerg Frederiksberg, Copenhagen, Denmark.

Anticipated start date:

08/12-2015

Anticipated completion date:

12/02-2016

Corresponding Author:

Marius Henriksen, PT, PhD , Professor

Department of Physical and Occupational Therapy, and

The Parker Institute.

Copenhagen University Hospital, Bispebjerg og Frederiksberg.

Nordre Fasanvej 57DK-2000 Copenhagen Frederiksberg

Denmark

Phone: +45 3531 2251

E-mail: marius.henriksen@regionh.dk

Objectives

The aim of this overview of reviews is to elucidate and quantify the risk of adverse events (AEs) associated with spinal manipulation regardless of the indications for the treatment.

METHODS

Protocol and registration

This brief protocol was developed and registered in International Prospective Register of Systematic Reviews (PROSPERO: CRD42015030068) prior to the initiation of the overview.

Eligibility criteria

We will include official health technology assessment reports and peer-reviewed reviews of studies of any type (incl both cases and cohorts) that examine individuals receiving at least one spinal manipulation. No restrictions will be put on the age, nationality, gender, or health status of the population. The control can be sham, placebo, any, or none. Reporting the outcome(s) of interest is not a criterion for inclusion, but at least an abstract in English, Danish, Swedish or Norwegian needs to be available.

In order to ensure that the included reviews are 'systematic', a criterion for inclusion will be to include the following two items from the tool Assessment of Multiple Systematic Reviews (AMSTAR): *were two or more electronic sources searched?* and *was the scientific quality of the included studies assessed and documented?* [1,2]. Other overview authors have used similar approach [3,4].

Search methods for identification of studies

Five databases will be searched: Cochrane Database of Systematic Reviews, Cochrane Database of Abstracts of Reviews of Effects (DARE), Cochrane Health Technology Assessment Database (HTA), MEDLINE via PubMed and EMBASE via Ovid. No language or date restrictions will be placed on the search. The initial search strategy was developed for PubMed and adapted to the other databases. It consists of an intervention filter and review filter.

In addition, references lists of the included reviews, other relevant overviews of reviews and relevant national clinical guidelines will be checked to identify further potential reviews for inclusion.

Selection of studies

One reviewer (SMN) will screen titles and abstracts, and the same reviewer will subsequently screen full texts to identify relevant reviews for the overview. A second reviewer (MH) will be consulted when the basis for decision making is not clear.

Data extraction and management

One reviewer (SMN) will perform the data extraction for each review. When the basis for decision making is not clear, a second reviewer (MH) will be consulted.

The primary outcome is serious adverse events (SAEs) defined as conditions requiring hospital admission (and mortality), and the secondary outcomes will be any AEs reported. One reviewer (SMN) will rate the severity of the reported AEs. If the basis for rating AE severity is unclear, a second reviewer (HB) will be consulted.

Assessment of methodological quality of included reviews

One reviewer will assess the quality of each review using the tool 'A Measurement Tool to Assess Systematic Reviews' (AMSTAR) [1,2]. This consists of 11 criteria, which will be given the rating 'yes' (clearly done), 'can't answer' (unclear if completed), 'no' (clearly not done), or 'not applicable'.

Assessment of the quality of the evidence in reviews

The quality of the evidence in the reviews included will be assessed by extracting GRADE ratings if sufficient information is provided in the publication. If other quality measures were used, these will be reported.

Data synthesis and presentation

The study selection will be summarized in a flow diagram, and the characteristics and the result of the AMSTAR assessment for the reviews will be summarized in summary tables.

As recommended by the Cochrane Collaboration, this overview will as far as possible rely on the analyses reported in the included review and summarize these [5]. The AEs and SAEs will be summarized for each review by counts and descriptions. The proportion (number of patients receiving spinal manipulation experiences AEs or SAEs), incidence, risk ratio (RR) and odds ratio (OR) will be calculated when they are not extractable. When a review also includes trials on interventions, other than spinal manipulation, only results on the AEs and SAEs for the spinal manipulation will be used.

If sufficient data is available, the presentation of the results will include a stratification with respect to type of SAEs and/or type of studies included (RCTs, case reports, case series and cohorts, or prospective and retrospective).

Funding

This research is funded by the Association of Danish Physiotherapists and by The Oak Foundation. These had no role in the writing of the protocol and will have no role in the conduct of the review, analyses, and reporting of results.

Competing interests

MH is associated with the Association of Danish Physiotherapists that could benefit from this publication.

REFERENCES

1. Shea BJ, Hamel C, Wells GA, et al. AMSTAR is a reliable and valid measurement tool to assess the methodological quality of systematic reviews. *J Clin Epidemiol.* 2009;62(10):1013-1020.
2. Shea BJ, Grimshaw JM, Wells GA, et al. Development of AMSTAR: a measurement tool to assess the methodological quality of systematic reviews. *BMC Med Res Methodol.* 2007;7:10.
3. Mickan S, Tilson JK, Atherton H, Roberts NW, Heneghan C. Evidence of effectiveness of health care professionals using handheld computers: a scoping review of systematic reviews. *J Med Internet Res.* 2013;15(10):e212.
4. Bobrovitz N, Onakpoya I, Roberts N, Heneghan C, Mahtani KR. Protocol for an overview of systematic reviews of interventions to reduce unscheduled hospital admissions among adults. *BMJ Open.* 2015;5(8):e008269.
5. Becker LA, Oxman AD. Chapter 22: Overviews of reviews. *Higgins JPT, Green S (editors), Cochrane Handbook for Systematic Reviews of Interventions Version 5.1.0 (updated March 2011).* 2011.
