# Supplementary material for: The risk associated with spinal manipulation: an overview of reviews
Source: Syst Rev. 2017 Mar 24;6:64. doi: 10.1186/s13643-017-0458-y (PMC5366149; doi:10.1186/s13643-017-0458-y)
Supplement: Supplementary file 3 — Search strategy. (PDF 179 kb) [file 13643_2017_458_MOESM3_ESM.pdf]

Search Strategies

The initial search strategy was developed for PubMed and adapted to the other databases. It consists of an intervention filter and review filter:

PubMed:

(((manipulat*[Title/Abstract] AND spine[Title/Abstract]) OR (manipulat*[Title/Abstract] AND spinal[Title/Abstract]) OR (manipulat*[Title/Abstract] AND lumbar[Title/Abstract]) OR (manipulat*[Title/Abstract] AND back[Title/Abstract]) OR (manipulat*[Title/Abstract] AND neck [Title/Abstract]) OR (manipulat*[Title/Abstract] AND cervical[Title/Abstract]) OR (manipulat*[Title/Abstract] AND thrust[Title/Abstract]) OR (manipulat*[Title/Abstract] AND osteopath*[Title/Abstract])) OR "Manipulation, Chiropractic"[Mesh] OR "spinal adjustment*" OR chiropractic*)

NOT (animals NOT humans)

AND

(Cochrane[Title/Abstract] OR CENTRAL[Title/Abstract] OR MEDLINE[Title/Abstract] OR EMBASE[Title/Abstract] OR pubmed[Title/Abstract] OR search*[Title/Abstract] OR “systematic review”[Title/Abstract] OR meta-analysis[Title/Abstract] OR metaanalysis[Title/Abstract] OR “network meta-analysis”[Title/Abstract] OR “Comparative effectiveness”[Title/Abstract] OR “Indirect comparison”[Title/Abstract] OR “mixed treatment comparison”[Title/Abstract] OR “Systematic Literature”[Title/Abstract])

Cochrane Database of Systematic Reviews, DARE and HTA:

#1 MeSH descriptor: [Manipulation, Chiropractic] explode all trees

#2 "spinal adjustment" and "spinal adjustments" or chiropracti*

#3 (manipulat* and spine) or (manipulat* and spinal) or (manipulat* and lumbar) or (manipulat* and back) or (manipulat* and neck) or (manipulat* and cervical) or (manipulat* and thrust) or (manipulat* and osteopath*)

#4 #1 or #2 or #3

EMBASE:

1. (animals not humans).mp. [mp=title, abstract, heading word, drug trade name, original title, device manufacturer, drug manufacturer, device trade name, keyword]

2. ((manipulat* and spine) or (manipulat* and spinal) or (manipulat* and lumbar) or (manipulat* and back) or (manipulat* and neck) or (manipulat* and cervical) or (manipulat* and thrust) or (manipulat* and osteopath*)).ti,ab.

3. ("spinal adjustment" or "spinal adjustments" or chiropracti*).ti,ab.

4. (Cochrane or CENTRAL or MEDLINE or EMBASE or pubmed or search* or "systematic review" or meta-analysis or metaanalysis or "network meta-analysis" or "Comparative effectiveness" or "Indirect comparison" or "mixed treatment comparison" or "Systematic Literature").ti,ab.

5. ((2 or 3) and 4) not 1

6. (conference).pt.

7. 5 not 6
