# Supplementary material for: The risk associated with spinal manipulation: an overview of reviews
Source: Syst Rev. 2017 Mar 24;6:64. doi: 10.1186/s13643-017-0458-y (PMC5366149; doi:10.1186/s13643-017-0458-y)
Supplement: Supplementary file 4 — Reference lists for the excluded reviews. (PDF 365 kb) [file 13643_2017_458_MOESM4_ESM.pdf]

Reference Lists for the Excluded Reviews

Content

Does not include studies on SMT (159 records and 15 records*)
Not clear if SMT was included (27 records and 4 records*)
Primary research (31 records and 1 record*)
Non-systematic (301 records and 13 records*)
Language (16 records and no records*)
No full-text or abstract available in English, Swedish, Norwegian or Danish (3 records)
English abstract available, but provides too few details (13 records)
Not retrievable (32 records and 8 records*)
Only protocol available (22 records and 8 records*)
Withdrawn (6 records and no records*)
Not possible to retrieve (4 records and no records*)
An update exists (12 records and no records*)
Not relevant for other reasons (6 records and 1 record*)

**records from the updated search*

Does not include studies on SMT (159 records and 15 records*)

1. Aggarwal A, Kumar S, Kumar R: **Therapeutic management of the hallux rigidus.** *Rehabil Res Pract* 2012, **2012**.
2. Almeida Matheus O, Silva Brenda NG, Andriolo Régis B, Atallah Álvaro N, Peccin Maria S: **Conservative interventions for treating exercise-related musculotendinous, ligamentous and osseous groin pain.** In *Cochrane Database Syst Rev*: John Wiley & Sons, Ltd; 2013.
3. Arroyo-Anllo EM, Pluchon C, Gil R: **Rehabilitation of spatial neglect. [Spanish].** *Psiquiatria Biologica* 2007, **14:**98-107.
4. Astin JA, Marie A, Pelletier KR, Hansen E, Haskell WL: **A review of the incorporation of complementary and alternative medicine by mainstream physicians.** *Arch Intern Med* 1998, **158:**2303-2310.
5. Au JK, Adam SI, Michaelides EM: **Contemporary management of pediatric lateral sinus thrombosis: a twenty year review.** *Am J Otolaryngol* 2013, **34:**145-150.
6. Bennett C, Underdown A, Barlow J: **Massage for promoting mental and physical health in typically developing infants under the age of six months.** In *Cochrane Database Syst Rev*: John Wiley & Sons, Ltd; 2013.
7. Bennett Michael H, Lehm Jan P, Mitchell Simon J, Wasiak J: **Recompression and adjunctive therapy for decompression illness.** In *Cochrane Database Syst Rev*: John Wiley & Sons, Ltd; 2012.
8. Bervoets DC, Luijsterburg PA, Alessie JJ, Buijs MJ, Verhagen AP: **Massage therapy has short-term benefits for people with common musculoskeletal disorders compared to no treatment: a systematic review.** *J Physiother* 2015, **61:**106-116.
9. Bettany-Saltikov J, Weiss H-R, Chockalingam N, Taranu R, Srinivas S, Hogg J, Whittaker V, Kalyan Raman V, Arnell T: **Surgical versus non-surgical interventions in people with adolescent idiopathic scoliosis.** In *Cochrane Database Syst Rev*: John Wiley & Sons, Ltd; 2015.

10. Boocock MG, McNair PJ, Larmer PJ, Armstrong B, Collier J, Simmonds M, Garrett N: **Interventions for the prevention and management of neck/upper extremity musculoskeletal conditions: a systematic review.** *Occup Environ Med* 2007, **64:**291-303.
11. Brady-Fryer B, Wiebe N, Lander Janice A: **Pain relief for neonatal circumcision.** In *Cochrane Database Syst Rev*: John Wiley & Sons, Ltd; 2004.
12. Brand PL, Engelbert RH, Helders PJ, Offringa M: **[Systematic review of the effects of therapy in infants with the KISS-syndrome (kinetic imbalance due to suboccipital strain)].** *Ned Tijdschr Geneeskd* 2005, **149:**703-707.
13. Brand PLP, Engelbert RHH, Helders PJM, Offringa M: **Systematic review of the effects of therapy in infants with the KISS-syndrome (kinetic imbalance due to suboccipital strain). [Dutch].** *Ned Tijdschr Geneeskd* 2005, **149:**703-707.
14. Cao H, Yang G, Wang Y, Liu Jian P, Smith Caroline A, Luo H, Liu Y: **Complementary therapies for acne vulgaris.** In *Cochrane Database Syst Rev*: John Wiley & Sons, Ltd; 2015.
15. Carneiro Machado LA, Sperling de Souza M, Ferreira PH, Ferreira ML: **The McKenzie method for low back pain: a systematic review of the literature with a meta-analysis approach.** *Spine (Phila Pa 1976)* 2006, **31:**E254-e262.
16. Chu J, Allen DD, Pawlowsky S, Smoot B: **Peripheral response to cervical or thoracic spinal manual therapy: an evidence-based review with meta analysis.** *J Man Manip Ther* 2014, **22:**220-229.
17. Coggrave M, Norton C, Cody June D: **Management of faecal incontinence and constipation in adults with central neurological diseases.** In *Cochrane Database Syst Rev*: John Wiley & Sons, Ltd; 2014.
18. Cogo E, Sampson M, Ajiferuke I, Manheimer E, Campbell K, Daniel R, Moher D: **Searching for controlled trials of complementary and alternative medicine: a comparison of 15 databases.** *Evid Based Complement Alternat Med* 2011, **2011**.
19. Conable KM, Rosner AL: **A narrative review of manual muscle testing and implications for muscle testing research.** *J Chiropr Med* 2011, **10:**157-165.
20. Cook C, Hegedus EJ, Ramey K: **Physical therapy exercise intervention based on classification using the patient response method: A systematic review of the literature.** *J Man Manip Ther* 2005, **13:**152-162.
21. Cooper KL, Harris PE, Relton C, Thomas KJ: **Prevalence of visits to five types of complementary and alternative medicine practitioners by the general population: a systematic review.** *Complement Ther Clin Pract* 2013, **19:**214-220.
22. Cramp F, Bottrell O, Campbell H, Ellyatt P, Smith C, Wilde B: **Non-surgical management of piriformis syndrome: a systematic review.** *Phys Ther Rev* 2007, **12:**66-72.
23. Crocker T, Forster A, Young J, Brown L, Ozer S, Smith J, Green J, Hardy J, Burns E, Glidewell E, Greenwood Darren C: **Physical rehabilitation for older people in long-term care.** In *Cochrane Database Syst Rev*: John Wiley & Sons, Ltd; 2013.
24. Day JM, McKeon P, Nitz A: **The efficacy of cervical/thoracic active range of motion for detecting changes associated with individuals receiving muscle energy techniques.** *Phys Ther Rev* 2010, **15:**453-461.
25. Day JM, Nitz AJ: **The effect of muscle energy techniques on disability and pain scores in individuals with low back pain.** *J Sport Rehabil* 2012, **21:**194-198.
26. Denneny D, Petersen K, McLoughlin R, Brook S, Hassan S, Williams Amanda CdC: **Trigger point manual therapy for the treatment of chronic non-cancer pain in adults.** In *Cochrane Database Syst Rev*: John Wiley & Sons, Ltd; 2015.
27. Dobscha SK, Clark ME, Morasco BJ, Freeman M, Campbell R, Helfand M: **Systematic review of the literature on pain in patients with polytrauma including traumatic brain injury** *Pain Med* 2009, **10:**1200-1217.
28. Donken Christian CMA, Al-Khateeb H, Verhofstad Michael HJ, van Laarhoven Cornelis JHM: **Surgical versus conservative interventions for treating ankle fractures in adults.** In *Cochrane Database Syst Rev*: John Wiley & Sons, Ltd; 2012.

29. Dubin JC, Comeau D, McClelland RI, Dubin RA, Ferrel E: **Lateral and syndesmotic ankle sprain injuries: a narrative literature review.** *J Chiropr Med* 2011, **10:**204-219.
30. Elders LA, Beek AJ, Burdorf A: **Return to work after sickness absence due to back disorders: a systematic review on intervention strategies.** *Int Arch Occup Environ Health* 2000, **73:**339-348.
31. Ernst E: **The "Activator": A systematic review of randomised clinical trials. [German].** *Perfusion* 2009, **22:**51-53.
32. Ernst E: **Chiropractic spinal manipulation for whiplash injury? A systematic review of controlled clinical trials.** *Focus Altern Complement Ther* 2009, **14:**85-86.
33. Ezzo J, Donner T, Nickols D, Cox M: **Is massage useful in the management of diabetes: a systematic review.** *Diabetes Spectr* 2001, **14:**218-225.
34. Fischbacher C: **Outpatient physiotherapy services for low back pain (Structured abstract).** In *Health Technology Assessment Database*. pp. 8; 2002:8.
35. Franke H, Fryer G, Ostelo Raymond WJG, Kamper Steven J: **Muscle energy technique for non-specific low-back pain.** In *Cochrane Database Syst Rev*: John Wiley & Sons, Ltd; 2015.
36. Franke H, Hoesele K: **Osteopathic manipulative treatment (OMT) for lower urinary tract symptoms (LUTS) in women.** *J Bodyw Mov Ther* 2013, **17:**11-18.
37. Fryer G, Morris T, Gibbons P: **Paraspinal muscles and intervertebral dysfunction: part one.** *J Manipulative Physiol Ther* 2004, **27:**267-274.
38. Fuhr AW, Menke JM: **Status of activator methods chiropractic technique, theory, and practice.** *J Manipulative Physiol Ther* 2005, **28:**e1-e20.
39. Furlan AD, Brosseau L, Imamura M, Irvin E: **Massage for low-back pain: a systematic review within the framework of the Cochrane Collaboration Back Review Group.** *Spine (Phila Pa 1976)* 2002, **27:**1896-1910.
40. Furlan AD, Brosseau L, Welch V, Wong J: **Massage for low back pain.** In *Cochrane Database Syst Rev*, 2000/10/18 edition; 2000.
41. Furlan AD, Imamura M, Dryden T, Irvin E: **Massage for low back pain: an updated systematic review within the framework of the Cochrane Back Review Group.** *Spine (Phila Pa 1976)* 2009, **34:**1669-1684.
42. Furlan Andrea D, Giraldo M, Baskwill A, Irvin E, Imamura M: **Massage for low-back pain.** In *Cochrane Database Syst Rev*: John Wiley & Sons, Ltd; 2015.
43. Gillespie Lesley D, Robertson MC, Gillespie William J, Sherrington C, Gates S, Clemson Lindy M, Lamb Sarah E: **Interventions for preventing falls in older people living in the community.** In *Cochrane Database Syst Rev*: John Wiley & Sons, Ltd; 2012.
44. Graham N, Gross A, Goldsmith Charles H, Klaber Moffett J, Haines T, Burnie Stephen J, Peloso Paul Michael J: **Mechanical traction for neck pain with or without radiculopathy.** In *Cochrane Database Syst Rev*: John Wiley & Sons, Ltd; 2008.
45. Graham N, Gross AR, Carlesso LC, Santaguida PL, Macdermid JC, Walton D, Ho E: **An ICON Overview on Physical Modalities for Neck Pain and Associated Disorders.** *Open Orthop J* 2013, **7:**440-460.
46. Gray K, Pacey V, Gibbons P, Little D, Burns J: **Interventions for congenital talipes equinovarus (clubfoot).** In *Cochrane Database Syst Rev*: John Wiley & Sons, Ltd; 2014.
47. Green C, Martin CW, Bassett K, Kazanjian A: **A systematic review and critical appraisal of the scientific evidence on craniosacral therapy (Structured abstract).** In *Database of Abstracts of Reviews of Effects*. pp. 54: University of British Columbia, Centre for Health Services and Policy Research, B.C. Office of Health Technology Assessment (BCOHTA); 1999:54.
48. Gunduz H, Binak DF: **Autonomic dysreflexia: an important cardiovascular complication in spinal cord injury patients.** *Cardiol J* 2012, **19:**215-219.
49. Hall S, Lewith G, Brien S, Little P: **A review of the literature in applied and specialised kinesiology.** *Forsch Komplementmed* 2008, **15:**40-46.
50. Handoll Helen HG, Madhok R: **Closed reduction methods for treating distal radial fractures in adults.** In *Cochrane Database Syst Rev*: John Wiley & Sons, Ltd; 2003.

51. Haneline M, Cooperstein R, Young M, Birkeland K: **An annotated bibliography of spinal motion palpation reliability studies.** *J Can Chiropr Assoc* 2009, **53:**40-58.
52. Haneline M, Lewkovich GN: **A narrative review of pathophysiological mechanisms associated with cervical artery dissection.** *J Can Chiropr Assoc* 2007, **51:**146-157.
53. Haneline MT, Cooperstein R, Young M, Birkeland K: **Spinal motion palpation: a comparison of studies that assessed intersegmental end feel vs excursion.** *J Manipulative Physiol Ther* 2008, **31:**616-626.
54. Harrison DE, Harrison DD, Colloca CJ, Betz J, Janik TJ, Holland B: **Repeatability over time of posture, radiograph positioning, and radiograph line drawing: an analysis of six control groups.** *J Manipulative Physiol Ther* 2003, **26:**87-98.
55. Hartvigsen L, Kongsted A, Hestbaek L: **Clinical examination findings as prognostic factors in low back pain: a systematic review of the literature.** *Chiropr Man Therap* 2015, **23:**13.
56. Hegedus EJ, Goode A, Butler RJ, Slaven E: **The neurophysiological effects of a single session of spinal joint mobilization: does the effect last?** *J Man Manip Ther* 2011, **19:**143-151.
57. Herd CR, Meserve BB: **A systematic review of the effectiveness of manipulative therapy in treating lateral epicondylalgia.** *J Man Manip Ther* 2008, **16:**225-237.
58. Heymans Martijn W, van Tulder Maurits W, Esmail R, Koes Bart W, Poquet N, Maher Christopher G, Lin Chung-Wei C: **Back schools for acute and subacute non-specific low-back pain.** In *Cochrane Database Syst Rev*: John Wiley & Sons, Ltd; 2010.
59. Heymans MW, van Tulder MW, Esmail R, Bombardier C, Koes BW: **Back schools for non-specific low-back pain.** In *Cochrane Database Syst Rev*, 2004/10/21 edition; 2004.
60. Heymans MW, van Tulder MW, Esmail R, Bombardier C, Koes BW: **Back schools for nonspecific low back pain: a systematic review within the framework of the Cochrane Collaboration Back Review Group.** *Spine (Phila Pa 1976)* 2005, **30:**2153-2163.
61. Hollerwoger D: **Methodological quality and outcomes of studies addressing manual cervical spine examinations: a review.** *Man Ther* 2006, **11:**93-98.
62. Hoskins W, Pollard H, Reggars J, Vitiello A, Bonello R: **Journal publications by Australian chiropractic academics: are they enough?** *Chiropr Osteopat* 2006, **14:**13.
63. Huggins T, Boras AL, Gleberzon BJ, Popescu M, Bahry LA: **Clinical effectiveness of the activator adjusting instrument in the management of musculoskeletal disorders: a systematic review of the literature.** *J Can Chiropr Assoc* 2012, **56:**49-57.
64. Hurlbert RJ: **Strategies of medical intervention in the management of acute spinal cord injury.** *Spine (Phila Pa 1976)* 2006, **31:**S16-21; discussion S36.
65. ICER: **Management options for low back disorders (Structured abstract).** In *Health Technology Assessment Database*: Institute for Clinical and Economic Review; 2011.
66. Johnson CD, Green BN: **Diversity in the chiropractic profession: preparing for 2050.** *J Chiropr Educ* 2012, **26:**1-13.
67. Johnson CD, Green BN, Nelson RC, Moreau B, Nabhan D: **Chiropractic and concussion in sport: a narrative review of the literature.** *J Chiropr Med* 2013, **12:**216-229.
68. Jungheim M, Miller S, Ptok M: **[Clicking larynx syndrome : a literature review and multiple-case report].** *HNO* 2013, **61:**965-969.
69. Jäkel A, von Hauenschild P: **Therapeutic effects of cranial osteopathic manipulative medicine: a systematic review.** *J Am Osteopath Assoc* 2011, **111:**685-693.
70. Karpouzis F, Bonello R, Pollard H: **Chiropractic care for paediatric and adolescent Attention-Deficit/Hyperactivity Disorder: A systematic review.** *Chiropr Osteopat* 2010, **18:**13.
71. Katalinic Owen M, Harvey Lisa A, Herbert Robert D, Moseley Anne M, Lannin Natasha A, Schurr K: **Stretch for the treatment and prevention of contractures.** In *Cochrane Database Syst Rev*: John Wiley & Sons, Ltd; 2010.
72. Kendall JC, Hartvigsen J, Azari MF, French SD: **Effects of Nonpharmacological Interventions for Dizziness in Older People: Systematic Review.** *Phys Ther* 2015.

73. Kronenberg F, Fugh-Berman A: **Complementary and alternative medicine for menopausal symptoms: a review of randomized, controlled trials.** *Ann Intern Med* 2002, **137:**805-813.
74. Krul M, van der Wouden Johannes C, van Suijlekom-Smit Lisette WA, Koes Bart W: **Manipulative interventions for reducing pulled elbow in young children.** In *Cochrane Database Syst Rev*: John Wiley & Sons, Ltd; 2012.
75. Kwan I, Bhattacharya S, Knox F, McNeil A: **Pain relief for women undergoing oocyte retrieval for assisted reproduction.** In *Cochrane Database Syst Rev*: John Wiley & Sons, Ltd; 2013.
76. Levack William MM, Weatherall M, Hay-Smith EJC, Dean Sarah G, McPherson K, Siegert Richard J: **Goal setting and strategies to enhance goal pursuit for adults with acquired disability participating in rehabilitation.** In *Cochrane Database Syst Rev*: John Wiley & Sons, Ltd; 2015.
77. Liddle SD, Gracey JH, Baxter GD: **Advice for the management of low back pain: a systematic review of randomised controlled trials.** *Man Ther* 2007, **12:**310-327.
78. Lin Chung-Wei C, Donkers Nicole AJ, Refshauge Kathryn M, Beckenkamp Paula R, Khera K, Moseley Anne M: **Rehabilitation for ankle fractures in adults.** In *Cochrane Database Syst Rev*: John Wiley & Sons, Ltd; 2012.
79. Lincoln AE, Vernick JS, Ogaitis S, Smith GS, Mitchell CS, Agnew J: **Interventions for the primary prevention of work-related carpal tunnel syndrome.** *Am J Prev Med* 2000, **18:**37-50.
80. Linko E, Harilainen A, Malmivaara A, Seitsalo S: **Surgical versus conservative interventions for anterior cruciate ligament ruptures in adults.** In *Cochrane Database Syst Rev*: John Wiley & Sons, Ltd; 2005.
81. Lærum E, Dullerud R, Grundnes O, Haagesen Ø, Indahl A, Ljunggren AE, Magnæs B, Nygaard Ø, Salvesen R, Kjønniksen I: **Treatment of lumbar disc herniation** In *Health Technology Assessment Database*. Senter for medisinsk metodevurdering; Oslo: Norwegian Knowledge Centre for the Health Services (NOKC); 2001.
82. Macedo LG, Maher CG, Latimer J, McAuley JH: **Motor control exercise for persistent, nonspecific low back pain: a systematic review.** *Phys Ther* 2009, **89:**9-25.
83. Machado L, Lin Chung-Wei C, Clare H, van Tulder Maurits W: **The McKenzie method for (sub)acute non-specific low-back pain.** In *Cochrane Database Syst Rev*: John Wiley & Sons, Ltd; 2012.
84. Machado L, van Tulder Maurits W, Lin Chung-Wei C, Clare H, Hayden Jill A: **The McKenzie method for chronic non-specific low-back pain.** In *Cochrane Database Syst Rev*: John Wiley & Sons, Ltd; 2012.
85. May S, Johnson R: **Stabilisation exercises for low back pain: a systematic review.** *Physiotherapy* 2008, **94:**179-189.
86. McNeely ML, Armijo Olivo S, Magee DJ: **A systematic review of the effectiveness of physical therapy interventions for temporomandibular disorders.** *Phys Ther* 2006, **86:**710-725.
87. MedicalAdvisorySecretariat: **Spinal cord stimulation for neuropathic pain: an evidence-based analysis (Structured abstract).** In *Health Technology Assessment Database*. pp. 80: Medical Advisory Secretariat, Ontario Ministry of Health and Long-Term Care (MAS); 2005:80.
88. Mootz RD, Coulter ID, Hansen DT: **Health services research related to chiropractic: review and recommendations for research prioritization by the chiropractic profession.** *J Manipulative Physiol Ther* 1997, **20:**201-217.
89. Morgan Angela T, Dodrill P, Ward Elizabeth C: **Interventions for oropharyngeal dysphagia in children with neurological impairment.** In *Cochrane Database Syst Rev*: John Wiley & Sons, Ltd; 2012.
90. Mrozek JP, Till H, Taylor-Vaisey AL, Wickes D: **Research in chiropractic education: an update.** *J Manipulative Physiol Ther* 2006, **29:**762-773.
91. Murphy LS, Reinsch S, Najm WI, Dickerson VM, Seffinger MA, Adams A, Mishra SI: **Spinal palpation: The challenges of information retrieval using available databases.** *J Manipulative Physiol Ther* 2003, **26:**374-382.
92. Murphy RJ, Carr AJ: **Shoulder pain.** *BMJ Clin Evid* 2010, **2010**.

93. Myers CD: **Complementary and Alternative Medicine for Persistent Facial Pain.** *Dent Clin North Am* 2007, **51:**263-274.
94. Myers CD, White BA, Heft MW: **A review of complementary and alternative medicine use for treating chronic facial pain.** *J Am Dent Assoc* 2002, **133:**1189-1196; quiz 1259-1160.
95. Oosterhuis T, Costa Leonardo OP, Maher Christopher G, de Vet Henrica CW, van Tulder Maurits W, Ostelo Raymond WJG: **Rehabilitation after lumbar disc surgery.** In *Cochrane Database Syst Rev*: John Wiley & Sons, Ltd; 2014.
96. Ostelo RW, de Vet HC, Waddell G, Kerckhoffs MR, Leffers P, van Tulder M: **Rehabilitation following first-time lumbar disc surgery: a systematic review within the framework of the cochrane collaboration.** *Spine (Phila Pa 1976)* 2003, **28:**209-218.
97. Ostelo RW, de Vet HC, Waddell G, Kerckhoffs MR, Leffers P, van Tulder MW: **Rehabilitation after lumbar disc surgery.** In *Cochrane Database Syst Rev*, 2002/06/22 edition; 2002.
98. Oto B, Corey DJ, 2nd, Oswald J, Sifford D, Walsh B: **Early Secondary Neurologic Deterioration After Blunt Spinal Trauma: A Review of the Literature.** *Acad Emerg Med* 2015, **22:**1200-1212.
99. Owen JM, Green BN: **Homeopathic treatment of headaches: a systematic review of the literature.** *J Chiropr Med* 2004, **3:**45-52.
100. Page Matthew J, O'Connor D, Pitt V, Massy-Westropp N: **Exercise and mobilisation interventions for carpal tunnel syndrome.** In *Cochrane Database Syst Rev*: John Wiley & Sons, Ltd; 2012.
101. Patel Kinjal C, Gross A, Graham N, Goldsmith Charles H, Ezzo J, Morien A, Peloso Paul Michael J: **Massage for mechanical neck disorders.** In *Cochrane Database Syst Rev*: John Wiley & Sons, Ltd; 2012.
102. Patterson J: **Spinal manipulation for chronic low back pain (Structured abstract).** In *Health Technology Assessment Database*. pp. 8; 2003:8.
103. Peters S, Page Matthew J, Coppieters Michel W, Ross M, Johnston V: **Rehabilitation following carpal tunnel release.** In *Cochrane Database Syst Rev*: John Wiley & Sons, Ltd; 2013.
104. Pfefer MT, Cooper SR, Uhl NL: **Chiropractic management of tendinopathy: a literature synthesis.** *J Manipulative Physiol Ther* 2009, **32:**41-52.
105. Pillai Riddell Rebecca R, Racine Nicole M, Gennis Hannah G, Turcotte K, Uman Lindsay S, Horton Rachel E, Ahola Kohut S, Hillgrove Stuart J, Stevens B, Lisi Diana M: **Non-pharmacological management of infant and young child procedural pain.** In *Cochrane Database Syst Rev*: John Wiley & Sons, Ltd; 2015.
106. Piper S, Shearer HM, Cote P, Wong JJ, Yu H, Varatharajan S, Southerst D, Randhawa KA, Sutton DA, Stupar M, et al: **The effectiveness of soft-tissue therapy for the management of musculoskeletal disorders and injuries of the upper and lower extremities: A systematic review by the Ontario Protocol for Traffic Injury management (OPTIMa) collaboration.** *Man Ther* 2015.
107. Pollock A, Farmer Sybil E, Brady Marian C, Langhorne P, Mead Gillian E, Mehrholz J, van Wijck F: **Interventions for improving upper limb function after stroke.** In *Cochrane Database Syst Rev*: John Wiley & Sons, Ltd; 2014.
108. Prabhu Rama KR, Swaminathan N, Harvey Lisa A: **Passive movements for the treatment and prevention of contractures.** In *Cochrane Database Syst Rev*: John Wiley & Sons, Ltd; 2013.
109. Puhl AA, Reinhart CJ, Rok ER, Injeyan HS: **An examination of the observed placebo effect associated with the treatment of low back pain - a systematic review.** *Pain Res Manag* 2011, **16:**45-52.
110. Rada G, Capurro D, Pantoja T, Corbalán J, Moreno G, Letelier Luz M, Vera C: **Non-hormonal interventions for hot flushes in women with a history of breast cancer.** In *Cochrane Database Syst Rev*: John Wiley & Sons, Ltd; 2010.
111. Randhawa K, Bohay R, Cote P, van der Velde G, Sutton D, Wong JJ, Yu H, Southerst D, Varatharajan S, Mior S, et al: **The Effectiveness of Non-invasive Interventions for Temporomandibular Disorders: A Systematic Review by the Ontario Protocol for Traffic Injury Management (OPTIMa) Collaboration.** *Clin J Pain* 2015.

112. Reinink H, Wegner I, Stegeman I, Grolman W: **Rapid systematic review of repeated application of the Epley maneuver for treating posterior BPPV (Provisional abstract).** In *Database of Abstracts of Reviews of Effects*. pp. 399-406; 2014:399-406.
113. Renner R-M, Jensen Jeffrey TJ, Nichols Mark DN, Edelman A: **Pain control in first trimester surgical abortion.** In *Cochrane Database Syst Rev*: John Wiley & Sons, Ltd; 2009.
114. Robertson C, Saratsiotis J: **A review of compressive ulnar neuropathy at the elbow.** *J Manipulative Physiol Ther* 2005, **28:**345.
115. Robinson N, Lorenc A, Liao X: **The evidence for Shiatsu: a systematic review of Shiatsu and acupressure.** *BMC Complement Altern Med* 2011, **11:**88.
116. Romano M, Negrini S: **Manual therapy as a conservative treatment for adolescent idiopathic scoliosis: a systematic review.** *Scoliosis* 2008, **3**.
117. Rome K, Ashford Robert L, Evans A: **Non-surgical interventions for paediatric pes planus.** In *Cochrane Database Syst Rev*: John Wiley & Sons, Ltd; 2010.
118. Roqué i Figuls M, Giné-Garriga M, Granados Rugeles C, Perrotta C: **Chest physiotherapy for acute bronchiolitis in paediatric patients between 0 and 24 months old.** In *Cochrane Database Syst Rev*: John Wiley & Sons, Ltd; 2012.
119. Rose Kristy J, Burns J, Wheeler Danielle M, North Kathryn N: **Interventions for increasing ankle range of motion in patients with neuromuscular disease.** In *Cochrane Database Syst Rev*: John Wiley & Sons, Ltd; 2010.
120. Russell EG: **Process versus outcome: challenges of the chiropractic wellness paradigm.** *J Chiropr Humanit* 2009, **16:**50-53.
121. Sansonnens N, Kunzler F, Bron C, Vassant M, Allet L: **The McKenzie method: Is this method efficient in short and long term for chronic non-specific low back pain? A systematic review.** *Kinesitherapie Revue* 2013, **13:**30-37.
122. Scheiman M, Gwiazda J, Li T: **Non-surgical interventions for convergence insufficiency.** In *Cochrane Database Syst Rev*: John Wiley & Sons, Ltd; 2011.
123. Schmid A, Brunner F, Wright A, Bachmann LM: **Paradigm shift in manual therapy: evidence for a central nervous system component in the response to passive cervical joint mobilisation.** *Man Ther* 2008, **13:**387-396.
124. Slade SC, Keating JL: **Unloaded movement facilitation exercise compared to no exercise or alternative therapy on outcomes for people with nonspecific chronic low back pain: a systematic review.** *J Manipulative Physiol Ther* 2007, **30:**301-311.
125. Slaven EJ, Goode AP, Coronado RA, Poole C, Hegedus EJ: **The relative effectiveness of segment specific level and non-specific level spinal joint mobilization on pain and range of motion: Results of a systematic review and meta-analysis.** *J Man Manip Ther* 2013, **21:**7-17.
126. Smith Caroline A, Collins Carmel T, Cyna Allan M, Crowther Caroline A: **Complementary and alternative therapies for pain management in labour.** In *Cochrane Database Syst Rev*: John Wiley & Sons, Ltd; 2006.
127. Smith Caroline A, Levett Kate M, Collins Carmel T, Jones L: **Massage, reflexology and other manual methods for pain management in labour.** In *Cochrane Database Syst Rev*: John Wiley & Sons, Ltd; 2012.
128. Snaith A, Wade D: **Dystonia.** *BMJ Clin Evid* 2008, **2008**.
129. Snaith A, Wade D: **Dystonia.** *BMJ Clin Evid* 2011, **2011**.
130. Solly SL: **Cervical postero-anterior mobilisation: a brief review of evidence of physiological and pain relieving effects.** *Phys Ther Rev* 2004, **9:**183-187.
131. Takasaki H, May S: **Mechanical diagnosis and therapy has similar effects on pain and disability as 'wait and see' and other approaches in people with neck pain: a systematic review (Provisional abstract).** In *Database of Abstracts of Reviews of Effects*. pp. 78-84; 2014:78-84.
132. Tan Kelvin H, Sabapathy A, Wei X: **Fetal manipulation for facilitating tests of fetal wellbeing.** In *Cochrane Database Syst Rev*: John Wiley & Sons, Ltd; 2013.

133. Teixeira Lázaro J, Valbuza Juliana S, Prado Gilmar F: **Physical therapy for Bell's palsy (idiopathic facial paralysis).** In *Cochrane Database Syst Rev*: John Wiley & Sons, Ltd; 2011.
134. Theadom A, Cropley M, Smith Helen E, Feigin Valery L, McPherson K: **Mind and body therapy for fibromyalgia.** In *Cochrane Database Syst Rev*: John Wiley & Sons, Ltd; 2015.
135. Tomlinson Claire L, Herd Clare P, Clarke Carl E, Meek C, Patel S, Stowe R, Deane Katherine HO, Shah L, Sackley Catherine M, Wheatley K, Ives N: **Physiotherapy for Parkinson's disease: a comparison of techniques.** In *Cochrane Database Syst Rev*: John Wiley & Sons, Ltd; 2014.
136. Tomlinson Claire L, Patel S, Meek C, Herd Clare P, Clarke Carl E, Stowe R, Shah L, Sackley Catherine M, Deane Katherine HO, Wheatley K, Ives N: **Physiotherapy versus placebo or no intervention in Parkinson's disease.** In *Cochrane Database Syst Rev*: John Wiley & Sons, Ltd; 2013.
137. Trescot AM, Chopra P, Abdi S, Datta S, Schultz DM: **Systematic review of effectiveness and complications of adhesiolysis in the management of chronic spinal pain: an update.** *Pain Physician* 2007, **10:**129-146.
138. Triano JJ: **Literature syntheses for the Council on Chiropractic Guidelines and Practice Parameters: methodology.** *J Manipulative Physiol Ther* 2008, **31:**645-650.
139. Triano JJ, Budgell B, Bagnulo A, Roffey B, Bergmann T, Cooperstein R, Gleberzon B, Good C, Perron J, Tepe R: **Review of methods used by chiropractors to determine the site for applying manipulation.** *Chiropr Man Therap* 2013, **21:**36.
140. Uden H, Boesch E, Kumar S: **Plantar fasciitis ? to jab or to support? A systematic review of the current best evidence.** *J Multidiscip Healthc* 2011, **4:**155-164.
141. Urrútia G, Burton AK, Morral Fernández A, Bonfill Cosp X, Zanoli G: **Neuroreflexotherapy for non-specific low-back pain.** In *Cochrane Database Syst Rev*: John Wiley & Sons, Ltd; 2004.
142. van der Velde G, Yu H, Paulden M, Cote P, Varatharajan S, Shearer HM, Wong JJ, Randhawa K, Southerst D, Mior S, et al: **Which interventions are cost-effective for the management of whiplash-associated and neck pain-associated disorders? a systematic review of the health economic literature by the ontario protocol for traffic injury management (optima) collaboration.** *Spine J* 2015.
143. van Ochten JM, van Middelkoop M, Meuffels D, Bierma-Zeinstra SM: **Chronic complaints after ankle sprains: a systematic review on effectiveness of treatments.** *J Orthop Sports Phys Ther* 2014, **44:**862-871, C861-823.
144. Velde GM: **Benign paroxysmal positional vertigo - part ii: a qualitative review of non-pharmacological, conservative treatments and a case report presenting Epley's "canalith repositioning procedure", a non-invasive bedside manoeuvre for treating BPPV.** *J Am Osteopath Assoc* 1999, **43:**41-49.
145. Verhagen Arianne P, Scholten-Peeters Gwendolijne GGM, van Wijngaarden S, de Bie R, Bierma-Zeinstra Sita MA: **Conservative treatments for whiplash.** In *Cochrane Database Syst Rev*: John Wiley & Sons, Ltd; 2007.
146. Vernon H, Humphreys BK, Hagino C: **The outcome of control groups in clinical trials of conservative treatments for chronic mechanical neck pain: a systematic review.** *BMC Musculoskelet Disord* 2006, **7:**58.
147. Wang MY, Tsai PS, Lee PH, Chang WY, Yang CM: **Systematic review and meta-analysis of the efficacy of tuina for cervical spondylosis.** *J Clin Nurs* 2008, **17:**2531-2538.
148. Wegner I, Widyahening Indah S, van Tulder Maurits W, Blomberg Stefan EI, de Vet Henrica CW, Brønfort G, Bouter Lex M, van der Heijden Geert J: **Traction for low-back pain with or without sciatica.** In *Cochrane Database Syst Rev*: John Wiley & Sons, Ltd; 2013.
149. West C, Hesketh A, Vail A, Bowen A: **Interventions for apraxia of speech following stroke.** In *Cochrane Database Syst Rev*: John Wiley & Sons, Ltd; 2005.
150. Winter J, Hunter S, Sim J, Crome P: **Hands-on therapy interventions for upper limb motor dysfunction following stroke.** In *Cochrane Database Syst Rev*: John Wiley & Sons, Ltd; 2011.

151. Wong CK, Abraham T, Karimi P, Ow-Wing C: **Strain counterstrain technique to decrease tender point palpation pain compared to control conditions: a systematic review with meta-analysis.** *J Bodyw Mov Ther* 2014, **18:**165-173.
152. Xia QC, Feng ZX, Ping CX: **Evaluating the efficacy of Tui Na in treatment of childhood anorexia: a meta-analysis.** *Altern Ther Health Med* 2014, **20:**45-52.
153. Yu H, Cote P, Shearer HM, Wong JJ, Sutton DA, Randhawa KA, Varatharajan S, Southerst D, Mior SA, Ameis A, et al: **Effectiveness of passive physical modalities for shoulder pain: systematic review by the Ontario protocol for traffic injury management collaboration.** *Phys Ther* 2015, **95:**306-318.
154. Yu H, Cote P, Southerst D, Wong JJ, Varatharajan S, Shearer HM, Gross DP, van der Velde GM, Carroll LJ, Mior SA, et al: **Does structured patient education improve the recovery and clinical outcomes of patients with neck pain? A systematic review from the Ontario Protocol for Traffic Injury Management (OPTIMa) Collaboration.** *Spine J* 2014.
155. Zaugg B, Wangler M: **A model framework for patient safety training in chiropractic: a literature synthesis.** *J Manipulative Physiol Ther* 2009, **32:**493-499.
156. Zhang Q, Sun Z, Yue J: **Massage therapy for preventing pressure ulcers.** In *Cochrane Database Syst Rev*: John Wiley & Sons, Ltd; 2015.
157. Zhang Y, Wang C, Guo Y: **[Of clinical reseach on cervical spondylosis treated with acupoint injection in recent five years].** *Zhongguo Zhen Jiu* 2012, **32:**477-479.
158. Zinck ND, Downie JW: **Plasticity in the injured spinal cord: can we use it to advantage to reestablish effective bladder voiding and continence?** *Prog Brain Res* 2006, **152:**147-162.
159. Aas Randi W, Tuntland H, Holte Kari A, Røe C, Lund T, Marklund S, Moller A: **Workplace interventions for neck pain in workers.** In *Cochrane Database Syst Rev*: John Wiley & Sons, Ltd; 2011.

From updated search:

1. Brown CK, Southerst D, Cote P, Shearer HM, Randhawa K, Wong JJ, Yu H, Varatharajan S, Sutton D, Stern PJ, et al: **The Effectiveness of Exercise on Recovery and Clinical Outcomes in Patients With Soft Tissue Injuries of the Hip, Thigh, or Knee: A Systematic Review by the Ontario Protocol for Traffic Injury Management (OPTIMa) Collaboration.** *J Manipulative Physiol Ther* 2016, **39:**110-120.e111.
2. Cheatham SW, Lee M, Cain M, Baker R: **The efficacy of instrument assisted soft tissue mobilization: a systematic review.** *J Can Chiropr Assoc* 2016, **60:**200-211.
3. Emtiazy M, Abrishamkar M: **The Effect of Massage Therapy on Children's Learning Process: A Review.** *Iran J Med Sci* 2016, **41:**S64.
4. Farber K, Wieland LS: **Massage for Low-back Pain.** *Explore (NY)* 2016, **12:**215-217.
5. Goldgrub R, Cote P, Sutton D, Wong JJ, Yu H, Randhawa K, Varatharajan S, Southerst D, Mior S, Shearer HM, et al: **The Effectiveness of Multimodal Care for the Management of Soft Tissue Injuries of the Shoulder: A Systematic Review by the Ontario Protocol for Traffic Injury Management (OPTIMa) Collaboration.** *J Manipulative Physiol Ther* 2016, **39:**121-139.e121.
6. Holmes MM, Lewith G, Newell D, Field J, Bishop FL: **The impact of patient-reported outcome measures in clinical practice for pain: a systematic review.** *Qual Life Res* 2016.
7. Macedo Luciana G, Saragiotto Bruno T, Yamato Tiê P, Costa Leonardo OP, Menezes Costa Luciola C, Ostelo Raymond WJG, Maher Christopher G: **Motor control exercise for acute non-specific low back pain.** In *Cochrane Database Syst Rev*: John Wiley & Sons, Ltd; 2016.
8. Mu PF, Chen YC, Cheng SC: **The effectiveness of non-pharmacological pain management in relieving chronic pain for children and adolescents.** *JBI Libr Syst Rev* 2009, **7:**1489-1543.
9. Poquet N, Lin Chung-Wei C, Heymans Martijn W, van Tulder Maurits W, Esmail R, Koes Bart W, Maher Christopher G: **Back schools for acute and subacute non-specific low-back pain.** In *Cochrane Database Syst Rev*: John Wiley & Sons, Ltd; 2016.

10. Randhawa K, Cote P, Gross DP, Wong JJ, Yu H, Sutton D, Southerst D, Varatharajan S, Mior S, Stupar M, et al: **The effectiveness of structured patient education for the management of musculoskeletal disorders and injuries of the extremities: a systematic review by the Ontario Protocol for Traffic Injury Management (OPTIMa) Collaboration.** *J Can Chiropr Assoc* 2015, **59:**349-362.
11. Roqué i Figuls M, Giné-Garriga M, Granados Rugeles C, Perrotta C, Vilaró J: **Chest physiotherapy for acute bronchiolitis in paediatric patients between 0 and 24 months old.** In *Cochrane Database Syst Rev*: John Wiley & Sons, Ltd; 2016.
12. Shin E-S, Seo K-H, Lee S-H, Jang J-E, Jung Y-M, Kim M-J, Yeon J-Y: **Massage with or without aromatherapy for symptom relief in people with cancer.** In *Cochrane Database Syst Rev*: John Wiley & Sons, Ltd; 2016.
13. Smart Keith M, Wand Benedict M, O'Connell Neil E: **Physiotherapy for pain and disability in adults with complex regional pain syndrome (CRPS) types I and II.** In *Cochrane Database Syst Rev*: John Wiley & Sons, Ltd; 2016.
14. Sutton D, Gross DP, Cote P, Randhawa K, Yu H, Wong JJ, Stern P, Varatharajan S, Southerst D, Shearer HM, et al: **Multimodal care for the management of musculoskeletal disorders of the elbow, forearm, wrist and hand: a systematic review by the Ontario Protocol for Traffic Injury Management (OPTIMa) Collaboration.** *Chiropr Man Therap* 2016, **24:**8.
15. Zaina F, Tomkins-Lane C, Carragee E, Negrini S: **Surgical versus non-surgical treatment for lumbar spinal stenosis.** In *Cochrane Database Syst Rev*: John Wiley & Sons, Ltd; 2016.

Not clear if SMT was included (27 records and 4 records*)

1. Baranowsky J, Klose P, Musial F, Hauser W, Dobos G, Langhorst J: **Qualitative systemic review of randomized controlled trials on complementary and alternative medicine treatments in fibromyalgia.** *Rheumatol Int* 2009, **30:**1-21.
2. Boldt I, Eriks-Hoogland I, Brinkhof Martin WG, de Bie R, Joggi D, von Elm E: **Non-pharmacological interventions for chronic pain in people with spinal cord injury.** In *Cochrane Database Syst Rev*: John Wiley & Sons, Ltd; 2014.
3. Camarinos J, Marinko L: **Effectiveness of manual physical therapy for painful shoulder conditions: a systematic review.** *J Man Manip Ther* 2009, **17:**206-215.
4. Chen X, Wang Z, Liang Y: **Effectiveness of non-surgical treatment of lumbar disc herniation: A systematic review. [Chinese].** *Chinese Journal of Evidence-Based Medicine* 2012, **12:**861-866.
5. Darlow B, Fullen BM, Dean S, Hurley DA, Baxter GD, Dowell A: **The association between health care professional attitudes and beliefs and the attitudes and beliefs, clinical management, and outcomes of patients with low back pain: a systematic review.** *Eur J Pain* 2012, **16:**3-17.
6. Feuerstein M, Burrell LM, Miller VI, Lincoln A, Huang GD, Berger R: **Clinical management of carpal tunnel syndrome: a 12-year review of outcomes.** *Am J Ind Med* 1999, **35:**232-245.
7. Goodyear-Smith F, Arroll B: **What can family physicians offer patients with carpal tunnel syndrome other than surgery? A systematic review of nonsurgical management.** *Ann Fam Med* 2004, **2:**267-273.
8. Handoll Helen HG, Elliott J: **Rehabilitation for distal radial fractures in adults.** In *Cochrane Database Syst Rev*: John Wiley & Sons, Ltd; 2015.
9. Handoll Helen HG, Sherrington C, Mak Jenson CS: **Interventions for improving mobility after hip fracture surgery in adults.** In *Cochrane Database Syst Rev*: John Wiley & Sons, Ltd; 2011.
10. Heijden GJ, Windt DA, Winter AF: **Physiotherapy for patients with soft tissue shoulder disorders: a systematic review of randomised clinical trials.** *BMJ* 1997, **315:**25-30.

11. Hollenbach D, Broker R, Herlehy S, Stuber K: **Non-pharmacological interventions for sleep quality and insomnia during pregnancy: A systematic review.** *J Can Chiropr Assoc* 2013, **57:**260-270.
12. Kamper Steven J, Apeldoorn Andreas T, Chiarotto A, Smeets Rob JEM, Ostelo Raymond WJG, Guzman J, van Tulder Maurits W: **Multidisciplinary biopsychosocial rehabilitation for chronic low back pain.** In *Cochrane Database Syst Rev*: John Wiley & Sons, Ltd; 2014.
13. Karjalainen Kaija A, Malmivaara A, van Tulder Maurits W, Roine R, Jauhiainen M, Hurri H, Koes Bart W: **Multidisciplinary biopsychosocial rehabilitation for neck and shoulder pain among working age adults.** In *Cochrane Database Syst Rev*: John Wiley & Sons, Ltd; 2003.
14. Khan F, Amatya B, Ng L, Drummond K, Galea M: **Multidisciplinary rehabilitation after primary brain tumour treatment.** In *Cochrane Database Syst Rev*: John Wiley & Sons, Ltd; 2015.
15. las Penas CF, Sohrbeck Campo M, Fernandez Carnero J, Miangolarra Page JC: **Manual therapies in myofascial trigger point treatment: a systematic review.** *J Bodyw Mov Ther* 2005, **9:**27-34.
16. Lauche R, Cramer H, Hauser W, Dobos G, Langhorst J: **A Systematic Overview of Reviews for Complementary and Alternative Therapies in the Treatment of the Fibromyalgia Syndrome.** *Evid Based Complement Alternat Med* 2015, **2015**.
17. Liao IC, Chen SL, Wang MY, Tsai PS: **Effects of Massage on Blood Pressure in Patients With Hypertension and Prehypertension: A Meta-analysis of Randomized Controlled Trials.** *J Cardiovasc Nurs* 2016, **31:**73-83.
18. n.a.: **Nonhormonal management of menopause-associated vasomotor symptoms: 2015 position statement of The North American Menopause Society.** *Menopause* 2015, **22:**1155-1174.
19. O'Connor D, Marshall Shawn C, Massy-Westropp N, Pitt V: **Non-surgical treatment (other than steroid injection) for carpal tunnel syndrome.** In *Cochrane Database Syst Rev*: John Wiley & Sons, Ltd; 2003.
20. Piazzini DB, Aprile I, Ferrara PE, Bertolini C, Tonali P, Maggi L, Rabini A, Piantelli S, Padua L: **A systematic review of conservative treatment of carpal tunnel syndrome.** *Clin Rehabil* 2007, **21:**299-314.
21. Poder TG, Lemieux R: **How effective are spiritual care and body manipulation therapies in pediatric oncology? A systematic review of the literature.** *Glob J Health Sci* 2014, **6:**112-127.
22. Pollock A, Baer G, Campbell P, Choo Pei L, Forster A, Morris J, Pomeroy Valerie M, Langhorne P: **Physical rehabilitation approaches for the recovery of function and mobility following stroke.** In *Cochrane Database Syst Rev*: John Wiley & Sons, Ltd; 2014.
23. Scheer SJ, Radack KL, O'Brien DR: **Randomized controlled trials (RCTs) in industrial low back pain relating to return to work - Part 2: discogenic low back pain.** *Arch Phys Med Rehabil* 1996, **77:**1189-1197.
24. Stevinson C, Ernst E: **Complementary/alternative therapies for premenstrual syndrome: a systematic review of randomized controlled trials.** *Am J Obstet Gynecol* 2001, **185:**227-235.
25. Turner-Stokes L, Nair A, Sedki I, Disler Peter B, Wade Derick T: **Multi-disciplinary rehabilitation for acquired brain injury in adults of working age.** In *Cochrane Database Syst Rev*: John Wiley & Sons, Ltd; 2005.
26. Wilkinson S, Barnes K, Storey L: **Massage for symptom relief in patients with cancer: systematic review.** *J Adv Nurs* 2008, **63:**430-439.
27. Winkelmann A, Hauser W, Friedel E, Moog-Egan M, Seeger D, Settan M, Weiss T, Schiltenwolf M: **[Physiotherapy and physical therapies for fibromyalgia syndrome. Systematic review, meta-analysis and guideline].** *Schmerz* 2012, **26:**276-286.

From updated search:

1. Hejazi ZA, Namjooyan F, Khanifar M: **Complementary and Alternative Medicine for Osteoporosis.** *Iran J Med Sci* 2016, **41:**S27.
2. Sutton DA, Nordin M, Cote P, Randhawa K, Yu H, Wong JJ, Stern P, Varatharajan S, Southerst D, Shearer HM, et al: **The Effectiveness of Multimodal Care for Soft Tissue Injuries of the Lower**

Extremity: A Systematic Review by the Ontario Protocol for Traffic Injury Management (OPTIMa) Collaboration. *J Manipulative Physiol Ther* 2016, **39:**95-109.e101-102.
3. Turner-Stokes L, Pick A, Nair A, Disler Peter B, Wade Derick T: **Multi-disciplinary rehabilitation for acquired brain injury in adults of working age.** In *Cochrane Database Syst Rev*: John Wiley & Sons, Ltd; 2015.
4. Yang L, Bian Y, Shao J, Sheng W, Li W, Zeng L: **Efficacy and safety of chiropractic therapy in infantile anorexia: A systematic review.** *Eur J Integr Med* 2016, **8:**106-112.

Primary research (31 records and 1 record*)

1. Aickin M, McCaffery A, Pugh G, Tick H, Ritenbaugh C, Hicks P, Pelletier KR, Cao J, Himick D, Monahan J: **Description of a clinical stream of back-pain patients based on electronic medical records.** *Complement Ther Clin Pract* 2013, **19:**158-176.
2. Apeldoorn AT, Ostelo RW, Van Helvoirt H, Fritz JM, De Vet HCW, Van Tulder MW: **The cost-effectiveness of a treatment-based classification system for low back pain: Design of a randomised controlled trial and economic evaluation.** *BMC Musculoskelet Disord* 2010, **11**.
3. Bien JY, Morel J, Demasles S, Abboud K, Molliex S: **[Postoperative dissection of the vertebral artery in two steps].** *Ann Fr Anesth Reanim* 2014, **33:**696-699.
4. Cerritelli F, Martelli M, Renzetti C, Pizzolorusso G, Cozzolino V, Barlafante G: **Introducing an osteopathic approach into neonatology ward: The NE-O model.** *Chiropr Man Therap* 2014, **22**.
5. Chiu TW, Wright A: **To compare the effects of different rates of application of a cervical mobilisation technique on sympathetic outflow to the upper limb in normal subjects.** *Man Ther* 1996, **1:**198-203.
6. Cramer GD, Ross K, Pocius J, Cantu JA, Laptook E, Fergus M, Gregerson D, Selby S, Raju PK: **Evaluating the relationship among cavitation, zygapophyseal joint gapping, and spinal manipulation: an exploratory case series.** *J Manipulative Physiol Ther* 2011, **34:**2-14.
7. Daffner SD, Hymanson HJ, Wang JC: **Cost and use of conservative management of lumbar disc herniation before surgical discectomy.** *Spine J* 2010, **10:**463-468.
8. DeBar LL, Elder C, Ritenbaugh C, Aickin M, Deyo R, Meenan R, Dickerson J, Webster JA, Jo Yarborough B: **Acupuncture and chiropractic care for chronic pain in an integrated health plan: a mixed methods study.** *BMC Complement Altern Med* 2011, **11**.
9. Field J, Newell D: **Relationship between STarT Back Screening Tool and prognosis for low back pain patients receiving spinal manipulative therapy.** *Chiropr Man Therap* 2012, **20**.
10. Gemmell H, Miller P: **Relative effectiveness and adverse effects of cervical manipulation, mobilisation and the activator instrument in patients with sub-acute non-specific neck pain: Results from a stopped randomised trial.** *Chiropr Osteopat* 2010, **18**.
11. Hestbaek L, Kongsted A, Jensen T, Leboeuf-Yde C: **The clinical aspects of the acute facet syndrome: Results from a structured discussion among European chiropractors.** *Chiropr Osteopat* 2009, **17**.
12. Jones J, Jones C, Nugent K: **Vertebral artery dissection after a chiropractor neck manipulation.** *Proc (Bayl Univ Med Cent)* 2015, **28:**88-90.
13. Julian C, Hoskins W, Vitiello AL: **Sports chiropractic management at the World Ice Hockey Championships.** *Chiropr Osteopat* 2010, **18**.
14. Konrad K, Gerencser F: **Manual treatment in patients with vertigo. [German].** *Manuelle Medizin* 1990, **28:**62-64.
15. Leach RA: **Patients with symptoms and signs of stroke presenting to a rural chiropractic practice.** *J Manipulative Physiol Ther* 2010, **33:**62-69.
16. Leboeuf-Yde C, Rosenbaum A, Axen I, Lovgren PW, Jorgensen K, Halasz L, Eklund A, Wedderkopp N: **The Nordic Subpopulation Research Programme: Prediction of treatment outcome in patients**

with low back pain treated by chiropractors - does the psychological profile matter? *Chiropr Osteopat* 2009, **17**.
17. Ledonio CG, Polly DW, Jr., Swiontkowski MF, Cummings JT, Jr.: **Comparative effectiveness of open versus minimally invasive sacroiliac joint fusion.** *Med Devices (Auckl)* 2014, **7:**187-193.
18. Maiers MJ, Hartvigsen J, Schulz C, Schulz K, Evans RL, Bronfort G: **Chiropractic and exercise for seniors with low back pain or neck pain: The design of two randomized clinical trials.** *BMC Musculoskelet Disord* 2007, **8**.
19. Malmqvist S, Leboeuf-Yde C: **The Nordic maintenance care program: Case management of chiropractic patients with low back pain - Defining the patients suitable for various management strategies.** *Chiropr Osteopat* 2009, **17**.
20. Marchand AM, Miller JE, Mitchell C: **Diagnosis and chiropractic treatment of infant headache based on behavioral presentation and physical findings: a retrospective series of 13 cases.** *J Manipulative Physiol Ther* 2009, **32:**682-686.
21. Maruyama Y, Shimoji K, Shimizu H: **Human spinal cord potentials evoked by different sources of stimulation and conduction velocities along the cord.** *J Neurophysiol* 1982, **48:**1098-1107.
22. Newell D, Field J, Visnes N: **Prognostic accuracy of clinicians for back, neck and shoulder patients in routine practice.** *Chiropr Man Therap* 2013, **21**.
23. Pelletier KR, Astin JA, Haskell WL: **Current trends in the integration and reimbursement of complementary and alternative medicine by managed care organizations (MCOs) and insurance providers: 1998 update and cohort analysis.** *Am J Health Promot* 1999, **14:**125-133.
24. Petersen N, Vicenzino B, Wright A: **The effects of a cervical mobilisation technique on sympathetic outflow to the upper limb in normal subjects.** *Physiother Theory Pract* 1993, **9:**149-156.
25. Polkinghorn BS, Colloca CJ: **Treatment of symptomatic lumbar disc herniation using activator methods chiropractic technique.** *J Manipulative Physiol Ther* 1998, **21:**187-196.
26. Rajaii RM, Cox GJ, Schneider RP: **Role of osteopathic manipulative treatment in the management of stiff person syndrome.** *J Am Osteopath Assoc* 2015, **115:**394-398.
27. Sandnes KF, Bjornstad C, Leboeuf-Yde C, Hestbaek L: **The Nordic Maintenance Care Program - Time intervals between treatments of patients with low back pain: How close and who decides?** *Chiropr Osteopat* 2010, **18**.
28. Shvartzman P, Abelson A: **Complications of chiropractic treatment for back pain.** *Postgrad Med* 1988, **83:**57-58, 61.
29. Slater RN, Spencer JD: **Central lumbar disc prolapse following chiropractic manipulation: a call for audit of 'alternative practice'.** *J R Soc Med* 1992, **85:**637-638.
30. Spallek M, Kuhn W, Uibel S, Van Mark A, Quarcoo D: **Work-related musculoskeletal disorders in the automotive industry due to repetitive work - Implications for rehabilitation.** *J Occup Med Toxicol* 2010, **5**.
31. Young YH, Chen CH: **Acute vertigo following cervical manipulation.** *Laryngoscope* 2003, **113:**659-662.

From updated search:

1. Yang HS, Oh YM, Eun JP: **Cervical Intradural Disc Herniation Causing Progressive Quadriparesis after Spinal Manipulation Therapy.** *Medicine (Baltimore)* 2016, **95 (6) (no pagination)**.

Non-systematic (301 records and 13 records*)

1. Adams AH, Gatterman M: **The state of the art of research on chiropractic education.** *J Manipulative Physiol Ther* 1997, **20:**179-184.

2. Alcantara J, Alcantara JD, Alcantara J: **The chiropractic care of infants with colic: a systematic review of the literature.** *Explore (NY)* 2011, **7:**168-174.
3. Alcantara J, Alcantara JD, Alcantara J: **A systematic review of the literature on the chiropractic care of patients with autism spectrum disorder.** *Explore (NY)* 2011, **7:**384-390.
4. Alcantara J, Alcantara JD, Alcantara J: **The chiropractic care of patients with cancer: a systematic review of the literature.** *Integr Cancer Ther* 2012, **11:**304-312.
5. Alcantara J, Alcantara JD, Alcantara J: **The Chiropractic Care of Infants with Breastfeeding Difficulties.** *Explore (NY)* 2015, **11:**468-474.
6. Alcantara J, Alcantara JD, Alcantara J: **The use of validated outcome measures in the chiropractic care of pregnant patients: A systematic review of the literature.** *Complement Ther Clin Pract* 2015, **21:**131-136.
7. Alix ME, Bates DK: **A proposed etiology of cervicogenic headache: the neurophysiologic basis and anatomic relationship between the dura mater and the rectus posterior capitis minor muscle.** *J Manipulative Physiol Ther* 1999, **22:**534-539.
8. An GH, Zhao Y, Sun P: **Mechanism and application of Jiaji point in the treatment of cervical spondylosis. [Chinese].** *Chinese Journal of Clinical Rehabilitation* 2006, **10:**129-131.
9. Anderson-Peacock E, Blouin JS, Bryans R, Danis N, Furlan A, Marcoux H, Potter B, Ruegg R, Stein JG, White E: **Chiropractic clinical practice guideline: evidence-based treatment of adult neck pain not due to whiplash.** *J Can Chiropr Assoc* 2005, **49:**158-209.
10. Angus K, Asgharifar S, Gleberzon B: **What effect does chiropractic treatment have on gastrointestinal (GI) disorders: a narrative review of the literature.** *J Can Chiropr Assoc* 2015, **59:**122-133.
11. Aronsson DD, Loder RT: **Treatment of the unstable (acute) slipped capital femoral epiphysis.** *Clin Orthop Relat Res* 1996**:**99-110.
12. Assendelft WJ, Bouter LM: **Does the goose really lay golden eggs? A methodological review of Workmen's Compensation studies.** *J Manipulative Physiol Ther* 1993, **16:**161-168.
13. Assendelft WJ, Koes BW, Knipschild PG, Bouter LM: **The relationship between methodological quality and conclusions in reviews of spinal manipulation.** *JAMA* 1995, **274:**1942-1948.
14. Assendelft WJ, Koes BW, van der Heijden GJ, Bouter LM: **The efficacy of chiropractic manipulation for back pain: blinded review of relevant randomized clinical trials.** *J Manipulative Physiol Ther* 1992, **15:**487-494.
15. Assendelft WJ, Lankhorst GJ: **[Effectiveness of manipulative therapy in low back pain: systematic literature reviews and guidelines are inconclusive].** *Ned Tijdschr Geneeskd* 1998, **142:**684-687.
16. Bailey HW: **Some problems in making osteopathic spinal manipulative therapy appropriate and specific.** *J Am Osteopath Assoc* 1976, **75:**486-499.
17. Baldwin ML, Cote P, Frank JW, Johnson WG: **Cost-effectiveness studies of medical and chiropractic care for occupational low back pain. a critical review of the literature.** *Spine J* 2001, **1:**138-147.
18. Balon JW, Mior SA: **Chiropractic care in asthma and allergy.** *Ann Allergy Asthma Immunol* 2004, **93:**S55-60.
19. Bayer K: **Vertebral artery dissection and chirotherapy. [German].** *Manuelle Medizin* 1998, **36:**241-245.
20. Bayme MJ, Geftler A, Netz U, Kirshtein B, Glazer Y, Atias S, Perry Z: **The perils of complementary alternative medicine.** *Rambam Maimonides Med J* 2014, **5**.
21. Bendix T: **[Spinal manipulation for chronic low back pain--a survey of a chronic review].** *Ugeskr Laeger* 2011, **173:**2790-2793.
22. Bergman GJ, Winter JC, Van Tulder MW, Meyboom-De Jong B, Postema K, Van Der Heijden GJ: **Manipulative therapy in addition to usual medical care accelerates recovery of shoulder complaints at higher costs: Economic outcomes of a randomized trial.** *BMC Musculoskelet Disord* 2010, **11**.
23. Bergmann TF: **Short lever, specific contact articular chiropractic technique.** *J Manipulative Physiol Ther* 1992, **15:**591-595.

24. Bergmann TF: **Manual force, mechanically assisted articular chiropractic technique using long and/or short level contacts.** *J Manipulative Physiol Ther* 1993, **16:**33-36.
25. Berman BM, Swyers JP: **Establishing a research agenda for investigating alternative medical interventions for chronic pain.** *Prim Care* 1997, **24:**743-758.
26. Binder AI: **Neck pain.** *BMJ Clin Evid* 2008, **2008**.
27. Biondi DM: **Physical treatments for headache: a structured review.** *Headache* 2005, **45:**738-746.
28. Boal RW, Gillette RG: **Central neuronal plasticity, low back pain and spinal manipulative therapy.** *J Manipulative Physiol Ther* 2004, **27:**314-326.
29. Bolton PS, Budgell B: **Visceral responses to spinal manipulation.** *J Electromyogr Kinesiol* 2012, **22:**777-784.
30. Borge JA, Leboeuf-Yde C, Lothe J: **Erratum: Prognostic values of physical examination findings in patients with chronic low back pain treated conservatively: A systematic literature review (Journal of Manipulative and Physiological Therapeutics (2001) 24 (292-295)).** *J Manipulative Physiol Ther* 2001, **24:**377.
31. Borggren CL: **Pregnancy and chiropractic: a narrative review of the literature.** *J Chiropr Med* 2007, **6:**70-74.
32. Boucher P, Robidoux S: **Lumbar disc herniation and cauda equina syndrome following spinal manipulative therapy: a review of six court decisions in Canada.** *J Forensic Leg Med* 2014, **22:**159-169.
33. Brand PLP, Engelbert RHH, Helders PJM, Offringa M: **Systematic review of effects of manual therapy in infants with kinetic imbalance due to suboccipital strain (KISS) syndrome.** *J Man Manip Ther* 2005, **13:**209-214.
34. Braus DF, Mainka R: **Stroke following chiropractic manipulation: A rational strategy for diagnosis. [German].** *Manuelle Medizin* 1993, **31:**92-96.
35. Breen A, Vogel S, Pincus T, Foster N, Underwood M: **Systematic review of spinal manipulation: A balanced review of evidence?** *J R Soc Med* 2006, **99:**277; author reply 279-280.
36. Bronfort G, Haas M, Moher D, Bouter L, van Tulder M, Triano J, Assendelft WJ, Evans R, Dagenais S, Rosner A: **Review conclusions by Ernst and Canter regarding spinal manipulation refuted.** *Chiropr Osteopat* 2006, **14:**14.
37. Brown R: **A health care system in transformation: Making the case for chiropractic.** *Chiropr Man Therap* 2012, **20**.
38. Byfield D, McCarthy P: **Systematic review of spinal manipulation: Flaws in the review.** *J R Soc Med* 2006, **99:**277-278, author reply 279-280.
39. Canter PH, Coon JT, Ernst E: **Cost-effectiveness of complementary therapies in the United kingdom-a systematic review.** *Evid Based Complement Alternat Med* 2006, **3:**425-432.
40. Carlesso LC, Macdermid JC, Santaguida LP: **Standardization of adverse event terminology and reporting in orthopaedic physical therapy: application to the cervical spine.** *J Orthop Sports Phys Ther* 2010, **40:**455-463.
41. Carr RR, Nahata MC: **Complementary and alternative medicine for upper-respiratory-tract infection in children.** *Am J Health Syst Pharm* 2006, **63:**33-39.
42. Cates JR, Young DN, Guerriero DJ, Jahn WT, Armine JP, Korbett AB, Bowerman DS, Porter RC, Sandman TD, King RA: **Evaluating the quality of clinical practice guidelines.** *J Manipulative Physiol Ther* 2001, **24:**170-176.
43. CCOHTA: **Spinal manipulation for lower back pain (Structured abstract).** In *Health Technology Assessment Database*: Canadian Coordinating Office for Health Technology Assessment (CCOHTA); 2002.
44. Chaitow L, Comeaux Z, Dommerholt J, Ernst E, Gibbons P, Hannon J, Lewis D, Liebenson C: **Effectiveness of manipulation in the treatment of low back pain: Validity of the conclusions in metaanalyses. [German].** *Osteopathische Medizin* 2004, **5:**13-19.

45. Chaitow L, Comeaux Z, Dommerholt J, Ernst E, Gibbons P, Hannon J, Lewis D, Liebenson C: **Efficacy of manipulation in low back pain treatment: The validity of meta-analysis conclusions.** *J Bodyw Mov Ther* 2004, **8:**25-31.
46. Cherkin DC, Sherman KJ, Deyo RA, Shekelle PG: **A review of the evidence for the effectiveness, safety, and cost of acupuncture, massage therapy, and spinal manipulation for back pain.** *Ann Intern Med* 2003, **138:**898-906.
47. Chou R: **Low back pain (chronic).** *BMJ Clin Evid* 2010, **2010**.
48. Chung CL, Cote P, Stern P, L'Esperance G: **The Association Between Cervical Spine Manipulation and Carotid Artery Dissection: A Systematic Review of the Literature.** *J Manipulative Physiol Ther* 2014.
49. Cooper R, Stoflet S: **Diversity and consistency: the challenge of maintaining quality in a multidisciplinary workforce.** *J Health Serv Res Policy* 2004, **9 Suppl 1:**39-47.
50. Cooperstein R, Perle SM, Gatterman MI, Lantz C, Schneider MJ: **Chiropractic technique procedures for specific low back conditions: characterizing the literature.** *J Manipulative Physiol Ther* 2001, **24:**407-424.
51. Coulter I: **Manipulation and mobilization of the cervical spine: The results of a literature survey and consensus panel.** *J Musculoskelet Pain* 1996, **4:**113-123.
52. Coulter ID: **Efficacy and risks of chiropractic manipulation: What does the evidence suggest?** *Integrative Medicine* 1998, **1:**61-66.
53. Cramer G, Budgell B, Henderson C, Khalsa P, Pickar J: **Basic science research related to chiropractic spinal adjusting: the state of the art and recommendations revisited.** *J Manipulative Physiol Ther* 2006, **29:**726-761.
54. Cravatari M, Schillan M, Ponte E: **Carotid dissection and cerebral infaction in young people. [Italian].** *Nuova Riv Neurol* 2003, **13:**126-130.
55. Croisile B, Aimard G, Vighetto A, Vial C, Confavreux C, Trillet M: **[Neck pain and isolated torticollis revealing neurologic lesions].** *Presse Med* 1989, **18:**1513-1515.
56. Crossley K, Bennell K, Green S, McConnell J: **A systematic review of physical interventions for patellofemoral pain syndrome.** *Clin J Sport Med* 2001, **11:**103-110.
57. Crotteau CA, Wright ST: **What is the best treatment for infants with colic?** *J Fam Pract* 2006, **55:**634-636.
58. da Paz AC, Carod Artal FJ, Kalil RK: **The function of proprioceptors in bone organization: a possible explanation for neurogenic heterotopic ossification in patients with neurological damage.** *Med Hypotheses* 2007, **68:**67-73.
59. Dagenais S, Gay RE, Tricco AC, Freeman MD, Mayer JM: **NASS Contemporary Concepts in Spine Care: spinal manipulation therapy for acute low back pain.** *Spine J* 2010, **10:**918-940.
60. Dagenais S, Mayer J, Wooley JR, Haldeman S: **Evidence-informed management of chronic low back pain with medicine-assisted manipulation.** *Spine J* 2008, **8:**142-149.
61. Dagenais S, Roffey DM, Wai EK, Haldeman S, Caro J: **Can cost utility evaluations inform decision making about interventions for low back pain?** *Spine J* 2009, **9:**944-957.
62. Dalby BJ: **Chiropractic diagnosis and treatment of closed head trauma.** *J Manipulative Physiol Ther* 1993, **16:**392-400.
63. D'Amico D, Grazzi L, Usai S, Leonardi M, Raggi A: **Disability and quality of life in headache: where we are now and where we are heading.** *Neurol Sci* 2013, **34 Suppl 1:**S1-5.
64. Davis C: **Chronic pain/dysfunction in whiplash-associated disorders.** *J Manipulative Physiol Ther* 2001, **24:**44-51.
65. Davis PT, Hulbert JR: **Carpal tunnel syndrome: conservative and nonconservative treatment. A chiropractic physician's perspective.** *J Manipulative Physiol Ther* 1998, **21:**356-362.
66. Di Duro JO: **Improvement in hearing after chiropractic care: a case series.** *Chiropr Osteopat* 2006, **14**.
67. Di Fabio RP: **Efficacy of manual therapy.** *Phys Ther* 1992, **72:**853-864.

68. DiGiorgi D: **Spinal manipulation under anesthesia: A narrative review of the literature and commentary.** *Chiropr Man Therap* 2013, **21**.
69. Dobkin BH: **Neurobiology of rehabilitation.** *Ann N Y Acad Sci* 2004, **1038:**148-170.
70. Dougherty PE, Hawk C, Weiner DK, Gleberzon B, Andrew K, Killinger L: **The role of chiropractic care in older adults.** *Chiropr Man Therap* 2012, **20**.
71. Downie AS, Vemulpad S, Bull PW: **Quantifying the high-velocity, low-amplitude spinal manipulative thrust: a systematic review.** *J Manipulative Physiol Ther* 2010, **33:**542-553.
72. Driscoll MD: **Arterial tonometry and assessment of cardiovascular alterations with chiropractic spinal manipulative therapy.** *J Manipulative Physiol Ther* 1997, **20:**47-55.
73. Ehrlich GE: **Back pain.** *J Rheumatol Suppl* 2003, **67:**26-31.
74. Eisenberg DM, Cohen MH, Hrbek A, Grayzel J, Van Rompay MI, Cooper RA: **Credentialing complementary and alternative medical providers.** *Ann Intern Med* 2002, **137:**965-973.
75. Engel RM, Vemulpad SR, Dougherty P: **Safety of thrust joint manipulation in the thoracic spine: A systematic review.** *J Man Manip Ther* 2015, **23:**173.
76. Ernst E: **The use, efficacy, safety and costs of complementary/alternative therapies for low back pain.** *European Journal of Physical Medicine and Rehabilitation* 1998, **8:**53-57.
77. Ernst E: **Complementary medicine: where is the evidence?** *J Fam Pract* 2003, **52:**630-634.
78. Ernst E, Canter P: **Erratum: Systematic review of spinal manipulation: Authors' reply (Journal of the Royal Society of Medicine (2006) 99 (279-280)).** *J R Soc Med* 2006, **99:**336.
79. Ernst E, Canter PH: **A systematic review of systematic reviews of spinal manipulation.** *J R Soc Med* 2006, **99:**192-196.
80. Ernst E, Posadzki P: **An independent review of NCCAM-funded studies of chiropractic.** *Clin Rheumatol* 2011, **30:**593-600.
81. Ernst E, Posadzki P: **Reporting of adverse effects in randomised clinical trials of chiropractic manipulations: a systematic review.** *N Z Med J* 2012, **125:**87-140.
82. Evans DW: **Mechanisms and effects of spinal high-velocity, low-amplitude thrust manipulation: previous theories.** *J Manipulative Physiol Ther* 2002, **25:**251-262.
83. Evans G, Richards S: **Low back pain: an evaluation of therapeutic interventions (Structured abstract).** In *Database of Abstracts of Reviews of Effects*. pp. 176: University of Bristol, Department of Social Medicine, Health Care Evaluation Unit; 1996:176.
84. Evans Jr MW, Rupert R: **The council on Chiropractic Education's new Wellness Standard: A call to action for the chiropractic profession.** *Chiropr Osteopat* 2006, **14**.
85. Everett CR, Patel RK: **A systematic literature review of nonsurgical treatment in adult scoliosis.** *Spine (Phila Pa 1976)* 2007, **32:**S130-134.
86. Farfan HF: **The scientific basis of manipulative procedures.** *Clin Rheum Dis* 1980, **6:**159-177.
87. Fenton BW: **Limbic associated pelvic pain: a hypothesis to explain the diagnostic relationships and features of patients with chronic pelvic pain.** *Med Hypotheses* 2007, **69:**282-286.
88. Fernandez-de-Las-Penas C, Courtney CA: **Clinical reasoning for manual therapy management of tension type and cervicogenic headache.** *J Man Manip Ther* 2014, **22:**44-50.
89. Ferrance RJ, Miller J: **Chiropractic diagnosis and management of non-musculoskeletal conditions in children and adolescents.** *Chiropr Osteopat* 2010, **18:**14.
90. Fishbain DA: **Non-surgical chronic pain treatment outcome: A review.** *Int Rev Psychiatry* 2000, **12:**170-180.
91. Fitz-Ritson D: **Therapeutic traction: a review of neurological principles and clinical applications.** *J Manipulative Physiol Ther* 1984, **7:**39-49.
92. Foreman RD, Qin C: **Neuromodulation of cardiac pain and cerebral vasculature: neural mechanisms.** *Cleve Clin J Med* 2009, **76 Suppl 2:**S75-79.
93. Fowler RP: **Recommendations for management of uncomplicated back pain in the workers' compensation system: a focus on functional restoration.** *J Chiropr Med* 2004, **3:**129-137.

94. Franke H: **Why reservations remain: a critical reflection about the systematic review and meta-analysis "Osteopathic manipulative treatment for low back pain" by Licciardone et al.** *J Bodyw Mov Ther* 2012, **16:**411-415.
95. French SD, Green SE: **The Cochrane Collaboration: is it relevant for doctors of chiropractic?** *J Manipulative Physiol Ther* 2005, **28:**641-642.
96. French SD, Walker BF, Perle SM: **Chiropractic care for children: Too much, too little or not enough?** *Chiropr Osteopat* 2010, **18**.
97. Fritz JM, Childs JD, Flynn TW: **Pragmatic application of a clinical prediction rule in primary care to identify patients with low back pain with a good prognosis following a brief spinal manipulation intervention.** *BMC Fam Pract* 2005, **6**.
98. Fryer G, Morris T, Gibbons P: **Paraspinal muscles and intervertebral dysfunction: part two.** *J Manipulative Physiol Ther* 2004, **27:**348-357.
99. Frymann VM: **Learning difficulties of children viewed in the light of the osteopathic concept.** *J Am Osteopath Assoc* 1976, **76:**46-61.
100. Gaines E, Chila AG: **Communication for osteopathic manipulative treatment (OMT): the language of lived experience in OMT pedagogy.** *J Am Osteopath Assoc* 1998, **98:**164-168.
101. Gamber R, Holland S, Russo DP, Cruser d A, Hilsenrath PE: **Cost-effective osteopathic manipulative medicine: a literature review of cost-effectiveness analyses for osteopathic manipulative treatment.** *J Am Osteopath Assoc* 2005, **105:**357-367.
102. Gay RE, Brault JS: **Evidence-informed management of chronic low back pain with traction therapy.** *Spine J* 2008, **8:**234-242.
103. Gay RE, Bronfort G, Evans RL: **Distraction manipulation of the lumbar spine: a review of the literature.** *J Manipulative Physiol Ther* 2005, **28:**266-273.
104. Gerencser F, Balint G, Konrad K, Scheidl Z: **Evaluation of the diagnostic and therapeutic experiences of the authors in cervical syndrome. [Hungarian].** *Rheumatol Balneol Allergol* 1978, **19:**65-69.
105. Gerow G, Matthews B, Jahn W, Gerow R: **Compartment syndrome and shin splints of the lower leg.** *J Manipulative Physiol Ther* 1993, **16:**245-252.
106. Gerstenbrand F, Kotscher E, Tischer H: **The upper cervical syndrome. [German].** *Z Orthop Ihre Grenzgeb* 1974, **112:**1249-1255.
107. Getzoff H: **Disc technique: An adjusting procedure for any lumbar discogenic syndrome.** *J Chiropr Med* 2003, **2:**142-144.
108. Gleberzon BJ: **A narrative review of the published chiropractic literature regarding older patients from 2001-2010.** *J Can Chiropr Assoc* 2011, **55:**76-95.
109. Goldenberg DL, Burchhardt C, Crofford L: **Useful treatments for fibromyalgia syndrome.** *J Fam Pract* 2005, **54:**105.
110. Gonzalez I: **Impact of manual osteopathic techniques on muscle proprioceptors: a literature review. [Spanish].** *Osteopatia Cientifica* 2009, **4:**70-75.
111. Gordin K, Hauser R: **The case for utilizing prolotherapy as a promising stand-alone or adjunctive treatment for over-manipulation syndrome.** *J Appl Res* 2013, **13:**1-28.
112. Gordon R, Cremata E, Hawk C: **Guidelines for the practice and performance of manipulation under anesthesia.** *Chiropr Man Therap* 2014, **22**.
113. Gotlib A, Rupert R: **Assessing the evidence for the use of chiropractic manipulation in paediatric health conditions: A systematic review.** *Paediatr Child Health* 2005, **10:**157-161.
114. Goto V, Frange C, Andersen ML, Junior JM, Tufik S, Hachul H: **Chiropractic intervention in the treatment of postmenopausal climacteric symptoms and insomnia: A review.** *Maturitas* 2014, **78:**3-7.
115. Gottlieb MS: **Conservative management of spinal osteoarthritis with glucosamine sulfate and chiropractic treatment.** *J Manipulative Physiol Ther* 1997, **20:**400-414.
116. Grace S: **CAM practitioners in the Australian health workforce: An underutilized resource.** *BMC Complement Altern Med* 2012, **12**.

117. Grandas OH, Klar M, Goldman MH, Filston HC: **Deep venous thrombosis in the pediatric trauma population: an unusual event: report of three cases.** *Am Surg* 2000, **66:**273-276.
118. Haldeman S: **Spinal manipulative therapy in sports medicine.** *Clin Sports Med* 1986, **5:**277-293.
119. Haldeman S, Dagenais S: **Cervicogenic headaches: a critical review.** *Spine J* 2001, **1:**31-46.
120. Hall H, McIntosh G: **Low back pain (acute).** *BMJ Clin Evid* 2008, **2008**.
121. Hall H, McIntosh G: **Low back pain (chronic).** *BMJ Clin Evid* 2008, **2008**.
122. Haneline M, Triano J: **Cervical artery dissection. A comparison of highly dynamic mechanisms: manipulation versus motor vehicle collision.** *J Manipulative Physiol Ther* 2005, **28:**57-63.
123. Haneline MT: **Chiropractic manipulation and acute neck pain: a review of the evidence.** *J Manipulative Physiol Ther* 2005, **28:**520-525.
124. Haneline MT: **Safety of chiropractic interventions: a systematic review.** *Spine (Phila Pa 1976)* 2009, **34:**2475-2476; author reply 2476-2477.
125. Haneline MT, Croft AC, Frishberg BM: **Association of internal carotid artery dissection and chiropractic manipulation.** *Neurologist* 2003, **9:**35-44.
126. Haneline MT, Lewkovich GN: **An analysis of the etiology of cervical artery dissections: 1994 to 2003.** *J Manipulative Physiol Ther* 2005, **28:**617-622.
127. Harrison DD, Colloca CJ, Troyanovich SJ, Harrison DE: **Torque: an appraisal of misuse of terminology in chiropractic literature and technique.** *J Manipulative Physiol Ther* 1996, **19:**454-462.
128. Harrison DE, Cailliet R, Harrison DD, Troyanovich SJ, Harrison SO: **A review of biomechanics of the central nervous system--Part III: spinal cord stresses from postural loads and their neurologic effects.** *J Manipulative Physiol Ther* 1999, **22:**399-410.
129. Haun DW, Kettner NW: **Spondylolysis and spondylolisthesis: a narrative review of etiology, diagnosis, and conservative management.** *J Chiropr Med* 2005, **4:**206-217.
130. Hawk C, Evans Jr MW: **A framework for chiropractic training in clinical preventive services.** *Chiropr Man Therap* 2013, **21**.
131. Hawk C, Schneider M, Ferrance RJ, Hewitt E, Van Loon M, Tanis L: **Best practices recommendations for chiropractic care for infants, children, and adolescents: results of a consensus process.** *J Manipulative Physiol Ther* 2009, **32:**639-647.
132. Hebert JJ, Koppenhaver SL, Walker BF: **Subgrouping patients with low back pain: a treatment-based approach to classification.** *Sports Health* 2011, **3:**534-542.
133. Hession EF, Donald GD: **Treatment of multiple lumbar disk herniations in an adolescent athlete utilizing flexion distraction and rotational manipulation.** *J Manipulative Physiol Ther* 1993, **16:**185-192.
134. Hestbaek L, Stochkendahl MJ: **The evidence base for chiropractic treatment of musculoskeletal conditions in children and adolescents: The emperor's new suit?** *Chiropr Osteopat* 2010, **18:**15.
135. Hogg K, Morton R: **Alternative treatments for neck sprain.** *Emerg Med J* 2003, **20:**62.
136. Holdcraft LC, Assefi N, Buchwald D: **Complementary and alternative medicine in fibromyalgia and related syndromes.** *Best Pract Res Clin Rheumatol* 2003, **17:**667-683.
137. Hoskins WT, Polland HP: **Successful management of hamstring injuries in Australian Rules footballers: Two case reports.** *Chiropr Osteopat* 2005, **13**.
138. Humphreys BK: **Possible adverse events in children treated by manual therapy: a review.** *Chiropr Osteopat* 2010, **18:**12.
139. Hurwitz EL: **Epidemiology: spinal manipulation utilization.** *J Electromyogr Kinesiol* 2012, **22:**648-654.
140. Inamasu J, Guiot BH: **Iatrogenic vertebral artery injury.** *Acta Neurol Scand* 2005, **112:**349-357.
141. Isabel de-la-Llave-Rincon A, Puentedura EJ, Fernandez-de-Las-Penas C: **Clinical presentation and manual therapy for upper quadrant musculoskeletal conditions.** *J Man Manip Ther* 2011, **19:**201-211.
142. Jeffery AR, Ellis FJ, Repka MX, Buncic JR: **Pediatric Horner syndrome.** *J AAPOS* 1998, **2:**159-167.

143. Johnson AW, Shubrook JH, Jr.: **Role of osteopathic structural diagnosis and osteopathic manipulative treatment for diabetes mellitus and its complications.** *J Am Osteopath Assoc* 2013, **113:**829-836.
144. Johnson C: **Comparative effectiveness research and the chiropractic profession.** *J Manipulative Physiol Ther* 2010, **33:**243-250.
145. Johnston WL: **Segmental definition: Part II. Application of an indirect method in osteopathic manipulative treatment.** *J Am Osteopath Assoc* 1988, **88:**211-217.
146. Jordan A, Ostergaard K: **Implementation of neck/shoulder rehabilitation in primary health care clinics.** *J Manipulative Physiol Ther* 1996, **19:**36-40.
147. Jordan A, Ostergaard K: **Rehabilitation of neck/shoulder patients in primary health care clinics.** *J Manipulative Physiol Ther* 1996, **19:**32-35.
148. Jordan J, Konstantinou K, O'Dowd J: **Herniated lumbar disc.** *BMJ Clin Evid* 2009, **2009**.
149. Jordan J, Konstantinou K, O'Dowd J: **Herniated lumbar disc.** *BMJ Clin Evid* 2011, **2011**.
150. Kalamir A, Pollard H, Vitiello AL, Bonello R: **Manual therapy for temporomandibular disorders: A review of the literature.** *J Bodyw Mov Ther* 2007, **11:**84-90.
151. Katavich L: **Differential effects of spinal manipulative therapy on acute and chronic muscle spasm: A proposal for mechanisms and efficacy.** *Man Ther* 1998, **3:**132-139.
152. Koes BW, Assendelft WJ, Heijden GJ, Bouter LM: **Spinal manipulation for low back pain: an updated systematic review of randomized clinical trials.** *Spine (Phila Pa 1976)* 1996, **21:**2860-2871.
153. Koes BW, Assendelft WJ, van der Heijden GJ, Bouter LM, Knipschild PG: **Spinal manipulation and mobilisation for back and neck pain: a blinded review.** *BMJ* 1991, **303:**1298-1303.
154. Kohlbeck FJ, Haldeman S: **Medication-assisted spinal manipulation.** *Spine J* 2002, **2:**288-302.
155. Korr IM: **Osteopathic research: the needed paradigm shift.** *J Am Osteopath Assoc* 1991, **91:**156, 161-158, 170-151.
156. Kreitz BG, Aker PD: **Nocturnal enuresis: treatment implications for the chiropractor.** *J Manipulative Physiol Ther* 1994, **17:**465-473.
157. Krishnan A, Silver N: **Headache (chronic tension-type).** *BMJ Clin Evid* 2009, **2009**.
158. Kwan I, Onwude JL: **Premenstrual syndrome.** *BMJ Clin Evid* 2007, **2007**.
159. Ladeira CE: **Evidence based practice guidelines for management of low back pain: physical therapy implications.** *Rev Bras Fisioter* 2011, **15:**190-199.
160. Latthe PM, Champaneria R, Khan KS: **Dysmenorrhoea.** *BMJ Clin Evid* 2011, **2011**.
161. Lawrence DJ, Meeker WC: **Chiropractic and CAM utilization: a descriptive review.** *Chiropr Osteopat* 2007, **15**.
162. Lawrence V: **Spinal manipulation for low-back pain: A meta-analysis.** *Ann Intern Med* 1993, **118:**49.
163. Leboeuf-Yde C, Hestbaek L: **Chiropractic and children: Is more research enough?** *Chiropr Osteopat* 2010, **18**.
164. Lefebvre R, Peterson D, Haas M: **Evidence-Based Practice and Chiropractic Care.** *J Evid Based Complementary Altern Med* 2012, **18:**75-79.
165. Lehman JJ, Jegtvig SK: **Reactive attachment disorder: a preventable mental health disease.** *J Chiropr Med* 2004, **3:**69-75.
166. Levi JR, Brody RM, McKee-Cole K, Pribitkin E, O'Reilly R: **Complementary and alternative medicine for pediatric otitis media.** *Int J Pediatr Otorhinolaryngol* 2013, **77:**926-931.
167. Lewis BJ, Carruthers G: **Systematic review of spinal manipulation: A biased report.** *J R Soc Med* 2006, **99:**278; author reply 279-280.
168. Licciardone JC: **Osteopathic research: elephants, enigmas, and evidence.** *Osteopath Med Prim Care* 2007, **1:**7.
169. Licciardone JC: **Responding to the challenge of clinically relevant osteopathic research: efficacy and beyond.** *Int J Osteopath Med* 2007, **10:**3.

170. Licciardone JC: **Systematic review and meta-analysis conclusions relating to osteopathic manipulative treatment for low back pain remain valid and well accepted.** *J Bodyw Mov Ther* 2013, **17:**2-4.
171. Lucaciu OC, Connell GP: **Itch sensation through transient receptor potential channels: a systematic review and relevance to manual therapy.** *J Manipulative Physiol Ther* 2013, **36:**385-393.
172. Lucassen P: **Infantile colic.** *BMJ Clin Evid* 2007, **2007**.
173. Lucassen P: **Colic in infants.** *BMJ Clin Evid* 2010, **2010**.
174. Lucassen P: **Colic in infants.** *BMJ Clin Evid* 2015, **2015**.
175. Maigne JY, Goussard JC, Dumont F, Marty M, Berlinson G: **[Is systematic radiography needed before spinal manipulation? Recommendations of the SOFMMOO].** *Ann Readapt Med Phys* 2007, **50:**111-116; discussion 117-118.
176. Majchrzycki M, Wolski H, Seremak-Mrozikiewicz A, Lipiec J, Marszalek S, Mrozikiewicz PM, Klejewski A, Lisinski P: **Application of osteopathic manipulative technique in the treatment of back pain during pregnancy.** *Ginekol Pol* 2015, **86:**224-228.
177. Manchikanti L, Falco FJ, Benyamin RM, Caraway DL, Kaye AD, Helm S, 2nd, Wargo BW, Hansen H, Parr AT, Singh V, et al: **Assessment of bleeding risk of interventional techniques: a best evidence synthesis of practice patterns and perioperative management of anticoagulant and antithrombotic therapy.** *Pain Physician* 2013, **16:**SE261-318.
178. Manniche C, Bendix T: **[Back pain--from the viewpoint of medical technology. Danish National Board of Health].** *Nord Med* 1998, **113:**230-232, 239.
179. Marchand AM: **A Literature Review of Pediatric Spinal Manipulation and Chiropractic Manipulative Therapy: Evaluation of Consistent Use of Safety Terminology.** *J Manipulative Physiol Ther* 2012.
180. Marchand AM: **A Proposed Model With Possible Implications for Safety and Technique Adaptations for Chiropractic Spinal Manipulative Therapy for Infants and Children.** *J Manipulative Physiol Ther* 2013.
181. May S, Rosedale R: **Prescriptive clinical prediction rules in back pain research: a systematic review.** *J Man Manip Ther* 2009, **17:**36-45.
182. McGrath MC: **A global view of osteopathic practice - mirror or echo chamber?** *Int J Osteopath Med* 2015, **18:**130-140.
183. McIntosh G, Hall H: **Low back pain (acute).** *BMJ Clin Evid* 2011, **2011**.
184. Miners AL: **Chiropractic treatment and the enhancement of sport performance: a narrative literature review.** *J Can Chiropr Assoc* 2010, **54:**210-221.
185. Mior S: **Manipulation and mobilization in the treatment of chronic pain.** *Clin J Pain* 2001, **17:**S70-76.
186. Mirtz TA: **Acute respiratory distress syndrome: clinical recognition and preventive management in chiropractic acute care practice.** *J Manipulative Physiol Ther* 2001, **24:**467-473.
187. Mirtz TA, Perle SM: **The prevalence of the term subluxation in North American English-Language Doctor of chiropractic programs.** *Chiropr Man Therap* 2011, **19:**14.
188. Mitchell J: **Vertebral Artery Blood flow Velocity Changes Associated with Cervical Spine rotation: A Meta-Analysis of the Evidence with implications for Professional Practice.** *J Man Manip Ther* 2009, **17:**46-57.
189. Moore A: **Systematic review of spinal manipulation: Including different techniques.** *J R Soc Med* 2006, **99:**278-279; author reply 279-280.
190. Morningstar MW, Joy T: **Scoliosis treatment using spinal manipulation and the Pettibon Weighting SystemTM: A summary of 3 atypical presentations.** *Chiropr Osteopat* 2006, **14**.
191. Mueller S, Sahs AL: **Brain stem dysfunction related to cervical manipulation. Report of 3 cases.** *Neurology* 1976, **26:**547-550.
192. Murphy DR: **Current understanding of the relationship between cervical manipulation and stroke: What does it mean for the chiropractic profession?** *Chiropr Osteopat* 2010, **18**.

193. Murphy DR, Goldstein D, Katz M: **Chiropractic adjustment to the cervical spine and the Arnold-Chiari malformation.** *J Manipulative Physiol Ther* 1993, **16:**550-555.
194. Murphy DR, Schneider MJ, Seaman DR, Perle SM, Nelson CF: **How can chiropractic become a respected mainstream profession? The example of podiatry.** *Chiropr Osteopat* 2008, **16**.
195. n.a.: **Nonsurgical treatment is effective for carpal tunnel syndrome.** *J Fam Pract* 2004, **53:**685.
196. Nachemson A: **Back pain - causes, diagnosis, treatment (Structured abstract).** In *Health Technology Assessment Database*. pp. 200; 1991:200.
197. Nalley C, Castellvi A, Abitbol JJ: **Current status of lumbar disc replacements and motion preservation devices.** *Curr Orthop Pract* 2014, **25:**4-8.
198. Nansel D, Szlazak M: **Somatic dysfunction and the phenomenon of visceral disease simulation: a probable explanation for the apparent effectiveness of somatic therapy in patients presumed to be suffering from true visceral disease.** *J Manipulative Physiol Ther* 1995, **18:**379-397.
199. Nelson CF, Lawrence DJ, Triano JJ, Bronfort G, Perle SM, Metz RD, Hegetschweiler K, LaBrot T: **Chiropractic as spine care: A model for the profession.** *Chiropr Osteopat* 2005, **13**.
200. Nielsen JB, Crone C, Hultborn H: **The spinal pathophysiology of spasticity--from a basic science point of view.** *Acta Physiol (Oxf)* 2007, **189:**171-180.
201. Nyiendo J, Haas M, Hondras MA: **Outcomes research in chiropractic: the state of the art and recommendations for the chiropractic research agenda.** *J Manipulative Physiol Ther* 1997, **20:**185-200.
202. Oakley PA, Harrison DD, Harrison DE, Haas JW: **Evidence-based protocol for structural rehabilitation of the spine and posture: review of clinical biomechanics of posture (CBP) publications.** *J Can Chiropr Assoc* 2005, **49:**270-296.
203. O'Neill A, Willis E: **Chiropractic and the politics of health care.** *Aust J Public Health* 1994, **18:**325-331.
204. O'Shaughnessy J, Drolet M, Roy J, Descarreaux M: **Chiropractic management of patients post-disc arthroplasty: Eight case reports.** *Chiropr Osteopat* 2010, **18**.
205. Osterbauer PJ, Fuhr AW, Hildebrandt RW: **Mechanical force, manually assisted short lever chiropractic adjustment.** *J Manipulative Physiol Ther* 1992, **15:**309-317.
206. Ottenbacher K, DiFabio RP: **Efficacy of spinal manipulation/mobilization therapy. A meta-analysis.** *Spine (Phila Pa 1976)* 1985, **10:**833-837.
207. Pelletier JC: **Sports related concussion and spinal injuries: the need for changing spearing rules at the National Capital Amateur Football Association (NCAFA).** *J Can Chiropr Assoc* 2006, **50:**195-208.
208. Pelletier KR, Astin JA: **Integration and reimbursement of complementary and alternative medicine by managed care and insurance providers: 2000 update and cohort analysis.** *Altern Ther Health Med* 2002, **8:**38-39, 42, 44 passim.
209. Pelletier KR, Marie A, Krasner M, Haskell WL: **Current trends in the integration and reimbursement of complementary and alternative medicine by managed care, insurance carriers, and hospital providers.** *Am J Health Promot* 1997, **12:**112-122.
210. Petering RC, Webb C: **Treatment options for low back pain in athletes.** *Sports Health* 2011, **3:**550-555.
211. Pfaffenrath V, Brune K, Diener HC, Gerber WD, Gobel H: **Treatment of tension-type headache. [German].** *MMW Munch Med Wochenschr* 1998, **140:**519-525.
212. Pfaffenrath V, Brune K, Diener HC, Gerber WD, Gobel H: **The treatment of tension-type headache. Guidelines of the German migraine- and headache society. [German].** *Nervenheilkunde* 1998, **17:**91-100.
213. Pfaffenrath V, Brune K, Diener HC, Gerber WD, Gobel H: **Treatment of tension-type headache. Recommendations of the German migraine and headache society. [German].** *Schmerz* 1998, **12:**156-168.
214. Pickar JG: **Neurophysiological effects of spinal manipulation.** *Spine J* 2002, **2:**357-371.
215. Pickar JG: **Neurophysiological effects of spinal manipulation. [Spanish].** *Osteopatia Cientifica* 2011, **6:**2-18.

216. Pickar JG, Bolton PS: **Spinal manipulative therapy and somatosensory activation.** *J Electromyogr Kinesiol* 2012, **22:**785-794.
217. Pizzolorusso G, Cerritelli F, D'Orazio M, Cozzolino V, Turi P, Renzetti C, Barlafante G, D'Incecco C: **Osteopathic evaluation of somatic dysfunction and craniosacral strain pattern among preterm and term newborns.** *J Am Osteopath Assoc* 2013, **113:**462-467.
218. Pohlman KA, Holton-Brown MS: **Otitis media and spinal manipulative therapy: a literature review.** *J Chiropr Med* 2012, **11:**160-169.
219. Pollard H, de Luca K: **A descriptive report of management strategies used by chiropractors, as reviewed by a single independent chiropractic consultant in the Australian workers compensation system.** *Chiropr Osteopat* 2009, **17**.
220. Pollard H, Hoskins W, McHardy A, Bonello R, Garbutt P, Swain M, Dragasevic G, Pribicevic M, Vitiello A: **Australian chiropractic sports medicine: Half way there or living on a prayer?** *Chiropr Osteopat* 2007, **15**.
221. Posadzki P, Ernst E: **Spinal manipulation: an update of a systematic review of systematic reviews.** *N Z Med J* 2011, **124:**55-71.
222. Proctor ML, Farquhar CM: **Dysmenorrhoea.** *BMJ Clin Evid* 2007, **2007**.
223. Pustaver MR: **Mechanical low back pain: etiology and conservative management.** *J Manipulative Physiol Ther* 1994, **17:**376-384.
224. Quinn D: **Response to article "Spinal manipulation: an update of a systematic review of systematic reviews".** *N Z Med J* 2011, **124:**117-119.
225. Raineteau O: **Plastic responses to spinal cord injury.** *Behav Brain Res* 2008, **192:**114-123.
226. Raineteau O, Schwab ME: **Plasticity of motor systems after incomplete spinal cord injury.** *Nat Rev Neurosci* 2001, **2:**263-273.
227. Reggars JW: **Recording techniques and analysis of the articular crack. A critical review of the literature.** *Australas Chiropr Osteopathy* 1996, **5:**86-92.
228. Reggars JW: **The therapeutic benefit of the audible release associated with spinal manipulative therapy. A critical review of the literature.** *Australas Chiropr Osteopathy* 1998, **7:**80-85.
229. Renckens CN: **[Systematic review of the effects of therapy in infants with the KISS-syndrome (kinetic imbalance due to suboccipital strain)].** *Ned Tijdschr Geneeskd* 2005, **149:**1237; author reply 1237-1238.
230. Retzlaff EW: **Reflex mechanisms and their clinical significance.** *Osteopath Ann* 1974, **2:**40-43.
231. Rodine RJ, Vernon H: **Cervical radiculopathy: a systematic review on treatment by spinal manipulation and measurement with the Neck Disability Index.** *J Can Chiropr Assoc* 2012, **56:**18-28.
232. Rosted P, Andersen C: **[Use of stimulation techniques in pain treatment].** *Ugeskr Laeger* 2006, **168:**1982-1986.
233. Rubinstein SM, van Eekelen R, Oosterhuis T, de Boer MR, Ostelo RW, van Tulder MW: **The risk of bias and sample size of trials of spinal manipulative therapy for low back and neck pain: analysis and recommendations.** *J Manipulative Physiol Ther* 2014, **37:**523-541.
234. Rupert R, Gotlib A: **Chiropractic manipulation in pediatric health conditions - An updated systematic review.** *Chiropr Osteopat* 2008, **16**.
235. Rupert RL: **Searching chiropractic literature: a comparison of three computerized databases.** *J Manipulative Physiol Ther* 1997, **20:**285-288.
236. Russell R: **The rationale for primary spine care employing biopsychosocial, stratified and diagnosis-based care-pathways at a chiropractic college public clinic: A literature review.** *Chiropr Man Therap* 2013, **21**.
237. Saedt E, van der Woude B, Theunissen P: **[Systematic review of the effects of therapy in infants with the KISS-syndrome (kinetic imbalance due to suboccipital strain)].** *Ned Tijdschr Geneeskd* 2005, **149:**1238-1239; author reply 1239.
238. Salehi A, Hashemi N, Imanieh MH, Saber M: **Chiropractic: Is it Efficient in Treatment of Diseases? Review of Systematic Reviews.** *Int J Community Based Nurs Midwifery* 2015, **3:**244-254.

239. Savva C, Giakas G, Efstathiou M: **The role of the descending inhibitory pain mechanism in musculoskeletal pain following high-velocity, low amplitude thrust manipulation: a review of the literature.** *J Back Musculoskelet Rehabil* 2014, **27:**377-382.
240. Saxler G, Schopphoff E, Quitmann H, Quint U: **[Spinal manipulative therapy and cervical artery dissections].** *HNO* 2005, **53:**563-567.
241. Schildt-Rudloff K: **The value of the musculature in manual medicine. [German].** *Manuelle Medizin* 1995, **33:**101-106.
242. Schmerl M, Pollard H, Hoskins W: **Labral injuries of the hip: a review of diagnosis and management.** *J Manipulative Physiol Ther* 2005, **28:**632.e631-638.
243. Seaman DR, Cleveland C, 3rd: **Spinal pain syndromes: nociceptive, neuropathic, and psychologic mechanisms.** *J Manipulative Physiol Ther* 1999, **22:**458-472.
244. Sejari N, Kamaruddin K, Al-Worafi YMA, Ming LC: **A narrative review of massage and spinal manipulation in the treatment of low back pain.** *Arch Pharm Pract* 2014, **5:**139-143.
245. Shaw L, Descarreaux M, Bryans R, Duranleau M, Marcoux H, Potter B, Ruegg R, Watkin R, White E: **A systematic review of chiropractic management of adults with Whiplash-Associated Disorders: recommendations for advancing evidence-based practice and research.** *Work* 2010, **35:**369-394.
246. Shekelle PG, Coulter I: **Cervical spine manipulation: summary report of a systematic review of the literature and a multidisciplinary expert panel.** *J Spinal Disord* 1997, **10:**223-228.
247. Shiple BJ: **Treating low-back pain: exercise knowns and unknowns.** *Phys Sportsmed* 1997, **25:**51-66.
248. Sigg DC, Falkenberg JH, Hausmann ON, Iaizzo PA: **Low back pain: Part III: Treatment approaches.** *Progress in Anesthesiology* 2000, **14:**183-200.
249. Silber J: **Cochrane review of manipulation and mobilization for mechanical neck disorders.** *Spine (Phila Pa 1976)* 2005, **30:**166.
250. Simpson JK: **The Five Eras of Chiropractic & the future of chiropractic as seen through the eyes of a participant observer.** *Chiropr Man Therap* 2012, **20**.
251. Simpson JK, Hawken E: **Xiphodynia: A diagnostic conundrum.** *Chiropr Osteopat* 2007, **15**.
252. Smart LJ, Jr., Smith DL: **Postural dynamics: clinical and empirical implications.** *J Manipulative Physiol Ther* 2001, **24:**340-349.
253. Snodgrass SJ, Haskins R, Rivett DA: **A structured review of spinal stiffness as a kinesiological outcome of manipulation: its measurement and utility in diagnosis, prognosis and treatment decision-making.** *J Electromyogr Kinesiol* 2012, **22:**708-723.
254. Snow GJ: **Chiropractic management of a patient with lumbar spinal stenosis.** *J Manipulative Physiol Ther* 2001, **24:**300-304.
255. Solomon DH, Bates DW, Panush RS, Katz JN: **Costs, outcomes, and patient satisfaction by provider type for patients with rheumatic and musculoskeletal conditions: a critical review of the literature and proposed methodologic standards.** *Ann Intern Med* 1997, **127:**52-60.
256. Spadini E: **Value and average cost in manual medicine. [Italian].** *Riabilitazione* 1998, **31:**199-203.
257. Spears LG: **A narrative review of medical, chiropractic, and alternative health practices in the treatment of primary dysmenorrhea.** *J Chiropr Med* 2005, **4:**76-88.
258. Stock JL, Amantea L, Overdorf JH, Samar AD, Pickens FL: **The role of chiropractic in the diagnosis, prevention, and treatment of osteoporosis.** *Complement Ther Med* 1997, **5:**36-39.
259. Stuber K, Sajko S, Kristmanson K: **Chiropractic treatment of lumbar spinal stenosis: a review of the literature.** *J Chiropr Med* 2009, **8:**77-85.
260. Suchowersky O, Gronseth G, Perlmutter J, Reich S, Zesiewicz T, Weiner WJ: **Practice parameter: neuroprotective strategies and alternative therapies for Parkinson disease (an evidence-based review). Report of the Quality Standards Subcommittee of the American Academy of Neurology.** *Neurology* 2006, **66:**976-982.
261. Szabela DA, Szabela MA, Baumgartner H: **Post manipulation complications in treatment of the spine. An overview of the literature from 1989-1996. [German].** *Manuelle Medizin* 1997, **35:**258-262.

262. Taylor DN: **A theoretical basis for maintenance spinal manipulative therapy for the chiropractic profession.** *J Chiropr Humanit* 2011, **18:**74-85.
263. Terrett A: **The search for the subluxation: an investigation of medical literature to 1985.** *Chiropr Hist* 1987, **7:**29-33.
264. Triano JJ: **Biomechanics of spinal manipulative therapy.** *Spine J* 2001, **1:**121-130.
265. Troyanovich SJ, Harrison DE, Harrison DD: **Structural rehabilitation of the spine and posture: rationale for treatment beyond the resolution of symptoms.** *J Manipulative Physiol Ther* 1998, **21:**37-50.
266. Tuchin P: **A replication of the study 'Adverse effects of spinal manipulation: a systematic review'.** *Chiropr Man Therap* 2012, **20**.
267. Vallone SA, Miller J, Larsdotter A, Barham-Floreani J: **Chiropractic approach to the management of children.** *Chiropr Osteopat* 2010, **18**.
268. van de Veen EA, de Vet HC, Pool JJ, Schuller W, de Zoete A, Bouter LM: **Variance in manual treatment of nonspecific low back pain between orthomanual physicians, manual therapists, and chiropractors.** *J Manipulative Physiol Ther* 2005, **28:**108-116.
269. van Tulder MW, Furlan AD, Gagnier JJ: **Complementary and alternative therapies for low back pain.** *Best Pract Res Clin Rheumatol* 2005, **19:**639-654.
270. Vautravers P, Maigne JY: **[Cervical spine manipulation: risks--benefit--assessment].** *Rev Neurol (Paris)* 2003, **159:**1064-1066.
271. Vernon H: **Qualitative review of studies of manipulation-induced hypoalgesia.** *J Manipulative Physiol Ther* 2000, **23:**134-138.
272. Vernon H: **The effectiveness of spinal manipulation for the treatment of headache disorders: a systematic review of randomized clinical trials.** *Cephalalgia* 2003, **23:**479-480; author reply 480-471.
273. Vernon H: **What is different about spinal pain?** *Chiropr Man Therap* 2012, **20:**22.
274. Vernon H, Puhl A, Reinhart C: **Systematic review of clinical trials of cervical manipulation: control group procedures and pain outcomes.** *Chiropr Man Therap* 2011, **19:**3.
275. Vernon H, Steiman I, Crnec M, Thiel H, Kitchen R: **Efficacy of spinal manipulation/mobilization: a meta-analysis.** *Spine (Phila Pa 1976)* 1986, **11:**973-974.
276. Vernon HT: **The effectiveness of chiropractic manipulation in the treatment of headache: an exploration in the literature.** *J Manipulative Physiol Ther* 1995, **18:**611-617.
277. Victoria Espi-Lopez G, Arnal-Gomez A, Arbos-Berenguer T, Gonzalez AA, Vicente-Herrero T: **Effectiveness of Physical Therapy in Patients with Tension-type Headache: Literature Review.** *J Jpn Phys Ther Assoc* 2014, **17:**31-38.
278. Vijayalakshmi N, Paramanandam P: **Complementary therapies in asthmatic children.** *Int J Pharma Bio Sci* 2015, **6:**B719-B726.
279. Vohra S, Johnston BC, Cramer K, Humphreys K: **Adverse events associated with pediatric spinal manipulation: a systematic review.** *Pediatrics* 2007, **119:**e275-283.
280. Vohra S, Johnston BC, Cramer K, Humphreys K: **Erratum: Adverse events associated with pediatric spinal manipulation: A systematic review (Pediatrics (2007) 119, (e275-e283) DOI: 10.1542/peds.2006- 1392).** *Pediatrics* 2007, **119:**867.
281. Vohra S, Johnston BC, Cramer K, Humphreys K: **Erratum: Adverse events associated with pediatric spinal manipulation: A systematic review (Pediatrics (January 2001) 119, (e275-e283) DOI: 10.1542/peds.2006-1392).** *Pediatrics* 2007, **120:**251.
282. Wakefield TS, Huhner DA, David Gruszka DC: **On-site documentation of sports-related injuries.** *Journal of Sports Chiropractic and Rehabilitation* 1997, **11:**156-162.
283. Walker BF: **Spinal stenosis: a summary and review.** *COMSIG Rev* 1993, **2:**49-52.
284. Walker LA, Budd S: **UK: the current state of regulation of complementary and alternative medicine.** *Complement Ther Med* 2002, **10:**8-13.

285. Wang HH, Zhan HS, Zhang MC, Chen B, Guo K: **[Retrospective analysis and prevention strategies for accidents associated with cervical manipulation in China].** *Zhongguo Gu Shang* 2012, **25:**730-736.
286. Ward RC, Hruby RJ, Falls WM: **Integrating cranial nerve and release enhancing exercises assist several manipulative methods when treating a subset of head and neck pain patients. [German].** *Manuelle Medizin* 1998, **36:**182-193.
287. Weiner DK, Ernst E: **Complementary and alternative approaches to the treatment of persistent musculoskeletal pain.** *Clin J Pain* 2004, **20:**244-255.
288. Wellington J: **Noninvasive and alternative management of chronic low back pain (efficacy and outcomes).** *Neuromodulation* 2014, **17 Suppl 2:**24-30.
289. Wells RE: **Spinal manipulation for headaches: will better quality trials do the trick?** *Headache* 2011, **51:**1149-1151.
290. Wenban AB: **Inappropriate use of the title 'chiropractor' and term 'chiropractic manipulation' in the peer-reviewed biomedical literature.** *Chiropr Osteopat* 2006, **14:**16.
291. Whedon JM, Glassey D: **Cerebrospinal fluid stasis and its clinical significance.** *Altern Ther Health Med* 2009, **15:**54-60.
292. White AR, Ernst E: **Economic analysis of complementary medicine: a systematic review.** *Complement Ther Med* 2000, **8:**111-118.
293. Williams NH: **Optimising the psychological benefits of osteopathy.** *Int J Osteopath Med* 2007, **10:**36-41.
294. Williams S: **Potential unique causes of burnout for chiropractic professionals.** *J Chiropr Humanit* 2011, **18:**86-93.
295. Wingfield BR, Gorman RF: **Treatment of severe glaucomatous visual field deficit by chiropractic spinal manipulative therapy: a prospective case study and discussion.** *J Manipulative Physiol Ther* 2000, **23:**428-434.
296. Wolpaw JR, Carp JS: **Plasticity from muscle to brain.** *Prog Neurobiol* 2006, **78:**233-263.
297. Woodbury A, Soong SN, Fishman D, Garcia PS: **Complementary and alternative medicine therapies for the anesthesiologist and pain practitioner: a narrative review.** *Can J Anaesth* 2015.
298. Wye L, Sharp D, Shaw A: **The impact of NHS based primary care complementary therapy services on health outcomes and NHS costs: a review of service audits and evaluations.** *BMC Complement Altern Med* 2009, **9:5**.
299. Young KJ: **Gimme that old time religion: the influence of the healthcare belief system of chiropractic's early leaders on the development of x-ray imaging in the profession.** *Chiropr Man Therap* 2014, **22:**36.
300. Young M: **Safety of chiropractic interventions: a systematic review.** *Spine (Phila Pa 1976)* 2009, **34:**2476; author reply 2476-2477.
301. Zheng Z, Xue CC: **Pain research in complementary and alternative medicine in Australia: a critical review.** *J Altern Complement Med* 2013, **19:**81-91.

From updated search:

1. Task_Force_on_the_Low_Back_Pain_Clinical_Practice_Guidelines: **American Osteopathic Association Guidelines for Osteopathic Manipulative Treatment (OMT) for Patients With Low Back Pain.** *J Am Osteopath Assoc* 2016, **116:**536-549.
2. Fernandez-de-Las-Penas C, Cuadrado ML: **Physical therapy for headaches.** *Cephalalgia* 2015.
3. Karpouzis F, Bonello R, Pribicevic M, Kalamir A, Brown BT: **Quality of reporting of randomised controlled trials in chiropractic using the CONSORT checklist.** *Chiropr Man Therap* 2016, **24:**19.
4. Marom T, Marchisio P, Tamir SO, Torretta S, Gavriel H, Esposito S: **Complementary and Alternative Medicine Treatment Options for Otitis Media: A Systematic Review.** *Medicine (Baltimore)* 2016, **95:**e2695.

5. Todd AJ, Carroll MT, Mitchell EK: **Forces of Commonly Used Chiropractic Techniques for Children: A Review of the Literature.** *J Manipulative Physiol Ther* 2016, **39:**401-410.
6. Zhang L, Yao CH: **The Physiological Role of Tumor Necrosis Factor in Human Immunity and Its Potential Implications in Spinal Manipulative Therapy: A Narrative Literature Review.** *J Chiropr Med* 2016, **15:**190-196.
7. Karas S, Pannone A: **T4 Syndrome: A Scoping Review of the Literature.** *J Manipulative Physiol Ther* 2016.
8. Bussieres AE, Al Zoubi F, Stuber K, French SD, Boruff J, Corrigan J, Thomas A: **Evidence-based practice, research utilization, and knowledge translation in chiropractic: a scoping review.** *BMC Complement Altern Med* 2016, **16:**216.
9. Gorrell LM, Engel RM, Brown B, Lystad RP: **The reporting of adverse events following spinal manipulation in randomized clinical trials-a systematic review.** *Spine J* 2016, **16:**1143-1151.
10. Castellini G, Gianola S, Banfi G, Bonovas S, Moja L: **Mechanical Low Back Pain: Secular Trend and Intervention Topics of Randomized Controlled Trials.** *Physiother Can* 2016, **68:**61-63.
11. Weiss HR, Moramarco MM, Borysov M, Ng SY, Lee SG, Nan X, Moramarco KA: **Postural Rehabilitation for Adolescent Idiopathic Scoliosis during Growth.** *Asian Spine J* 2016, **10:**570-581.
12. Morell GC: **Manual therapy improved signs and symptoms of temporomandibular disorders.** *Evid Based Dent* 2016, **17:**25-26.
13. Bagagiolo D, Didio A, Sbarbaro M, Priolo CG, Borro T, Farina D: **Osteopathic Manipulative Treatment in Pediatric and Neonatal Patients and Disorders: Clinical Considerations and Updated Review of the Existing Literature.** *Am J Perinatol* 2016, **33:**1050-1054.

Language (16 records and no records*)

No full-text or abstract available in English, Swedish, Norwegian or Danish (3 records)

1. Cover s: **The search for the first 15 'disciples'.** *Chiropr Hist* 1983, **3:**23-24.
2. Lakke SE, Dolder R, Rijn M, Verhagen AP: **The effect of adding mobilization and manipulation to exercise therapy in patients with chronic low back pain: a systematic review.** *Nederlands Tijdschrift voor Fysiotherapie* 2009, **119:**170-176.
3. Lance J-MR: **Direct access to chiropractic care for car-accident victims with whiplash - Feasibility analysis for a meta-analysis (Structured abstract).** In *Health Technology Assessment Database*: Agence d'Evaluation des Technologies et des Modes d'Intervention en Sante (AETMIS); 2003.

English abstract available, but provides too few details (13 records)

1. Bu JH, Kong LJ, Guo CQ, Yang XC, Cheng YW: **Effectiveness of manual therapy and traction for lumbar disc herniation: a meta-analysis (Provisional abstract).** In *Database of Abstracts of Reviews of Effects*. pp. 409-414; 2014:409-414.
2. Dai DC, Fang M: **Normalized analysis on tuina manipulation applied in treating degenerative spondylolisthesis. [Chinese].** *Chinese Journal of Clinical Rehabilitation* 2006, **10:**160-162.
3. Dai DC, Fang M, Shen GQ, Yan JT: **Research on the characteristics of sacroiliac joint dysfunction and interventional treatment of tuina. [Chinese].** *Chinese Journal of Clinical Rehabilitation* 2006, **10:**135-138.

4. Gamus D: **[Advances in research of complementary and integrative medicine: a review of recent publications in some of the leading medical journals].** *Harefuah* 2015, **154:**9-15, 70.
5. Hoving JL, Heijden GJ: **Fysiotherapie bij heupklachten: systematische review van klinisch effectonderzoek.** *Nederlands Tijdschrift fur Fysioterapie* 1997, **107:**2-7.
6. Lahad A, Sarig-Bahat H: **[Israeli guidelines for prevention of low back pain].** *Harefuah* 2007, **146:**253-257, 320.
7. Li JB, Xiong QL, Qu SK, He JZ, Deng Y, Jia T, Li Y, Yi HC: **Application of Tuina manipulation for lumbar disc herniation: Literature analysis in recent 10 years. [Chinese].** *Chinese Journal of Tissue Engineering Research* 2014, **18:**7211-7216.
8. Lu Y, Wang XF: **Treatment of pediatric cerebral palsy with massage along with meridian, massage on special locus and acupressure. [Chinese].** *Chinese Journal of Clinical Rehabilitation* 2005, **9:**158-159.
9. Pribicevic M, Pollar H, Bonell R, de Luca K: **A systematic review of manipulative therapy for the treatment of shoulder pain. [Spanish].** *Osteopatia Cientifica* 2011, **6:**86-97.
10. Qin Dp, Zhang Xg, Song M: **Application of finite element analysis in mechanism of lumbar disease by Chinese medicine bonesetting massage manipulation.** *Chinese Journal of Tissue Engineering Research* 2012, **16:**4913-4917.
11. Ye RB, Zhou JX, Gan MX: **[Clinical and CT analysis of 35 cases of lumbar disc herniation before and after non-operative treatment].** *Zhong Xi Yi Jie He Za Zhi* 1990, **10:**667-668, 645.
12. Zhou X, Bai YH: **Combined therapy of traditional Chinese medicine and western medicine for low back pain. [Chinese].** *Chinese Journal of Clinical Rehabilitation* 2006, **10:**118-120.
13. Zhu GM, Fang M, Sun WQ: **Mechanism and characteristics of the rotary manipulation in the treatment of cervical spondylotic radiculopathy. [Chinese].** *Chinese Journal of Clinical Rehabilitation* 2006, **10:**154-156.

Not retrievable (32 records and 8 records*)

Only protocol available (22 records and 8 records*)

1. Abdul Rani Aidatul A, Ab Ghani Rimah M, Shamsuddin S, Abdullah Z, Abdul Halim Nur H, Mustapha N, Muhamad Nor A: **Massage therapy for improving functional activity after stroke.** In *Cochrane Database Syst Rev*: John Wiley & Sons, Ltd; 2015.
2. Blanchette MA, Bussieres A, Stochkendahl MJ, Boruff J, Harrison P: **Effectiveness and economic evaluation of chiropractic care for the treatment of low back pain: a systematic review protocol.** *Syst Rev* 2015, **4:**30.
3. Brønfort G, Evans Roni L, Goldsmith Charles H, Haas M, Leininger B, Levin M, Schmitt J, Westrom K: **Spinal rehabilitative exercise and manual treatment for the prevention of migraine attacks in adults.** In *Cochrane Database Syst Rev*: John Wiley & Sons, Ltd; 2015.
4. Catala-Lopez F, Hutton B, Nunez-Beltran A, Mayhew AD, Page MJ, Ridao M, Tobias A, Catala MA, Tabares-Seisdedos R, Moher D: **The pharmacological and non-pharmacological treatment of attention deficit hyperactivity disorder in children and adolescents: protocol for a systematic review and network meta-analysis of randomized controlled trials.** *Syst Rev* 2015, **4:**19.
5. Craane B, De Laat A, Dijkstra Pieter U, Stappaerts K, Stegenga B: **Physical therapy for the management of patients with temporomandibular disorders and related pain.** In *Cochrane Database Syst Rev*: John Wiley & Sons, Ltd; 2006.

6. French Helen P, Galvin R, Abbott JH, Fransen M: **Adjunctive therapies in addition to land-based exercise therapy for osteoarthritis of the hip or knee.** In *Cochrane Database Syst Rev*: John Wiley & Sons, Ltd; 2015.
7. Gutiérrez Jorge A, Soto C, Rada G: **Physical therapy interventions for the prevention of fractures after spinal cord injury.** In *Cochrane Database Syst Rev*: John Wiley & Sons, Ltd; 2013.
8. Hajebrahimi S, Sadeghi-Bazargani H, Taleschian Tabrizi N, Farhadi F, Sadeghi Ghyassi F: **Non-drug treatment for lower urinary tract symptoms in women with voiding dysfunction.** In *Cochrane Database Syst Rev*: John Wiley & Sons, Ltd; 2015.
9. Huitema G, Willems Paul C, van Rhijn L, Kleijnen J, Shaffrey Christopher I: **Anterior versus posterior spinal correction and fusion for adolescent idiopathic scoliosis.** In *Cochrane Database Syst Rev*: John Wiley & Sons, Ltd; 2014.
10. Jadotte Yuri T, Santer M, Vakirlis E, Schwartz Robert A, Bauer A, Gundersen Daniel A, Mossman K, Lewith G: **Complementary and alternative medicine treatments for atopic eczema.** In *Cochrane Database Syst Rev*: John Wiley & Sons, Ltd; 2014.
11. Miller J, Gross A, Kay Theresa M, Graham N, Burnie Stephen J, Goldsmith Charles H, Brønfort G, Hoving Jan L, MacDermid J: **Manual therapy with exercise for neck pain.** In *Cochrane Database Syst Rev*: John Wiley & Sons, Ltd; 2014.
12. Monson Carlos A, Silva V, Andriolo Régis B, Kozasa Elisa H, Sabbag Cid Y, Paula Carlos Alberto d, Tweed John A, Fernandes Moça Trevisani V: **Complementary therapies for chronic plaque psoriasis.** In *Cochrane Database Syst Rev*: John Wiley & Sons, Ltd; 2014.
13. Parreira P, Heymans Martijn W, van Tulder Maurits W, Esmail R, Koes Bart W, Poquet N, Lin Chung-Wei C, Maher Christopher G: **Back schools for chronic non-specific low back pain.** In *Cochrane Database Syst Rev*: John Wiley & Sons, Ltd; 2015.
14. Plumbe L, Peters S, Bennett S, Vicenzino B, Coppieters Michel W: **Mirror therapy, graded motor imagery and virtual illusion for the management of chronic pain.** In *Cochrane Database Syst Rev*: John Wiley & Sons, Ltd; 2013.
15. Samuel S, David Kenny S, Gray Randolph J, Tharyan P: **Fusion versus conservative management for low-grade isthmic spondylolisthesis.** In *Cochrane Database Syst Rev*: John Wiley & Sons, Ltd; 2012.
16. Shin E-S, Lee S-H, Seo K-H: **Massage with or without aromatherapy for symptom relief in patients with cancer.** In *Cochrane Database Syst Rev*: John Wiley & Sons, Ltd; 2014.
17. Smart Keith M, Wand Benedict M, O'Connell Neil E: **Physiotherapy for pain and disability in adults with complex regional pain syndrome (CRPS) types I and II.** In *Cochrane Database Syst Rev*: John Wiley & Sons, Ltd; 2013.
18. Smidt N, Assendelft Willem JJ, Arola H, Malmivaara A, Green S, Buchbinder R, Bouter Lex M: **Physiotherapy and physiotherapeutical modalities for lateral epicondylitis.** In *Cochrane Database Syst Rev*: John Wiley & Sons, Ltd; 1999.
19. Theivendran K, Thakrar Raj R, Deshmukh Subodh C, Dwan K: **Closed reduction methods for acute anterior shoulder dislocation.** In *Cochrane Database Syst Rev*: John Wiley & Sons, Ltd; 2014.
20. Westby Marie D, Kennedy D, Jones D, Jones A, Doyle-Waters MM, Backman C: **Post-acute physiotherapy for primary total knee arthroplasty.** In *Cochrane Database Syst Rev*: John Wiley & Sons, Ltd; 2008.
21. Zaina F, Tomkins-Lane C, Carragee E, Negrini S: **Surgical versus non-surgical treatment for lumbar spinal stenosis.** In *Cochrane Database Syst Rev*: John Wiley & Sons, Ltd; 2012.
22. Zanon Márcia A, Porfírio Gustavo JM, Riera R: **Neurodevelopmental treatment approaches for children with cerebral palsy.** In *Cochrane Database Syst Rev*: John Wiley & Sons, Ltd; 2015.

From updated search:

1. Bremner M, Blake B, Stiles C: **The experiences of persons living with HIV who participate in mind-body and energy therapies: a systematic review protocol of qualitative evidence.** *JBI Database System Rev Implement Rep* 2015, **13:**41-49.

2. Gendron Louis M, Nyberg A, Maltais F, Lacasse Y: **Active mind-body movement therapies as an adjunct to or in comparison to pulmonary rehabilitation for people with chronic obstructive pulmonary disease.** In *Cochrane Database Syst Rev*: John Wiley & Sons, Ltd; 2016.
3. Hilde G, Gutke A, Slade Susan C, Stuge B: **Physical therapy interventions for pelvic girdle pain (PGP) after pregnancy.** In *Cochrane Database Syst Rev*: John Wiley & Sons, Ltd; 2016.
4. Haas M, Brønfort G, Evans Roni L, Leininger B, Schmitt J, Levin M, Westrom K, Goldsmith Charles H: **Spinal rehabilitative exercise or manual treatment for the prevention of cervicogenic headache in adults.** In *Cochrane Database Syst Rev*: John Wiley & Sons, Ltd; 2016.
5. Kim CG, Mun SJ, Kim KN, Shin BC, Kim NK, Lee DH, Lee JH: **Economic evaluation of manual therapy for musculoskeletal diseases: a protocol for a systematic review and narrative synthesis of evidence.** *BMJ Open* 2016, **6:**e010556.
6. Leininger B, Brønfort G, Haas M, Schmitt J, Evans Roni L, Levin M, Westrom K, Goldsmith Charles H: **Spinal rehabilitative exercise or manual treatment for the prevention of tension-type headache in adults.** In *Cochrane Database Syst Rev*: John Wiley & Sons, Ltd; 2016.
7. McClurg D, Pollock A, Campbell P, Hazelton C, Elders A, Hagen S, Hill David C: **Conservative interventions for urinary incontinence in women: an Overview of Cochrane systematic reviews.** In *Cochrane Database Syst Rev*: John Wiley & Sons, Ltd; 2016.
8. Rogers Jason A, Wilson A, Laslett Laura L, Winzenberg Tania M: **Physical interventions (orthoses, splints, exercise and manual therapy) for treating plantar heel pain.** In *Cochrane Database Syst Rev*: John Wiley & Sons, Ltd; 2016.

Withdrawn (6 records and no records*)

1. Assendelft WJ, Morton SC, Yu EI, Suttorp MJ, Shekelle PG: **WITHDRAWN: Spinal manipulative therapy for low-back pain.** In *Cochrane Database Syst Rev*, vol. 1, 2013/02/27 edition; 2013.
2. Gross AR, Aker PD, Goldsmith CH, Peloso P: **WITHDRAWN: Physical medicine modalities for mechanical neck disorders.** In *Cochrane Database Syst Rev*, 2007/07/20 edition; 1998.
3. Gross AR, Aker PD, Goldsmith CH, Peloso P: **Physical medicine modalities for mechanical neck disorders.** In *Cochrane Database Syst Rev*, 2000/05/05 edition; 2000.
4. Hoskins W, Pollard H: **Retraction: a descriptive study of a manual therapy intervention within a randomised controlled trial for hamstring and lower limb injury prevention.** *Chiropr Man Therap* 2011, **19:**24.
5. Verhagen AP, Karels C, Bierma-Zeinstra SM, Burdorf L, Feleus A, Dahaghin S, de Vet HC, Koes BW: **Ergonomic and physiotherapeutic interventions for treating work-related complaints of the arm, neck or shoulder in adults.** In *Cochrane Database Syst Rev*, 2006/07/21 edition; 2006.
6. Verhagen AP, Karels CC, Bierma-Zeinstra SM, Burdorf LL, Feleus A, Dahaghin SS, de Vet HC, Koes BW: **WITHDRAWN: Ergonomic and physiotherapeutic interventions for treating work-related complaints of the arm, neck or shoulder in adults.** In *Cochrane Database Syst Rev*, 2009/07/10 edition; 2009.

Not possible to retrieve (4 records and no records*)

1. Ecri: **Manipulation under anesthesia for low-back pain (Structured abstract).** In *Health Technology Assessment Database*. pp. 33; 2003:33.
2. Hayes, Inc: **Chiropractic treatment for low back pain (Structured abstract).** In *Health Technology Assessment Database*: HAYES, Inc; 2005.
3. Hayes, Inc: **Spinal manipulation under anesthesia for the treatment of pain (Structured abstract).** In *Health Technology Assessment Database*: HAYES, Inc; 2007.

4. Hayes, Inc: **Osteopathic Manipulative Treatment (OMT) for back pain (Structured abstract).** In *Health Technology Assessment Database*: HAYES, Inc; 2011.

An update exists (12 records and no records*)

1. Brantingham JW, Globe G, Pollard H, Hicks M, Korporaal C, Hoskins W: **Manipulative therapy for lower extremity conditions: expansion of literature review.** *J Manipulative Physiol Ther* 2009, **32:**53-71.
2. Bryans R, Descarreaux M, Duranleau M, Marcoux H, Potter B, Ruegg R, Shaw L, Watkin R, White E: **Evidence-based guidelines for the chiropractic treatment of adults with headache.** *J Manipulative Physiol Ther* 2011, **34:**274-289.
3. D'Sylva J, Miller J, Gross A, Burnie SJ, Goldsmith CH, Graham N, Haines T, Bronfort G, Hoving JL: **Manual therapy with or without physical medicine modalities for neck pain: a systematic review.** *Man Ther* 2010, **15:**415-433.
4. Furlan AD, Brosseau L, Imamura M, Irvin E: **Massage for low back pain.** In *Cochrane Database Syst Rev*, 2002/06/22 edition; 2002.
5. Glazener CM, Evans JH, Cheuk DK: **Complementary and miscellaneous interventions for nocturnal enuresis in children.** In *Cochrane Database Syst Rev*, 2005/04/23 edition; 2005.
6. Gross A, Miller J, D'Sylva J, Burnie SJ, Goldsmith CH, Graham N, Haines T, Bronfort G, Hoving JL: **Manipulation or mobilisation for neck pain.** In *Cochrane Database Syst Rev*, 2010/01/22 edition; 2010.
7. Gross A, Miller J, D'Sylva J, Burnie SJ, Goldsmith CH, Graham N, Haines T, Bronfort G, Hoving JL: **Manipulation or mobilisation for neck pain: a Cochrane Review.** *Man Ther* 2010, **15:**315-333.
8. Gross AR, Hoving JL, Haines TA, Goldsmith CH, Kay T, Aker P, Bronfort G: **A Cochrane review of manipulation and mobilization for mechanical neck disorders.** *Spine (Phila Pa 1976)* 2004, **29:**1541-1548.
9. Gross AR, Hoving JL, Haines TA, Goldsmith CH, Kay T, Aker P, Bronfort G: **Manipulation and mobilisation for mechanical neck disorders.** In *Cochrane Database Syst Rev*, 2004/02/20 edition; 2004.
10. Hondras MA, Linde K, Jones AP: **Manual therapy for asthma.** In *Cochrane Database Syst Rev*, 2000/05/05 edition; 2000.
11. Miller J, Gross A, D'Sylva J, Burnie SJ, Goldsmith CH, Graham N, Haines T, Bronfort G, Hoving JL: **Manual therapy and exercise for neck pain: a systematic review.** *Man Ther* 2010, **15:**334-354.
12. Yang M, Yuping Y, Yin X, Wang BY, Wu T, Liu GJ, Dong BR: **Chest physiotherapy for pneumonia in adults.** *Cochrane Database Syst Rev* 2010.

Not relevant for other reasons (6 records and 1 record*)

1. Barnes J, Abbot NC, Harkness EF, Ernst E: **Articles on complementary medicine in the mainstream medical literature: an investigation of MEDLINE, 1966 through 1996.** *Arch Intern Med* 1999, **159:**1721-1725.
2. Frass M, Strassl RP, Friehs H, Mullner M, Kundi M, Kaye AD: **Use and acceptance of complementary and alternative medicine among the general population and medical personnel: a systematic review.** *Ochsner J* 2012, **12:**45-56.

3. Haneline MT: **A review of the use of likelihood ratios in the chiropractic literature.** *J Chiropr Med* 2007, **6:**99-104.
4. Khorsan R, Coulter ID, Hawk C, Choate CG: **Measures in chiropractic research: choosing patient-based outcome assessments.** *J Manipulative Physiol Ther* 2008, **31:**355-375.
5. Philadelphia P: **Philadelphia Panel evidence-based clinical practice guidelines on selected rehabilitation interventions: overview and methodology.** *Phys Ther* 2001, **81:**1629-1640.
6. Wei X, Wang S, Li J, Gao J, Yu J, Feng M, Zhu L: **Complementary and Alternative Medicine for the Management of Cervical Radiculopathy: An Overview of Systematic Reviews.** *Evid Based Complement Alternat Med* 2015, **2015**.

From updated search:

1. Hayes, Inc: **Coflex Interlaminar Stabilization Device (Paradigm Spine LLC) for treatment of lumbar spinal stenosis (Structured abstract).** In *Health Technology Assessment Database*: HAYES, Inc; 2016.
