# Supplementary material for: The risk associated with spinal manipulation: an overview of reviews
Source: Syst Rev. 2017 Mar 24;6:64. doi: 10.1186/s13643-017-0458-y (PMC5366149; doi:10.1186/s13643-017-0458-y)
Supplement: Supplementary file 5 — Reference lists for the included reviews in the overview. (PDF 177 kb) [file 13643_2017_458_MOESM5_ESM.pdf]

Reference Lists for the Included Reviews in the Overview

From search conducted 8th of December 2015 (110 records)

1. Aker PD, Gross AR, Goldsmith CH, Peloso P: **Conservative management of mechanical neck pain: systematic overview and meta-analysis.** *BMJ* 1996, **313:**1291-1296.
2. Assendelft WJ, Bouter LM, Knipschild PG: **Complications of spinal manipulation: a comprehensive review of the literature.** *J Fam Pract* 1996, **42:**475-480.
3. Boudreau R, Argaez C: **Chiropractic interventions for acute or chronic lower back pain in adults: a review of the clinical and cost-effectiveness** In *Health Technology Assessment Database*: Canadian Agency for Drugs and Technologies in Health (CADTH); 2009.
4. Boudreau R, Spry C: **Treatment of hydromyelia in adults: a review of clinical effectiveness and guidelines.** In *Health Technology Assessment Database*: Canadian Agency for Drugs and Technologies in Health (CADTH); 2009.
5. Brantingham JW, Bonnefin D, Perle SM, Cassa TK, Globe G, Pribicevic M, Hicks M, Korporaal C: **Manipulative therapy for lower extremity conditions: update of a literature review.** *J Manipulative Physiol Ther* 2012, **35:**127-166.
6. Brantingham JW, Cassa TK, Bonnefin D, Jensen M, Globe G, Hicks M, Korporaal C: **Manipulative therapy for shoulder pain and disorders: expansion of a systematic review.** *J Manipulative Physiol Ther* 2011, **34:**314-346.
7. Brantingham JW, Cassa TK, Bonnefin D, Pribicevic M, Robb A, Pollard H, Tong V, Korporaal C: **Manipulative and multimodal therapy for upper extremity and temporomandibular disorders: a systematic review.** *J Manipulative Physiol Ther* 2013, **36:**143-201.
8. Bronfort G, Assendelft WJ, Evans R, Haas M, Bouter L: **Efficacy of spinal manipulation for chronic headache: a systematic review.** *J Manipulative Physiol Ther* 2001, **24:**457-466.
9. Brown A, Angus D, Chen S, Tang Z, Milne S, Pfaff J, Li H, Mensinkai S: **Costs and outcomes of chiropractic treatment for low back pain** In *Health Technology Assessment Database*. pp. 88; 2005:88.
10. Brurberg KG, Myrhaug HT, Reinar LM: **Diagnostics and treatment of infant suspected with kinematic imbalance due to suboccipital strain (KISS).** In *Rapport fra Kunnskapssenteret nr 17−2009*: The Norwegian Knowledge Centre for the Health Services (NOKC); 2009.
11. Bryans R, Decina P, Descarreaux M, Duranleau M, Marcoux H, Potter B, Ruegg RP, Shaw L, Watkin R, White E: **Evidence-based guidelines for the chiropractic treatment of adults with neck pain.** *J Manipulative Physiol Ther* 2014, **37:**42-63.
12. Brønfort G, Nilsson N, Haas M, Evans R, Goldsmith CH, Assendelft WJ, Bouter LM: **Non-invasive physical treatments for chronic/recurrent headache.** In *Cochrane Database Syst Rev*, 2004/07/22 edition; 2004.
13. Carlesso LC, Gross AR, Santaguida PL, Burnie S, Voth S, Sadi J: **Adverse events associated with the use of cervical manipulation and mobilization for the treatment of neck pain in adults: a systematic review.** *Man Ther* 2010, **15:**434-444.
14. Carnes D, Mars TS, Mullinger B, Froud R, Underwood M: **Adverse events and manual therapy: a systematic review.** *Man Ther* 2010, **15:**355-363.
15. Chou R, Huffman L: **Evaluation and management of low back pain.** Glenview, Illinois: American Pain Society; 2007.
16. Chou R, Huffman LH: **Nonpharmacologic therapies for acute and chronic low back pain: a review of the evidence for an American Pain Society/American College of Physicians clinical practice guideline** *Ann Intern Med* 2007, **147:**492-504.
17. Cicchitti L, Martelli M, Cerritelli F: **Chronic inflammatory disease and osteopathy: a systematic review.** *PLoS One* 2015, **10**.

18. Clar C, Tsertsvadze A, Court R, Hundt GL, Clarke A, Sutcliffe P: **Clinical effectiveness of manual therapy for the management of musculoskeletal and non-musculoskeletal conditions: systematic review and update of UK evidence report.** *Chiropr Man Therap* 2014, **22:**12.
19. Close C, Sinclair M, Liddle SD, Madden E, McCullough JE, Hughes C: **A systematic review investigating the effectiveness of Complementary and Alternative Medicine (CAM) for the management of low back and/or pelvic pain (LBPP) in pregnancy.** *J Adv Nurs* 2014, **70:**1702-1716.
20. Coulter ID, Hurwitz E, Adams AH, Meeker W, Hansen DT, Mootz R, Aker P, Genovese B, Shekelle PG: *The Appropriateness of Manipulation and Mobilization of the Cervical Spine.* Santa Monica, CA: RAND Corporation; 1996.
21. Cross KM, Kuenze C, Grindstaff TL, Hertel J: **Thoracic spine thrust manipulation improves pain, range of motion, and self-reported function in patients with mechanical neck pain: a systematic review.** *J Orthop Sports Phys Ther* 2011, **41:**633-642.
22. Dabbs V, Lauretti WJ: **A risk assessment of cervical manipulation vs. NSAIDs for the treatment of neck pain.** *J Manipulative Physiol Ther* 1995, **18:**530-536.
23. Dobson D, Lucassen Peter LBJ, Miller Joyce J, Vlieger Arine M, Prescott P, Lewith G: **Manipulative therapies for infantile colic.** In *Cochrane Database Syst Rev*: John Wiley & Sons, Ltd; 2012.
24. Ernst E: **Does spinal manipulation have specific treatment effects?** *Fam Pract* 2000, **17:**554-556.
25. Ernst E: **Prospective investigations into the safety of spinal manipulation.** *J Pain Symptom Manage* 2001, **21:**238-242.
26. Ernst E: **Adverse effects of unconventional therapies in the elderly: A systematic review of the recent literature.** *J Am Aging Assoc* 2002, **25:**11-20.
27. Ernst E: **Manipulation of the cervical spine: a systematic review of case reports of serious adverse events, 1995-2001.** *Med J Aust* 2002, **176:**376-380.
28. Ernst E: **Serious adverse effects of unconventional therapies for children and adolescents: a systematic review of recent evidence.** *Eur J Pediatr* 2003, **162:**72-80.
29. Ernst E: **Cerebrovascular Complications Associated with Spinal Manipulation.** *Phys Ther Rev* 2004, **9:**5-15.
30. Ernst E: **Ophthalmological adverse effects of (chiropractic) upper spinal manipulation: evidence from recent case reports.** *Acta Ophthalmol Scand* 2005, **83:**581-585.
31. Ernst E: **Adverse effects of spinal manipulation: a systematic review.** *J R Soc Med* 2007, **100:**330-338.
32. Ernst E: **Deaths after chiropractic: a review of published cases.** *Int J Clin Pract* 2010, **64:**1162-1165.
33. Ernst E, Harkness E: **Spinal manipulation: a systematic review of sham-controlled, double-blind, randomized clinical trials.** *J Pain Symptom Manage* 2001, **22:**879-889.
34. Fabio RP: **Manipulation of the cervical spine: risks and benefits** *Phys Ther* 1999, **79:**50-65.
35. Franke H, Franke JD, Fryer G: **Osteopathic manipulative treatment for nonspecific low back pain: a systematic review and meta-analysis.** *BMC Musculoskelet Disord* 2014, **15:**286.
36. Furlan AD, Yazdi F, Tsertsvadze A, Gross A, Van Tulder M, Santaguida L, Cherkin D, Gagnier J, Ammendolia C, Ansari MT, et al: **Complementary and alternative therapies for back pain II.** *Evid Rep Technol Assess (Full Rep)* 2010:1-764.
37. Furlan AD, Yazdi F, Tsertsvadze A, Gross A, Van Tulder M, Santaguida L, Gagnier J, Ammendolia C, Dryden T, Doucette S, et al: **A systematic review and meta-analysis of efficacy, cost-effectiveness, and safety of selected complementary and alternative medicine for neck and low-back pain.** *Evid Based Complement Alternat Med* 2012, **2012**.
38. Gemmell H, Miller P: **Comparative effectiveness of manipulation, mobilisation and the activator instrument in treatment of non-specific neck pain: a systematic review.** *Chiropr Osteopat* 2006, **14:**7.
39. Gerritsen AA, Krom MC, Struijs MA, Scholten RJ, Vet HC, Bouter LM: **Conservative treatment options for carpal tunnel syndrome: a systematic review of randomised controlled trials** *J Neurol* 2002, **249:**272-280.

40. Gleberzon BJ, Arts J, Mei A, McManus EL: **The use of spinal manipulative therapy for pediatric health conditions: a systematic review of the literature.** *J Can Chiropr Assoc* 2012, **56:**128-141.
41. Gouveia LO, Castanho P, Ferreira JJ: **Safety of chiropractic interventions: a systematic review.** *Spine (Phila Pa 1976)* 2009, **34:**E405-413.
42. Gross A, Langevin P, Burnie SJ, Bedard-Brochu MS, Empey B, Dugas E, Faber-Dobrescu M, Andres C, Graham N, Goldsmith CH, et al: **Manipulation and mobilisation for neck pain contrasted against an inactive control or another active treatment.** In *Cochrane Database Syst Rev*, vol. 9, 2015/09/24 edition; 2015.
43. Gross AR, Goldsmith C, Hoving JL, Haines T, Peloso P, Aker P, Santaguida P, Myers C: **Conservative management of mechanical neck disorders: a systematic review.** *J Rheumatol* 2007, **34:**1083-1102.
44. Gross AR, Kay T, Hondras M, Goldsmith C, Haines T, Peloso P, Kennedy C, Hoving J: **Manual therapy for mechanical neck disorders: a systematic review.** *Man Ther* 2002, **7:**131-149.
45. Gross AR, Kay TM, Kennedy C, Gasner D, Hurley L, Yardley K, Hendry L, McLaughlin L: **Clinical practice guideline on the use of manipulation or mobilization in the treatment of adults with mechanical neck disorders.** *Man Ther* 2002, **7:**193-205.
46. Hahne AJ, Ford JJ, McMeeken JM: **Conservative management of lumbar disc herniation with associated radiculopathy: a systematic review.** *Spine (Phila Pa 1976)* 2010, **35:**E488-504.
47. Haldeman S, Kohlbeck FJ, McGregor M: **Risk factors and precipitating neck movements causing vertebrobasilar artery dissection after cervical trauma and spinal manipulation.** *Spine (Phila Pa 1976)* 1999, **24:**785-794.
48. Hawk C, Khorsan R, Lisi AJ, Ferrance RJ, Evans MW: **Chiropractic care for nonmusculoskeletal conditions: a systematic review with implications for whole systems research.** *J Altern Complement Med* 2007, **13:**491-512.
49. Haynes MJ, Vincent K, Fischhoff C, Bremner AP, Lanlo O, Hankey GJ: **Assessing the risk of stroke from neck manipulation: a systematic review.** *Int J Clin Pract* 2012, **66:**940-947.
50. Hebert JJ, Stomski NJ, French SD, Rubinstein SM: **Serious Adverse Events and Spinal Manipulative Therapy of the Low Back Region: A Systematic Review of Cases.** *J Manipulative Physiol Ther* 2013.
51. Hondras Maria A, Linde K, Jones Arthur P: **Manual therapy for asthma.** In *Cochrane Database Syst Rev*: John Wiley & Sons, Ltd; 2005.
52. Huang T, Shu X, Huang YS, Cheuk DK: **Complementary and miscellaneous interventions for nocturnal enuresis in children.** In *Cochrane Database Syst Rev*, 2011/12/14 edition; 2011.
53. Huisman PA, Speksnijder CM, de Wijer A: **The effect of thoracic spine manipulation on pain and disability in patients with non-specific neck pain: a systematic review.** *Disabil Rehabil* 2013, **35:**1677-1685.
54. Hunt KJ, Hung SK, Boddy K, Ernst E: **Chiropractic manipulation for carpal tunnel syndrome: a systematic review.** *Hand Therapy* 2009, **14:**89-94.
55. Hurwitz EL, Aker PD, Adams AH, Meeker WC, Shekelle PG: **Manipulation and mobilization of the cervical spine. A systematic review of the literature.** *Spine (Phila Pa 1976)* 1996, **21:**1746-1759; discussion 1759-1760.
56. Kaminskyj A, Frazier M, Johnstone K, Gleberzon BJ: **Chiropractic care for patients with asthma: A systematic review of the literature.** *J Can Chiropr Assoc* 2010, **54:**24-32.
57. Khorsan R, Hawk C, Lisi AJ, Kizhakkeveettil A: **Manipulative therapy for pregnancy and related conditions: a systematic review.** *Obstet Gynecol Surv* 2009, **64:**416-427.
58. Kizhakkeveettil A, Rose K, Kadar GE: **Integrative therapies for low back pain that include complementary and alternative medicine care: a systematic review.** *Glob Adv Health Med* 2014, **3:**49-64.
59. Kuczynski JJ, Schwieterman B, Columber K, Knupp D, Shaub L, Cook CE: **Effectiveness of physical therapist administered spinal manipulation for the treatment of low back pain: a systematic review of the literature.** *Int J Sports Phys Ther* 2012, **7:**647-662.

60. Lenssinck ML, Damen L, Verhagen AP, Berger MY, Passchier J, Koes BW: **The effectiveness of physiotherapy and manipulation in patients with tension-type headache: a systematic review.** *Pain* 2004, **112:**381-388.
61. Liddle Sarah D, Pennick V: **Interventions for preventing and treating low-back and pelvic pain during pregnancy.** In *Cochrane Database Syst Rev*: John Wiley & Sons, Ltd; 2015.
62. Lin JH, Chiu TT, Hu J: **Chinese manipulation for mechanical neck pain: a systematic review.** *Clin Rehabil* 2012, **26:**963-973.
63. Lisi AJ, Holmes EJ, Ammendolia C: **High-velocity low-amplitude spinal manipulation for symptomatic lumbar disk disease: a systematic review of the literature.** *J Manipulative Physiol Ther* 2005, **28:**429-442.
64. Luijsterburg PA, Verhagen AP, Ostelo RW, Os TA, Peul WC, Koes BW: **Effectiveness of conservative treatments for the lumbosacral radicular syndrome: a systematic review** *Eur Spine J* 2007, **16:**881-899.
65. Lystad RP, Bell G, Bonnevie-Svendsen M, Carter CV: **Manual therapy with and without vestibular rehabilitation for cervicogenic dizziness: a systematic review.** *Chiropr Man Therap* 2011, **19:**21.
66. Magee DJ, Oborn-Barrett E, Turner S, Fenning N: **A systematic overview of the effectiveness of physical therapy intervention on soft tissue neck injury following trauma** *Physiotherapy Canada* 2000, **52:**111-130.
67. Miley ML, Wellik KE, Wingerchuk DM, Demaerschalk BM: **Does cervical manipulative therapy cause vertebral artery dissection and stroke?** *Neurologist* 2008, **14:**66-73.
68. Oduneye F: **Spinal manipulation for chronic neck pain** In *Health Technology Assessment Database*. pp. 10; 2004:10.
69. Oliphant D: **Safety of spinal manipulation in the treatment of lumbar disk herniations: a systematic review and risk assessment.** *J Manipulative Physiol Ther* 2004, **27:**197-210.
70. Page Matthew J, Green S, Kramer S, Johnston Renea V, McBain B, Chau M, Buchbinder R: **Manual therapy and exercise for adhesive capsulitis (frozen shoulder).** In *Cochrane Database Syst Rev*: John Wiley & Sons, Ltd; 2014.
71. Parkinson L, Sibbritt D, Bolton P, van Rotterdam J, Villadsen I: **Well-being outcomes of chiropractic intervention for lower back pain: a systematic review.** *Clin Rheumatol* 2013, **32:**167-180.
72. Posadzki P, Ernst E: **Osteopathy for musculoskeletal pain patients: a systematic review of randomized controlled trials.** *Clin Rheumatol* 2011, **30:**285-291.
73. Posadzki P, Ernst E: **Spinal manipulations for cervicogenic headaches: a systematic review of randomized clinical trials.** *Headache* 2011, **51:**1132-1139.
74. Posadzki P, Ernst E: **Spinal manipulations for the treatment of migraine: a systematic review of randomized clinical trials.** *Cephalalgia* 2011, **31:**964-970.
75. Posadzki P, Ernst E: **Systematic reviews of spinal manipulations for headaches: an attempt to clear up the confusion.** *Headache* 2011, **51:**1419-1425.
76. Posadzki P, Ernst E: **Spinal manipulations for tension-type headaches: a systematic review of randomized controlled trials.** *Complement Ther Med* 2012, **20:**232-239.
77. Posadzki P, Lee MS, Ernst E: **Osteopathic manipulative treatment for pediatric conditions: a systematic review.** *Pediatrics* 2013, **132:**140-152.
78. Proctor M, Hing W, Johnson Trina C, Murphy Patricia A, Brown J: **Spinal manipulation for dysmenorrhoea.** In *Cochrane Database Syst Rev*: John Wiley & Sons, Ltd; 2006.
79. Puentedura EJ, March J, Anders J, Perez A, Landers MR, Wallmann HW, Cleland JA: **Safety of cervical spine manipulation: are adverse events preventable and are manipulations being performed appropriately? A review of 134 case reports.** *J Man Manip Ther* 2012, **20:**66-74.
80. Puentedura EJ, O'Grady WH: **Safety of thrust joint manipulation in the thoracic spine: a systematic review.** *J Man Manip Ther* 2015, **23:**154-161.
81. Reiman MP, Harris JY, Cleland JA: **Manual therapy interventions for patients with lumbar spinal stenosis: a systematic review** *New Zealand Journal of Physiotherapy* 2009, **37:**17-28.

82. Rubinstein Sidney M, Terwee Caroline B, Assendelft Willem JJ, de Boer Michiel R, van Tulder Maurits W: **Spinal manipulative therapy for acute low-back pain.** In *Cochrane Database Syst Rev*: John Wiley & Sons, Ltd; 2012.
83. Rubinstein Sidney M, van Middelkoop M, Assendelft Willem JJ, de Boer Michiel R, van Tulder Maurits W: **Spinal manipulative therapy for chronic low-back pain.** In *Cochrane Database Syst Rev*: John Wiley & Sons, Ltd; 2011.
84. Rubinstein SM, Peerdeman SM, van Tulder MW, Riphagen I, Haldeman S: **A systematic review of the risk factors for cervical artery dissection.** *Stroke* 2005, **36:**1575-1580.
85. Rubinstein SM, Terwee CB, Assendelft WJ, de Boer MR, van Tulder MW: **Spinal manipulative therapy for acute low back pain: an update of the cochrane review.** *Spine (Phila Pa 1976)* 2013, **38:**E158-177.
86. Rubinstein SM, van Middelkoop M, Assendelft WJ, de Boer MR, van Tulder MW: **Spinal manipulative therapy for chronic low-back pain: an update of a Cochrane review.** *Spine (Phila Pa 1976)* 2011, **36:**E825-846.
87. Scholten-Peeters GG, Thoomes E, Konings S, Beijer M, Verkerk K, Koes BW, Verhagen AP: **Is manipulative therapy more effective than sham manipulation in adults : a systematic review and meta-analysis.** *Chiropr Man Therap* 2013, **21:**34.
88. Schroeder J, Kaplan L, Fischer DJ, Skelly AC: **The outcomes of manipulation or mobilization therapy compared with physical therapy or exercise for neck pain: a systematic review.** *Evid Based Spine Care J* 2013, **4:**30-41.
89. Shekelle PG, Adams AH, Chassin MR, Hurwitz EL, Brook RH: **Spinal manipulation for low-back pain.** *Ann Intern Med* 1992, **117:**590-598.
90. Shin BC, Lee MS, Park TY, Ernst E: **Serious adverse events after spinal manipulation: A systematic review of the Korean literature.** *Focus Altern Complement Ther* 2010, **15:**198-201.
91. Snelling NJ: **Spinal manipulation in patients with disc herniation: A critical review of risk and benefit.** *Int J Osteopath Med* 2006, **9:**77-84.
92. Southerst D, Marchand AA, Cote P, Shearer HM, Wong JJ, Varatharajan S, Randhawa K, Sutton D, Yu H, Gross DP, et al: **The Effectiveness of Noninvasive Interventions for Musculoskeletal Thoracic Spine and Chest Wall Pain: A Systematic Review by the Ontario Protocol for Traffic Injury Management (OPTIMa) Collaboration.** *J Manipulative Physiol Ther* 2015, **38:**521-531.
93. Stevinson C, Ernst E: **Risks associated with spinal manipulation.** *Am J Med* 2002, **112:**566-571.
94. Stuber KJ, Smith DL: **Chiropractic treatment of pregnancy-related low back pain: a systematic review of the evidence.** *J Manipulative Physiol Ther* 2008, **31:**447-454.
95. Stuber KJ, Wynd S, Weis CA: **Adverse events from spinal manipulation in the pregnant and postpartum periods: a critical review of the literature.** *Chiropr Man Therap* 2012, **20:**8.
96. Sutton DA, Cote P, Wong JJ, Varatharajan S, Randhawa KA, Yu H, Southerst D, Shearer HM, van der Velde GM, Nordin MC, et al: **Is multimodal care effective for the management of patients with whiplash-associated disorders or neck pain and associated disorders? A systematic review by the Ontario Protocol for Traffic Injury Management (OPTIMa) Collaboration.** *Spine J* 2014.
97. Todd AJ, Carroll MT, Robinson A, Mitchell EK: **Adverse Events Due to Chiropractic and Other Manual Therapies for Infants and Children: A Review of the Literature.** *J Manipulative Physiol Ther* 2014.
98. Tuchin P: **A systematic literature review of intracranial hypotension following chiropractic.** *Int J Clin Pract* 2014, **68:**396-402.
99. Vernon H, Humphreys BK: **Manual therapy for neck pain: an overview of randomized clinical trials and systematic reviews.** *Eura Medicophys* 2007, **43:**91-118.
100. Vernon H, Humphreys BK: **Chronic mechanical neck pain in adults treated by manual therapy: a systematic review of change scores in randomized controlled trials of a single session.** *J Man Manip Ther* 2008, **16:**E42-52.

101. Vernon H, Humphreys K, Hagino C: **Chronic mechanical neck pain in adults treated by manual therapy: a systematic review of change scores in randomized clinical trials.** *J Manipulative Physiol Ther* 2007, **30:**215-227.
102. Vernon H, McDermaid CS, Hagino C: **Systematic review of randomized clinical trials of complementary/alternative therapies in the treatment of tension-type and cervicogenic headache.** *Complement Ther Med* 1999, **7:**142-155.
103. Walker BF, French SD, Grant W, Green S: **Combined chiropractic interventions for low-back pain.** In *Cochrane Database Syst Rev*, 2010/04/16 edition; 2010.
104. Walker BF, French SD, Grant W, Green S: **A Cochrane review of combined chiropractic interventions for low-back pain.** *Spine (Phila Pa 1976)* 2011, **36:**230-242.
105. Wynd S, Westaway M, Vohra S, Kawchuk G: **The quality of reports on cervical arterial dissection following cervical spinal manipulation.** *PLoS One* 2013, **8**.
106. Yang M, Yan Y, Yin X, Wang BY, Wu T, Liu GJ, Dong BR: **Chest physiotherapy for pneumonia in adults.** In *Cochrane Database Syst Rev*, vol. 2, 2013/03/02 edition; 2013.
107. Yin P, Gao N, Wu J, Litscher G, Xu S: **Adverse events of massage therapy in pain-related conditions: a systematic review.** *Evid Based Complement Alternat Med* 2014, **2014**.
108. Young JL, Walker D, Snyder S, Daly K: **Thoracic manipulation versus mobilization in patients with mechanical neck pain: a systematic review.** *J Man Manip Ther* 2014, **22:**141-153.
109. Yuan QL, Guo TM, Liu L, Sun F, Zhang YG: **Traditional Chinese medicine for neck pain and low back pain: a systematic review and meta-analysis.** *PLoS One* 2015, **10**.
110. Zhu L, Wei X, Wang S: **Does cervical spine manipulation reduce pain in people with degenerative cervical radiculopathy? A systematic review of the evidence, and a meta-analysis.** *Clin Rehabil* 2015.

From updated search conducted 10th of January 2017 (26 records)

1. Andronis L, Kinghorn P, Qiao S, Whitehurst DG, Durrell S, McLeod H: **Cost-Effectiveness of Non-Invasive and Non-Pharmacological Interventions for Low Back Pain: a Systematic Literature Review.** *Appl Health Econ Health Policy* 2016.
2. Blanchette MA, Stochkendahl MJ, Borges Da Silva R, Boruff J, Harrison P, Bussieres A: **Effectiveness and Economic Evaluation of Chiropractic Care for the Treatment of Low Back Pain: A Systematic Review of Pragmatic Studies.** *PLoS One* 2016, **11:**e0160037.
3. Cerritelli F, Ruffini N, Lacorte E, Vanacore N: **Osteopathic manipulative treatment in neurological diseases: Systematic review of the literature.** *J Neurol Sci* 2016, **369:**333-341.
4. Chou R, Deyo R, Friedly J, Skelly A, Hashimoto R, Weimer M, Fu R, Dana T, Kraegel P, Griffin J, et al: **Noninvasive treatments for low back pain (Structured abstract).** In *Health Technology Assessment Database*: Agency for Healthcare Research and Quality (AHRQ); 2016.
5. Chou R, Deyo R, Friedly J, Skelly A, Hashimoto R, Weimer M, Fu R, Dana T, Kraegel P, Griffin J, et al: **AHRQ Comparative Effectiveness Reviews.** In *Noninvasive Treatments for Low Back Pain.* Rockville (MD): Agency for Healthcare Research and Quality (US); 2016
6. Church EW, Sieg EP, Zalatimo O, Hussain NS, Glantz M, Harbaugh RE: **Systematic Review and Meta-analysis of Chiropractic Care and Cervical Artery Dissection: No Evidence for Causation.** *Cureus* 2016, **8:**e498.
7. Damgaard P, Bartels EM, Ris I, Christensen R, Juul-Kristensen B: **Evidence of Physiotherapy Interventions for Patients with Chronic Neck Pain: A Systematic Review of Randomised Controlled Trials.** *ISRN Pain* 2013, **2013:**567175.

8. Franke H, Franke JD, Fryer G: **Osteopathic manipulative treatment for chronic nonspecific neck pain: A systematic review and meta-analysis.** *Int J Osteopath Med* 2015, **18:**255-267.
9. Globe G, Farabaugh RJ, Hawk C, Morris CE, Baker G, Whalen WM, Walters S, Kaeser M, Dehen M, Augat T: **Clinical Practice Guideline: Chiropractic Care for Low Back Pain.** *J Manipulative Physiol Ther* 2016, **39:**1-22.
10. Hall H, Cramer H, Sundberg T, Ward L, Adams J, Moore C, Sibbritt D, Lauche R: **The effectiveness of complementary manual therapies for pregnancy-related back and pelvic pain: A systematic review with meta-analysis.** *Medicine (Baltimore)* 2016, **95:**e4723.
11. Michiels S, Naessens S, Van de Heyning P, Braem M, Visscher CM, Gilles A, De Hertogh W: **The Effect of Physical Therapy Treatment in Patients with Subjective Tinnitus: A Systematic Review.** *Front Neurosci* 2016, **10:**545.
12. Ng JY, Liang L, Gagliardi AR: **The quantity and quality of complementary and alternative medicine clinical practice guidelines on herbal medicines, acupuncture and spinal manipulation: systematic review and assessment using AGREE II.** *BMC Complement Altern Med* 2016, **16:**425.
13. Page Matthew J, Green S, McBain B, Surace Stephen J, Deitch J, Lyttle N, Mrocki Marshall A, Buchbinder R: **Manual therapy and exercise for rotator cuff disease.** In *Cochrane Database Syst Rev*: John Wiley & Sons, Ltd; 2016.
14. Posadzki P, Albedah AMN, Khalil MMK, Alqaed MS, Lee MS, Ernst E, Car J: **Complementary and alternative medicine for the prevention and treatment of migraine headache: An overview of systematic reviews.** *Focus Altern Complement Ther* 2015, **20:**58-73.
15. Puhl AA, Reinhart CJ, Doan JB, Vernon H: **The quality of placebos used in randomized, controlled trials of lumbar and pelvic joint thrust manipulation-a systematic review.** *Spine J* 2016.
16. Rothberg S, Friedman BW: **Complementary therapies in addition to medication for patients with nonchronic, nonradicular low back pain: a systematic review.** *Am J Emerg Med* 2017, **35:**55-61.
17. Ruddock JK, Sallis H, Ness A, Perry RE: **Spinal Manipulation Vs Sham Manipulation for Nonspecific Low Back Pain: A Systematic Review and Meta-analysis.** *J Chiropr Med* 2016, **15:**165-183.
18. Ruffini N, D'Alessandro G, Cardinali L, Frondaroli F, Cerritelli F: **Osteopathic manipulative treatment in gynecology and obstetrics: A systematic review.** *Complement Ther Med* 2016, **26:**72-78.
19. Stainsby BE, Clarke MC, Egonia JR: **Learning spinal manipulation: A best-evidence synthesis of teaching methods.** *J Chiropr Educ* 2016, **30:**138-151.
20. Steel A, Sundberg T, Reid R, Ward L, Bishop FL, Leach M, Cramer H, Wardle J, Adams J: **Osteopathic manipulative treatment: A systematic review and critical appraisal of comparative effectiveness and health economics research.** *Man Ther* 2016.
21. Thoomes EJ: **Effectiveness of manual therapy for cervical radiculopathy, a review.** *Chiropr Man Therap* 2016, **24:**45.
22. Varatharajan S, Ferguson B, Chrobak K, Shergill Y, Cote P, Wong JJ, Yu H, Shearer HM, Southerst D, Sutton D, et al: **Are non-invasive interventions effective for the management of headaches associated with neck pain? An update of the Bone and Joint Decade Task Force on Neck Pain and Its Associated Disorders by the Ontario Protocol for Traffic Injury Management (OPTIMa) Collaboration.** *Eur Spine J* 2016, **25:**1971-1999.
23. Wearing J, Beaumont S, Forbes D, Brown B, Engel R: **The Use of Spinal Manipulative Therapy in the Management of Chronic Obstructive Pulmonary Disease: A Systematic Review.** *J Altern Complement Med* 2016, **22:**108-114.
24. Wong JJ, Cote P, Sutton DA, Randhawa K, Yu H, Varatharajan S, Goldgrub R, Nordin M, Gross DP, Shearer HM, et al: **Clinical practice guidelines for the noninvasive management of low back pain: A systematic review by the Ontario Protocol for Traffic Injury Management (OPTIMa) Collaboration.** *Eur J Pain* 2017, **21:**201-216.
25. Wong JJ, Shearer HM, Mior S, Jacobs C, Cote P, Randhawa K, Yu H, Southerst D, Varatharajan S, Sutton D, et al: **Are manual therapies, passive physical modalities, or acupuncture effective for**

the management of patients with whiplash-associated disorders or neck pain and associated disorders? An update of the Bone and Joint Decade Task Force on Neck Pain and Its Associated Disorders by the OPTIMa collaboration. *Spine J* 2016, **16:**1598-1630.

26. Yao M, Sun YL, Dun RL, Lan TY, Li JL, Lee HJ, Haraguchi N, Wang YJ, Cui XJ: **Is manipulative therapy clinically necessary for relief of neck pain? A systematic review and meta-analysis.** *Chin J Integr Med* 2016.
