# Supplementary material for: The risk associated with spinal manipulation: an overview of reviews
Source: Syst Rev. 2017 Mar 24;6:64. doi: 10.1186/s13643-017-0458-y (PMC5366149; doi:10.1186/s13643-017-0458-y)
Supplement: Supplementary file 6 — Table showing further study characteristics including conclusions extracted from each reviews. (PDF 416 kb) [file 13643_2017_458_MOESM6_ESM.pdf]

Study Characteristics

Summary of findings for spinal manipulative therapy

Author (year) ref	Included studies on SMT (pts. in total receiving SMT)	Population receiving SMT	Interventions including SMT	AEs reported* associated with SMT)	Conclusion on AEs from SMT (quote)	Quality of the evidence for AEs (tool)
Blanchette, M. A. et al. (2016)	4 RCTs<br>(460 pts in total)	Pts with non-specific low back pain	SMT	NA<br><br>No SAEs	"The rate of adverse effects did not differ significantly between the two treatment groups (Table 4). These were rare (2% and 6% for chiropractic and exercise therapy care respectively), transient in nature, and necessitated minimal or no change in activity level. (…) Adverse events were only reported in the Cherkin study [51], and no serious adverse effects were recorded in either of the treatment groups (Table 4). (…) Moderate evidence suggests that chiropractic care for LBP appears to be equally effective as physical therapy. Limited evidence suggests the same conclusion when chiropractic care is compared to exercise therapy and medical care although no firm conclusion can be reached at this time. No serious adverse events were reported for any type of care. Our review was also unable to clarify whether chiropractic or medical care is more cost-effective. Given the limited available evidence, the decision to seek or to refer patients for chiropractic care should be based on patient preference and values. Future studies are likely to have an important impact on our estimates as these were based on only a few admissible studies."	Not reported
Cerritelli, F. et al. (2016)	2 RCTs<br>(31 pts in total)	Pts tension-type headache	OMT (including HVLA, i.e. may include SMT)	None	"Only 40% (4/10) of the included studies reported data on adverse events. No specific adverse events were recorded among the 296 patients included in the studies. (…) Another aspect to be considered is the reporting of adverse events. Only one third of the included trials mentioned adverse events, stating that patients did not report any adverse event. However, whether the reported adverse events were related to the study group, to the control group or both, was not always clear. Moreover, the number and quality of reported adverse events seems to be lower than the in all other nonpharmacological, non-interventional trials. In fact, none of the included trials reported any type of adverse events (neither mild nor severe), which is in contrast to other non-pharmacological approaches, thus suggesting possibly skewed data. (…) Results showed that studies on the efficacy and/or effectiveness of OMT treatments are scarce, heterogeneous, and of low methodological quality. Further studies should be conducted including a more pragmatic methodology, an exhaustive description of all investigated and concurrent interventions, and a systematic report of adverse events, so as to obtain robust and generalizable results."<br>*(Two of the trials were on SMT and one of these reported on AEs – no AEs was reported)*	Not reported
Chou, R. et al. (2016)	2 SRs, 16 RCTs<br>(>1100 pts in total)	Pts with low-back pain	SMT	Muscle soreness, stiffness, and/or transient increase in pain<br><br>No SAEs	"As in the prior APS/ACP review, we found that harms were poorly reported in trials of SMT. For chronic low back pain, ~two-thirds of trials did not report adverse events.438 When reported, adverse events in patients undergoing SMT were limited to muscle soreness, stiffness, and/or transient increase in pain. None of the studies reported any serious complications in either the experimental or control group."	"Low" (approach described in the AHRQ Methods Guide 19)

Author (year) [ref]	Included studies on SMT (pts. in total receiving SMT)	Population receiving SMT	Interventions including SMT	AEs reported* associated with SMT)	Conclusion on AEs from SMT (quote)	Quality of the evidence for AEs (tool)
Church, E. W. et al. (2016)	6 CCs (5934 pts in total)	Pts in studies examining carotid or vertebrobasilar artery dissection and recent chiropractic neck manipulation	Chiropractic care (including SMT)	NA<br><br>**SAEs**: Carotid artery dissection, vertebrobasilar artery dissection	"The quality of the published literature on the relationship between chiropractic manipulation and CAD is very low. Our analysis shows a small association between chiropractic neck manipulation and cervical artery dissection. This relationship may be explained by the high risk of bias and confounding in the available studies, and in particular by the known association of neck pain with CAD and with chiropractic manipulation. There is no convincing evidence to support a causal link between chiropractic manipulation and CAD. Belief in a causal link may have significant negative consequences such as numerous episodes of litigation."	"Very low" (GRADE)
Hall, H. et al. (2016)	4 RCTs (249 pts in total)	Pregnant women	OMT and SMT	Early contractions<br><br>No SAEs	"Only 3 studies reported safety, but no meta-analysis could be conducted. Although 1 study reported no health problems during the massages,[37] a case of early contractions in the control group was reported in another study.[47] A third study stated that the massage group had fewer obstetric complications, their newborns had fewer postnatal complications, less premature births and infants required less ventilatory assistance.[44] (…) There is currently limited evidence to support the use of manual therapies including massage and osteopathic manipulative treatment as an option for managing LBP and PGP during pregnancy. Current research is associated with a risk of publication and methodological biases, and lack of robust control comparisons. Further high-quality research is needed to determine causal effects, the influence of the therapist on the perceived effectiveness of treatments, and adequate dose-response of manual therapies on LBP and PGP outcomes during pregnancy."<br>*(One of the trials "[47]" reporting safety was on OMT i.e. possibly including SMT. Note they refer to the control group)*	Not reported
Page Matthew, J. et al. (2016)	4 RCTs (117 pts in total)	Pts with rotator cuff disease	SMT (mostly thoracic SMT)	None	"Based upon high quality data from this trial, there was no clinically important benefit of manual therapy and exercise over placebo. Adverse events were relatively more frequent with manual therapy and exercise but mild in nature (short-term pain following treatment)."<br>*(Of the trials on SMT, one trial reported data on AEs, and no AEs were observed)*	Not reported for SMT alone
Ruddock, J. K. et al. (2016)	9 RCTs (261 pts in total)	Pts with non-specific low back pain	SMT	Local discomfort, tiredness<br><br>No SAEs	"Only 3 trials reported on adverse events. Senna et al (2011)44 reported that the most common adverse events were local discomfort and tiredness, which had resolved within 24 hours. The other 2 articles just stated that none were reported.48,50 (…) Poor reporting of adverse events is a frequent criticism of complementary and alternative medicine research.61 Two previous reviews on complications of SM emphasize its safety62,63; however, serious adverse events have been reported.18 In this review, few studies reported on adverse events at all. In 1 study,48 the potential for adverse events to occur was higher because they used anesthetic to ensure adequate blinding of participants; this procedure may be considered an unnecessary risk. (…) There is some evidence that SM has specific treatment effects and is more effective at reducing nonspecific low back pain when compared with an effective sham intervention. However, given the small number	Not reported

Author (year) ref	Included studies on SMT (pts. in total receiving SMT)	Population receiving SMT	Interventions including SMT	AEs reported* associated with SMT)	Conclusion on AEs from SMT (quote)	Quality of the evidence for AEs (tool)
					of studies included in this analysis, we should be cautious of making strong inferences based on these results."	
Ruffini, N. et al. (2016)	1 RCT, 2 CSs, 2 CRs, 1 'mixed-methods design' (52 pts in total)	Women after menarche in all gynaecologic and obstetric conditions, including pregnancy, dysmenorrhea, labor, pelvic pain and menopause.	OMT (may include SMT)	None	"Considering the secondary outcome of the present systematic review, adverse events were not adequately described. Only three trials reported information on side effects, leading to insufficient data and therefore incomplete analysis. Certainly, more attention in planning, recording and analyzing adverse events is suggested for future trials. (…) In addition, the lack of data regarding potential harm does not mean that there is no risk of harm; nevertheless, even taking into account the limits mentioned above, none of the papers reported an increased risk of adverse events. (…) Although positive effects were found, the heterogeneity of study designs, the low number of studies and the high risk of bias of included trials prevented any indication on the effect of osteopathic care. Further investigation with more pragmatic methodology, better and detailed description of interventions and systematic reporting of adverse events are recommended in order to obtain solid and generalizable results."<br>*(Of the trials on HVLA, two trials reported data on AEs, and no AEs were observed)*	Not reported
Varatharajan, S. et al. (2016)	3 RCTs (129 pts in total)	Pts with headaches associated with neck pain (i.e., tension-type, cervicogenic, or whiplash-related headaches).	SMT	Headache<br><br>No SAEs	"Seven of the ten low risk of bias studies measured major adverse events and none were reported [15, 42, 46–48, 51] (Table 4). Five of the 10 RCTs reported minor adverse events that were mild to moderate and transient [15, 43, 46–48]. (…) The management of headaches associated with neck pain should include exercise. Patients who suffer from chronic tension-type headaches may also benefit from relaxation training with stress coping therapy or multimodal care. Patients with cervicogenic headache may also benefit from a course of manual therapy."<br>*(Of the trials on SMT, one trial reported data on AEs, and reported: "No serious adverse events. 6.7 % of headaches experienced during the intervention period were reported as 'provoked by treatment' by the subjects")*	Not reported
Wearing, J. et al. (2016)	1 CR, 1 CS, 2 pilot RCTs, 1 preliminary RCT, 1 pre-post study (55 pts in total)	Pts with chronic obstructive pulmonary disease	SMT (including OMT)	Muscle soreness<br><br>No SAEs	"Three studies21,22,25 reported on adverse events following SMT, describing a small number of minor adverse events and no moderate or severe adverse events. Minor adverse events are described as muscle soreness up to 24 hours after treatment that resolves with no further intervention. (…)In conclusion, this appears to be the first systematic review to investigate the evidence for administering SMT in conjunction with other modalities, such as exercise, on people with COPD. The exclusion of such combinations may explain the disparity in findings between this review and the review by Heneghan et al., who found no evidence to support or refute the use of MT in the management of COPD. The importance of increasing exercise capacity even by indirect methods such as increasing thoracic mobility should not be underestimated because exercise capacity is a predictor of mortality in COPD. As PR does not improve lung function, the current findings may have wider implications if repeated in a larger cohort. The results of this systematic review support the recommendation for further research in the field, in particular a larger RCT	Not reported

Author (year) ref	Included studies on SMT (pts. in total receiving SMT)	Population receiving SMT	Interventions including SMT	AEs reported* associated with SMT)	Conclusion on AEs from SMT (quote)	Quality of the evidence for AEs (tool)
					designed to investigate the effect of combining SMT with exercise."	
Wong, J. J. et al. (2016)	7 RCTs (369 pts in total)	Pts with whiplash-associated disorders or neck pain and associated disorders	SMT	Soreness, minor increase in neck pain or fatigue, increased neck pain and headache<br><br>No SAEs	"4. The risk of serious adverse events associated with manipulation was extremely low. (…) Sixteen of the 22 studies with a low risk of bias addressed the occurrence of adverse events [34,36–38,40,41,43–47,49,50,54,73,74]. Most adverse events were mild to moderate and transient (Tables 6 and 7). No serious neurovascular adverse events were reported. Most studies had a rate of minor adverse events ranging from 0% to about 30% [40,42–44,46–54,56]. One study [49] reported mild and transient adverse events in 98.9% of patients who received high dose strengthening exercise therapy and spinal manipulation, and 96.6% who received the same exercise therapy alone. Two serious adverse events in patients allocated to cervical mobilization were reported in one study, but were reported as unrelated to treatment by the attending medical specialists (one participant had a cardiac event, and one participant developed severe arm pain and weakness 3 days after the mobilization session) [50]. (…) Results that are consistent with findings of the Neck Pain Task Force Evaluation studies: We found that cervical manipulation and cervical mobilization lead to similar outcomes in individuals with recent NAD grades I–II. We also found that there were no serious adverse events reported in randomized clinical trials on manipulation. We did not find any studies that compared different techniques of cervical manipulation; therefore, it is unclear if specific cervical manipulation techniques are more effective than others."<br>*(Of the RCT referred to, [43], [44], [49], [50], [56], and [59] includes a group with SMT)*	
Yao, M. et al. (2016)	19 RCTs (1135 pts in total)	Pts with neck pain	SMT	Increased neck or headache pain, fatigue, nausea, dizziness<br><br>**SAEs:** Not specified	"Adverse events (AEs) as a measure of outcomes were reported in 10 studies,(40-45,50,51,53,54) out of 19 trials, and 4 trials(42,43,51,53) reported no serious AEs during treatment in any group. There were 6 trials(40,41,44,45,50,54) reported AEs. Only 1 patient of manipulative therapy group in 1 trial(54) was withdrawn due to serious AE, while in 5 trials,(40,41,44,45,50) there were AEs related to increased neck or headache pain, fatigue, nausea, and dizziness in the control group only or both the control group and manipulative therapy group as well. (…)This review does not suggest that the existing evidence supports the value of Eastern and Western manipulative therapy for neck pain of short and intermediate-duration. However, the limitations of the trials do not allow us to make a definitive conclusion."	Not reported
Cicchintti L. et. al (2015)	1 CC, 6 RCTs, 1 observational study, 1 laboratory study, 1 cross-over pilot study (NA)	Pts 'with medical conditions classified also as CID' (CID: chronic inflammatory disease)	OMT (may include SMT)	Musculoskeletal soreness or pain, elevated blood pressure in the morning, mild heart palpitations, sore back, feeling mildly light headed<br><br>No SAEs	"No major side effects were reported by those receiving OMT. (...) the OMT appears to be a safe approach (...) Only seven studies reported data on side effects. In five studies none of the participants showed side effects after osteopathic treatments. In the study conducted by Noll et al., 14 subjects over 25 reported mild side effects characterized by musculoskeletal soreness or pain. Post-hoc calculation of RR showed a reduction of side effects in the minimal touch control group compared to all other groups (data not showed). A further study conducted by Noll et al reported two patients with symptoms of muscle soreness after the OMT	Not reported

Author (year) [ref]	Included studies on SMT (pts. in total receiving SMT)	Population receiving SMT	Interventions including SMT	AEs reported* associated with SMT)	Conclusion on AEs from SMT (quote)	Quality of the evidence for AEs (tool)
					session, while in the sham group the side effects were recorded in four subjects who reported "elevated blood pressure in the morning", "mild heart palpitations", "a little muscle soreness" and "back was a little sore". Again, post-hoc RR computations demonstrated no significant reduction of side effects in the study group compared to controls (data not showed)."	
Franke, H. et al. (2015)	3 RCTs (67 pts in total)	Pts with chronic nonspecific neck pain	OMT (may include SMT)	Tiredness, short-term aggravation of symptoms in other 'familiar' regions<br><br>No SAEs	"Based on the 3 included studies, the review suggested clinically relevant effects of OMT for reducing pain in patients with chronic nonspecific neck pain. Given the small sample sizes, different comparison groups, and lack of long-term measurements in the few available studies, larger, high-quality randomized controlled trials with robust comparison groups are recommended. (…) Only 1 of the 3 studies reported on adverse events. Schwerla et al. stated that no serious adverse events were reported during the treatment period although transient minor events, such as tiredness on the day of treatment and short-term aggravation of symptoms in other 'familiar' regions, were noted. The other 2 studies did not report on adverse events. In personal communications, the authors of these studies indicated that no adverse events occurred."	NA (GRADE)
Gross A. J. et. al (2015)	39 RCTs (NA)	Pts with cervical pain, cervicogenic headache or cervical disorders with radicular findings	Manipulation (may be entirely SMT)	Increased neck pain, soreness, headache, stiffness, dizziness, nausea, paraesthesia, upper limb pain, fatigue, mid-lower back pain and "unpleasant change in spinal posture"<br><br>No SAEs	"Among participants receiving manipulation, 22% (105/469 participants) experienced adverse events. All adverse events reported for manipulation or mobilisation were benign and transient side effects (…)"	Not reported
Liddle S. D. & Pennick V. (2015)	5 RCTs (289 pts in total)	Pregnant women with risk for or suffering from low-back pain, pelvic pain or both	SMT, manual therapy provided by a chiropractic specialist, osteopathic treatments, OMT (all treatments include or may include SMT)	NA<br><br>No SAEs	"When reported, adverse effects were minor and transient." *(Of the five trials on SMT, two did not report on AEs, two trials reported no AEs, and for one trial it was unclear if AEs was reported or not)*	Not reported
Posadzki, P. et al. (2015)	5 SRs (NA pts in total)	Pts (adults, adolescents or children) with migraine	SMT (including a few SRs including other types of manual therapy as well)	Not specified/may be none<br><br>**SAEs:** Not specified/may be none	"In multiple SRs, positive conclusions were reached for AT (n=9), BFB (n=2), P. hybridus (n=1), T. parthenium L. (n=2) and SMT (n=2) (Table 6). Negative conclusions were reported for homeopathy (n=1) and SMT (n=1). Equivocal conclusions were reached for AT (n=3), homeopathy (n=2), P. hybridus (n=2), T. parthenium L. (n=4) and SMT (n=2). For other types of CAM, positive conclusions were drawn for AA, chiropractic, HBOT, manual therapies and relaxation (Table 7). (…) Twenty-two (66.7%) SRs mentioned adverse effects (AEs) and 11 did not. Sixteen (48.4%) SRs disclosed the authors' conflicts of interest, whereas 17 did not. Nineteen (57.5%) SRs reported the source of funding and 14 did not. (…) A large number of SRs of CAM	Not reported

Author (year) [ref]	Included studies on SMT (pts. in total receiving SMT)	Population receiving SMT	Interventions including SMT	AEs reported* associated with SMT)	Conclusion on AEs from SMT (quote)	Quality of the evidence for AEs (tool)
					for the prevention and/or treatment of migraine exist. The evidence for the effectiveness of CAM in preventing or treating migraines is, in the majority of SRs, positive. Policy makers and clinicians should acknowledge the existence of several caveats, however. For instance, only for acupuncture and biofeedback are the conclusions unanimously positive, whereas for the remainder of CAM modalities, the evidence is still conflicting."<br>*(All of the SRs on SMT mentioned AEs)*	
Puentedura E. J. & O'Grady W. H. (2015)	7 CRs<br>(10 pts in total)	Pts in studies reporting AEs following thoracic SMT	Thoracic SMT	Progressive weakness/paraesthesia in the lower extremities, thoracic pain, nausea, shortness of breath/dyspnoea at rest, neck stiffness, photophobia, severe headache<br>(*most common*)<br><br>**SAEs**: Injury (mechanical or vascular) to the spinal cord, pneumothorax, hematothorax, cerebrospinal fluid leak secondary to dural sleeve injury<br>(*most common*)	"This review showed that serious AEs do occur in the thoracic spine. The most commonly reported AE involved trauma to the spinal cord, followed by pneumothorax. This suggests that excessive peak forces may have been applied to thoracic spine, and it should serve as a cautionary note for clinicians to work on their TJM skills to decrease these peak forces."	Not reported
Southerst D. et. al (2015)	2 RCTs<br>(98 pts in total)	Pts with thoracic spine pain or acute chest pain	Cervical or thoracic SMT	Local tenderness, headache, fatigue<br><br>No SAEs	"One study reported on adverse events. Stochkendahl et al reported no serious adverse events in the multimodal care group [including SMT]. However, 75% of participants in this group reported transient and benign adverse events such as local tenderness, headache, and fatigue."	Not reported
Yuan Q.-L. et. al (2015)	3 RCTs<br>(208 pts in total)	Pts with cervical pain	Chinese manipulation	None	"Two studies mentioned adverse events; none were observed in either study."	Not reported
Zhu L. et. al (2015)	3 RCTs<br>(NA)	Pts with degenerative cervical radiculopathy	Cervical SMT	None	"The safety of cervical manipulation cannot be taken as an exact conclusion so far. (...) Only one trial reported the adverse events and none were observed in the trial with a small sample size. The other two trials did not mention whether adverse events have occurred in the intervention or control group."	Not reported
Bryans R. et. al (2014)	12 RCTs<br>(513 pts in total)	Pts with cervical pain	Manipulation or thoracic manipulation	"Minor events"<br><br>No SAEs	"There were no serious adverse events reported in any of the citations used in developing these treatment recommendations. A summary of the adverse event reporting from the literature summary is shown in Table 7. Of the 43 studies included in this summary, 14 made no mention of adverse events. Of the remaining 33, all studies reported either none or only minor adverse events from a total of 1682 study participants and several treatment sessions (on average) per participant."<br>*(The tables shows, that 8 trials reported no AEs, 3 trials did not record AEs, and 1 trial reported minor events)*	Not reported

Author (year) [ref]	Included studies on SMT (pts. in total receiving SMT)	Population receiving SMT	Interventions including SMT	AEs reported* associated with SMT)	Conclusion on AEs from SMT (quote)	Quality of the evidence for AEs (tool)
Clar C. et. al (2014)	96 RCTs, 72 SRs, 10 non-randomized primary studies (NA)	Pts 'with musculoskeletal and non-musculoskeletal conditions'	Interventions including 'an element of manipulation or mobilisation'	Worsening symptoms, increased pain, soreness, headache, dizziness, tiredness, nausea, vomiting (*very sparse information with respect to AEs*)<br><br>**SAEs**: Cerebrovascular events, disc herniation, vertebral artery dissection, cauda equine syndrome, stroke, dislocation, fracture, transient ischemic attack	"Poorly and scarcely reported harms data limited our ability to make meaningful comparisons of rates of adverse events between the treatments (...) Seven systematic reviews and seven primary studies were identified specifically concerning adverse events of manual therapy. Mild-to-moderate adverse events of transient nature (...) were relatively frequent. For example, evidence from high, medium, and low quality systematic reviews specifically focusing on adverse events suggested that approximately half of the individuals receiving manual therapy experienced mild-to-moderate adverse event which had resolved within 24–74 hours. In agreement with the UK evidence report, evidence indicated that serious (or major) adverse events after manual therapy were very rare (...). Evidence on safety of manual therapies in children or pediatric populations was scarce; the findings from two low quality cohort studies and one survey were consistent with those for adults that transient mild to moderate intensity adverse events in manual treatment were common compared to more serious or major adverse events which were very rare."	Not reported
Close C. et. al (2014)	1 RCT, 1 feasibility RCT (NA)	Pregnant women with low-back pain or pelvic pain	SMT or OMT	Soreness *(very sparse information with respect to AEs)*<br><br>No SAEs	"In this review, adverse effects reported were minor, which would imply that participants that dropped out were unhappy with the treatment they were receiving, or they may have perceived no improvements. However, possible under-reporting of adverse effects cannot be ruled out, considering the few studies that reported adverse effects and the limited detail provided. (...) There was also limited information on adverse effects. This is a cause for concern as the under-reporting of adverse effects could make treatments appear safer than they actually are, as well as possibly breaching publication ethics."	Not reported
Franke H. et. al (2014)	15 RCTs (779 pts in total)	Pts with non-specific low-back pain	OMT (may include SMT)	Stiffness and tiredness *(very sparse information with respect to AEs)*<br><br>No SAEs	"Of the 15 included studies, only 4 studies reported on adverse events. Two studies reported minor adverse events such as stiffness and tiredness. In the 2013 study, Licciardone et al. reported that 6% of patients had adverse events, but none of the serious events appeared to be related to the treatment intervention, and there were no significant differences between the treatment groups in the frequency of adverse events or serious adverse events. In a personal communication, the authors of another study reported that no adverse events occurred."	Not reported
Kizhakkeveettil A. et. al (2014)	12 RCTs (1799 pts in total)	Pts with low-back pain	SMT	Not specified/may be none<br><br>**SAEs**: Not specified/may be none	None<br>*(Unclear descriptions. No AEs were reported in 2 RCTs, AEs may have been reported in 4 RCTs and AEs were not reported in 6 RCTs)*	Not reported
Page M. J. et. al (2014)	1 RCT *(9 other trials included manual therapy, but were not further specified and were not included)* (4 pts in total)	Pts with adhesive capsulitis (frozen shoulder)	SMT	None	"Only seven trials measured adverse events, with three reporting marginal differences between groups, and four reporting no adverse events in any group [including one trial on SMT]."	Not reported (*The SMT study was not included in the quality of evidence assessment of AEs (GRADE)*)

Author (year) ref	Included studies on SMT (pts. in total receiving SMT)	Population receiving SMT	Interventions including SMT	AEs reported* associated with SMT)	Conclusion on AEs from SMT (quote)	Quality of the evidence for AEs (tool)
Sutton D. et. al (2014)	8 RCTs<br>(813 pts in total)	Pts diagnosed with whiplash-associated disorders or cervical pain and associated disorders	Multimodal care (SMT mixed with other interventions)	Headache, increased neck pain, tingling in upper extremities, dizziness, odd arm sensation but had normal neurologic examination, muscle soreness, tiredness, increased pain after first and second appointments (*the interventions ware very mixed, and all of these AEs may not be related to SMT*)<br><br>No SAEs	"Nine admissible RCTs reported adverse events [7 of these included SMT]. No RCT reported serious adverse events. Most adverse events were minor (e.g., headache, increased neck pain, tingling in upper extremities, dizziness). The proportion of adverse events in participants enrolled in a multimodal program of care ranged from 3% after one multimodal osteopathic treatment [in a study including SMT] to 63% after a multimodal program of care [in a study not including SMT]."	Not reported
Todd A. J. et. al (2014)	4 CRs, 1 CS, 8 surveys, 6 RCTs, 5 SRs, 5 narrative reviews, 2 discussion papers<br>(>34605 pts in total)	Infants or children in studies reporting AEs following chiropractic or other manual treatments	Chiropractic and other manual treatments (including SMT in the majority of the studies)	Soreness, stiffness, headache, severe headache, crying, sleeplessness, mid-back soreness and increased irritability, stiff neck, moderate to severe bradycardia, apnea of short duration, worsening symptoms, behavior problems/irritability, pain/soreness, headache, dizziness/flu-like symptoms/treatment, reaction/tiredness, vomiting, left facial weakness, diplopia, ataxia, leg fractures, hematothorax<br><br>**SAEs**: Loss of consciousness, anterior dislocation of atlas and fracture of odontoid axis at C2, dislocation of atlas, death, subarachnoidal hemorrhage and death	"High-velocity, low-amplitude thrust (HVLA) spinal manipulative therapy (SMT) was applied in 10 of the 15 cases of serious adverse events. In addition, in 8 of the 15 cases of a serious adverse event, it was revealed that before the application of chiropractic or manual therapy, there was present a preexisting but undetected underlying pathology or existing neurologic symptoms. Three deaths were recorded, and 2 of these were in infants under 3 months of age who had previously been healthy."	Not reported
Tuchin P. (2014)	9 CRs<br>(9 pts in total)	Pts experiencing intracranial hypotension following SMT	SMT	Intracranial hypotension<br><br>No SAEs	"To date, the evidence that CSMT [chiropractic SMT] is a cause of IH [intracranial hypotension] is inconclusive."	Not reported
Yin P. et. al (2014)	34 CRs, 4 CSs<br>(94 pts in total)	Pts experiencing AEs following pain-related massage (including SMT)	Different types of manipulations (SMT or not further specified)	Disc herniation, soft tissue trauma, neurologic compromise, bone fracture, hematoma or hemorrhagic cyst, syncope, pain, dislocation (*most common*)<br><br>**SAEs**: Spinal cord injury,	"The symptoms are frequently life-threatening, though in most cases the patient made a full recovery. In the majority of cases, the problems were related to spinal manipulations, including rotational movements, which seem to be the probable cause of the AEs. (...) Spinal manipulation in massage has repeatedly been associated with serious AEs especially. But the incidence of such events is probably low."	Not reported

Author (year) ref	Included studies on SMT (pts. in total receiving SMT)	Population receiving SMT	Interventions including SMT	AEs reported* associated with SMT)	Conclusion on AEs from SMT (quote)	Quality of the evidence for AEs (tool)
				dissection of the vertebral arteries, cauda equina syndrome (*most common*)		
Young J. L. et. al (2014)	1 CS, 1 pCohort, 10 RCTs, 1 'quasi-experimental study lacking randomization', 1 'secondary analysis of a RCT' (539 pts in total)	Pts with mechanical neck pain	Thoracic SMT	Aggravation of symptoms, muscle spasm, neck stiffness, headache, and radiating symptoms<br><br>No SAEs	"In addition, no significant differences were observed in the number of side effects experienced by the manipulation or mobilization groups." *(No descriptions linking the AEs to the individual trials)*	Not reported
Brantingham J. W. et. al (2013)	5 CRs, 2 CSs, 1 RCT (109 pts in total)	Pts with upper extremity problems (including carpal tunnel syndrome, shoulder impingement syndrome, soft tissue disorder or associated myofascial pain and dysfunction syndrome, frozen shoulder adhesive capsulitis, Parsonage-Turner syndrome, temporomandibular joint dysfunction and disorder, lateral epicondylitis, epicondylosis, epicondylalgia, tennis elbow, etc.)	SMT	Not specified<br><br>**SAEs**: Not specified/may be none	"Yet with local MMT [Manual or Manipulative Therapy] management of CTS [carpal tunnel syndrome] there are no reported serious adverse reactions beyond occasional minimal and temporary soreness, stiffness and/or temporary aggravation; bruising and/or soreness from soft tissue MMT [not SMT]. Such bruising and soreness caused only one subject to leave treatment in the Burke et al study and none in the vigorous ST MMT (trigger point therapy) CTS study of Hains and Hains [not SMT]. There are then almost no reports of serious adverse reactions (permanent disability or death) and minimal to nearly no side-effects reported for ultrasound, splinting, mobilization of the carpal bones, and/or upper extremity FKC MMT [Full Kinetic Chain Treatment Manual or Manipulative Therapy] in treatment of CTS [including 2 studies on SMT]."	Not reported
Hebert J. J. et. al (2013)	41 CRs (77 pts in total)	Pts experiencing SAEs following SMT of the lumbar spine or pelvis	SMT of the lumbar spine or pelvis (including a few studies on spinal mobilization)	Lumbar disk herniation, fracture, hematoma or hemorrhagic cyst, soft tissue trauma, muscle abscess formation, disrupted fracture healing<br><br>**SAEs**: Signs and symptoms consistent with cauda equina syndrome, neurologic or vascular compromise, esophageal rupture	"Additional high-quality research is needed to better estimate the incidence of adverse events associated with lumbopelvic SMT and to elucidate the relationship between this therapy and the types of adverse events reported in this systematic review. (…) The most commonly reported adverse events were signs and symptoms consistent with cauda equina syndrome (29 cases, 38% of total) and lumbar disk herniation (23 cases, 30% of total)."	Not reported
Huisman P. A. et. al	10 RCTs	Pts with non-specific cervical	Thoracic SMT	"Benign transient side effects"	"Five studies provided information regarding adverse events, which if occurred, were benign transient side effects. In future	Not reported

Author (year) [ref]	Included studies on SMT (pts. in total receiving SMT)	Population receiving SMT	Interventions including SMT	AEs reported* associated with SMT)	Conclusion on AEs from SMT (quote)	Quality of the evidence for AEs (tool)
(2013)	(350 pts in total)	pain		No SAEs	studies, better reporting of adverse events is needed."	
Parkinson L. et. al (2013)	1 pCohort, 4 RCTs, 1 observational study (>520 pts in total)	Pts with lower back pain	SMT	None	"(...) and two considered adverse events. (…) Giles et al. found that patients were highly satisfied with chiropractic treatment, and that no adverse events related to chiropractic occurred in a hospital setting. (…) Both studies which considered this reported no adverse events associated with chiropractic, but this was not significantly different from other treatments in the UCLA low back pain study." *(AEs reported by two studies, both reported no AEs)*	Not reported
Posadzki P. et. al (2013)	17 RCTs (>448 pts in total)	Children and adolescents with 'pediatric conditions'	OMT (may include SMT)	"Aggravation of vegetative symptoms"<br><br>No SAEs	"Eleven (64%) of the included RCTs failed to report the incidence rates of AEs. This may amount to a serious breach of publication ethics. (…) Four RCTs mentioned that no AEs had occurred. Philippi et al reported that 4 patients had had aggravation of vegetative symptoms after OMT. Two AEs reported in the study by Wahl et al were related to Echinacea and placebo and not to OMT."	Not reported
Scholten-Peeters G. G. M. et. al (2013)	19 RCTs (626 pts in total)	Pts 'with a diversity of complaints'	Manipulative therapy (mostly SMT)	Minor aggravation of neck pain or headache, muscle soreness, stiffness, tiredness, and local discomfort<br><br>No SAEs	"Only a few minor adverse events were reported in the included studies. There were no serious complications such as stroke." *(AEs reported in 4 RCTs, no AEs reported in 4 RCTs, AEs not reported in 11 RCTs)*	Not reported
Schroeder J. et. al (2013)	3 RCTs (195 pts in total)	Pts with cervical pain	Cervical SMT	Not specified<br><br>**SAEs**: Not specified/may be none	"[Acute Neck Pain:] Reported complications were minor and were similar between manipulation therapy compared with home exercise and mobilization therapy compared with physical therapy treatment groups. [Chronic Neck Pain:] There were no significant differences in treatment complications reported when comparing subjects who underwent spine manipulation therapy to those who received exercise." *(From tables: 'Minor complications of treatments' for acute neck pain: SMT, 40% (37/91), 'Home exercise', 46% (42/91), Effect size (95% CI), 0.86 (0.61-1.20); 'Complications/side effects' for chronic neck pain: SMT, 9.4% (6/64), 'Exercise', 14.3% (9/63), Effect size (95% CI), 0.66 (0.25-1.74))*	Not reported
Wynd S. et. al (2013)	24 CRs, 14 CSs, 2 surveys, 2 cohort studies, 1 commentary (901 pts in total)	Pts experiencing cervical artery dissection or stroke following cervical SMT	Cervical SMT	NA<br><br>**SAEs**: Cervical artery dissection (901 cases), stroke (707 cases)	"This study has demonstrated that the literature infrequently reports useful data toward understanding the association between cSMT [cervical spinal manipulation therapy], CADs [cervical artery dissection] and stroke. Improving the quality, completeness, and consistency of reporting adverse events may improve our understanding of this important relation."	Not reported
Yang M. et. al (2013)	2 RCTs (39 pts in total)	Pts with any type of pneumonia	OMT (may include SMT)	Muscle tenderness<br><br>No SAEs	"Only one trial reported adverse effects, as transient muscle tenderness emerged after treatment in two individuals during the period of study."	Not reported
Brantingham J. W. et. al (2012)	2 CRs, 2 CSs, 7 RCTs, 2 controlled or clinical trials, 2 single-group pretest-posttest designs	Pts with upper extremity conditions (including hip osteoarthritis, hip strain, patellofemoral	SMT (may include some mobilization)	'Minor side effects', "mild posttreatment soreness after the first 1-2 treatments, which resolved in all patients."	"Nevertheless, overall, when appraising the increasing quantity and quality of included trials, MT [manipulative therapy] for lower extremity disorders appears to be of value and, like spinal MT, fundamentally safe." *(3 studies reported 'side effects' (including one study reporting no AEs), 1 study reported no 'side effects', and AEs or 'side*	Not reported

Author (year) [ref]	Included studies on SMT (pts. in total receiving SMT)	Population receiving SMT	Interventions including SMT	AEs reported* associated with SMT)	Conclusion on AEs from SMT (quote)	Quality of the evidence for AEs (tool)
	(SGPPDs) (>109 pts in total)	pain syndrome, acetabular anterosuperior labral tear, plantar fasciitis		No SAEs	*effects' were not mentioned for the remaining studies)*	
Dobson D. et. al (2012)	4 RCTs (116 pts in total)	Infants suffering from colic)	SMT (including one study that did not specify the chiropractic treatment)	None	"One of the studies recorded adverse events and none were encountered. However, with only a sample of 325 infants, we have too few data to reach any definitive conclusions about safety. (…) No adverse effects were found, but they were only evaluated in one of the six studies. (...) we cannot quantify any risk of adverse effects when using manipulative therapies for the treatment of infantile colic. (...) Only one study (Miller 2010; N = 102) reported findings for adverse outcomes. None were recorded. A case report was incidentally drawn to our attention during the review process. This report outlines the case history of an individual infant who died following treatment for infantile colic by a "so called CranioSacral Therapist" (Holla 2009) who appears to have used an unrecognised technique. We have not undertaken a systematic search for safety studies, although we have introduced the debate in the background section. We may consider a comprehensive search specifically for adverse effects in the update of this review."	"Not estimable" (GRADE)
Furlan A. D. et. al (2012)	2 CCs, 2 pCohorts, 22 RCTs (NA)	Pts in studies receiving SMT	SMT	Transient increased pain (*very sparse information with respect to AEs*)<br><br>**SAEs**: Vertebro-basilar artery (VBA) stroke, cervical artery dissection *(very sparse information with respect to SAEs)*	"Poorly and scarcely reported harms data limited our ability to meaningfully compare rates of adverse events between the treatments. (...) RCTs: The reported events in RCTs were mostly moderate in severity and of transient nature (e.g., increased pain). In one RCT, after 2 weeks of treatment, patients with neck pain receiving manipulation were not at significantly increased risk for having an adverse event compared to patients receiving mobilization (OR = 1.44, 95% CI: 0.83, 2.49). In another RCT, the proportion of patients with neck pain having adverse events was similar in manipulation versus Diazepam groups (9.5% versus 11.1%). Nonrandomized Studies: In two case control studies, subjects younger than 45 years of age with vertebro-basilar artery (VBA) stroke were more likely to visit a chiropractic or primary care physician than subjects without VBA stroke. This association was not observed in older subject visiting the chiropractic clinic. In the first case-control study, the excess risk of vascular accident was observed for both, subjects undergoing chiropractic care and subjects undergoing primary care treatments. In the second case-control study, subjects with cervical artery dissection were more likely to have had spinal manipulation within 30 days (OR = 6.62, 95% CI: 1.4, 30.0). In one cohort study, rate of complications did not differ between subjects with low-back pain receiving manipulation plus mobilization versus no treatment. In another prospective cohort study of 68 subjects with chronic LBP, treatment with medication-assisted manipulation or spinal manipulation alone for at least 4 weeks did not lead to any complications requiring institutional review board notification."	Not reported
Gleberzon B. J. et. al	1 CS, 1 pCS, 6 RCTs, 2 pilot RCTs, 1	Pts below 18 years	SMT	Not specified	"SMT was safely used, with only two transient, self-limiting adverse reactions reported. [from the feasibility pilot study] (…) No adverse effects were reported in any of the clinical	Not reported

Author (year) ref	Included studies on SMT (pts. in total receiving SMT)	Population receiving SMT	Interventions including SMT	AEs reported* associated with SMT)	Conclusion on AEs from SMT (quote)	Quality of the evidence for AEs (tool)
(2012)	randomized feasibility study, 1 controlled clinical trial, 2 prospective study, 1 retrospective evaluation, 1 retrospective study (NA)			**SAEs**: May be none	trials reviewed."	
Haynes M. J. et. al (2012)	5 CCs (NA)	Pts in studies reporting craniocervical artery dissection associated with cervical SMT	Cervical SMT	NA<br><br>**SAEs**: Cranio-cervical artery dissection, vertebrobasilar occlusive stroke, vertebral artery dissection	"All of the extracted studies yielded inconclusive evidence regarding a strong association or no association between cSMT [cervical spinal manipulative therapy] with CAD [carotid artery dissection] related stroke."	Not reported
Kuczynski J. J. et. al (2012)	6 RCTs (268 pts in total)	Pts with low-back pain	SMT	Aggravation of symptoms and stiffness (*most common*)<br><br>No SAEs	"Physical therapy spinal manipulation appears to be a safe intervention that improves clinical outcomes for a variety of patients with LBP. (...) Only one study reported the presence of adverse effects. Cleland et al found that 25 percent of patients within the study reported these side effects. Nine patients in each spinal manipulation group reported side effects, whereas 10 patients in the nonthrust manipulation (comparative) group reported such effects. Although no serious complications were reported, the most common side effects included aggravation of symptoms and stiffness. All adverse effects were reported to be resolved within 48 hours of onset."	Not reported
Lin J. H. et. al (2012)	4 RCTs (283 pts in total)	Pts with mechanical neck pain (cervical spondylotic radiculopathy or cervical spondylosis)	Chinese manipulation	None	"No adverse events were reported in the four studies. (…) Only one study mentioned adverse events and none was observed in that study. The other studies did not report whether adverse events had been measured in the trials. (...) The adverse event rate of Chinese manipulation in treating neck pain was not clear."	Not reported
Posadzki P. & Ernst E. (2012)	5 RCTs (NA)	Pts with tension-type headache	SMT	Neck stiffness, minor aggravations of neck pain or headaches<br><br>No SAEs	"Three studies reported adverse effects (AEs) and two RCTs failed to provide that information. Several hundred severe complications after upper spinal manipulations have been reported. The estimates as to the incidence of these complications vary hugely. Not reporting AEs is unhelpful and distorts the overall picture about AEs after SM. It also is also not in line with generally accepted research ethics."	Not reported
Puentedura E. J. et. al (2012)	93 CRs (134 pts in total)	Pts experiencing AEs following cervical SMT	Cervical SMT	Disc herniation, weakness, paresthesias, and increased pain<br><br>**SAEs**: Arterial dissection, cerebrovascular accident, vertebral dislocation or fracture, death	"This review showed that, if all contraindications and red flags were ruled out, there was potential for a clinician to prevent 44.8% of AEs associated with CSM [cervical spine manipulation]. Additionally, 10.4% of the events were unpreventable, suggesting some inherent risks associated with CSM even after a thorough exam and proper clinical reasoning. (...) Four of the [seven] deaths were determined to be preventable, one unpreventable and two unknown. (...) Arterial dissection was the most common AE reported, being present in 37.3% of the cases (n=550). Other common AEs	Not reported

Author (year) ref	Included studies on SMT (pts. in total receiving SMT)	Population receiving SMT	Interventions including SMT	AEs reported* associated with SMT)	Conclusion on AEs from SMT (quote)	Quality of the evidence for AEs (tool)
					included disc herniation (18.7%, n=525), CVA [cerebrovascular accident] (13.4%, n=518), and vertebral dislocation or fracture (6.7%, n=59)."	
Rubinstein S. M. et. al (2012)	20 RCTs (1195 pts in total)	Pts with acute low-back pain	SMT	Aggravation of symptoms, stiffness (*most common*)<br><br>No SAEs	"Importantly, there was no evidence of serious adverse events demonstrated in any of the trials, although all RCTs were too small to give any reliable and precise estimate of these types of events (...) Six studies, with a total of 1195 participants, reported on adverse events. One study reported four serious adverse events, occurring equally in both the experimental and control groups; however, "neither of the events appeared to be related to the allocated treatment strategies" (Juni 2009). In another study 25% of the participants reported at least one side effect of treatment; however, there were no differences between the groups and all symptoms resolved within 48 hours of onset (Cleland 2009)."	"Not estimable" relative effect for SAEs and no grading given (GRADE)
Stuber K. A. et. al (2012)	4 CRs, 1 pCohort, 2 SRs (NA)	Females who are pregnant or postpartum (period from giving birth to six weeks after) and experiencing AEs following SMT	SMT	Vertigo, paresthesias<br><br>**SAEs**: Right cerebral infarct, occlusion of the left vertebral artery and thrombus in basilar artery, pathological type II odontoid fracture with ventral displacement producing spinal cord compression and paravertebral hematoma, epidural hematoma	"There are only a handful of reported cases of adverse events following spinal manipulation during pregnancy and the postpartum period in the literature with the severity ranging from mild increases in pain that resolved quickly to significant life-threatening injuries. While improved reporting of such events is required in the future, it may be that such injuries are relatively rare. Clearly future research into efficacy of this treatment for these populations and the rates of occurrence of adverse events is necessary to determine whether or not this is true."	"lower levels of evidence" (the hierarchy of evidence)
Brantingham J. et. al (2011)	2 CRs, 1 CS, 5 RCTs, 1 controlled trial (CT), 1 single-arm trial, 1 investigational study (>266 pts in total)	Pts with shoulder pain and disorders (including shoulder complaints, dysfunction, disorders, and/or pain, shoulder impingement syndrome, rotator cuff injuries, disease or disorders, acromioclavicular injury, osteoarthritis, frozen shoulder, neurogenic shoulder pain, glenoid hypoplasia)	SMT	None	None<br>*(4 studies reported no AEs, 2 studies did not report AEs, and AEs was not mentioned for the 5 remaining studies)*	Not reported
Cross K. et. al (2011)	6 RCTs (187 pts in total)	Pts with mechanical neck pain	Thoracic SMT	Aggravation of symptoms, muscle spasm, headache. (a duration of no greater than 24 hours)	"Only 2 of the included studies presented complications or adverse events as a result of the interventions. Cleland et al reported no significant differences in the number of side effects experienced by individuals in the thrust manipulation versus nonthrust group. (...) In a later study by Cleland et al, no adverse events in either group throughout the trial were	Not reported

Author (year) [ref]	Included studies on SMT (pts. in total receiving SMT)	Population receiving SMT	Interventions including SMT	AEs reported* associated with SMT)	Conclusion on AEs from SMT (quote)	Quality of the evidence for AEs (tool)
				No SAEs	reported."	
Huang T. et. al (2011)	2 RCTs (131 pts in total)	Children with nocturnal enuresis	SMT (chiropractic adjustments of the spine)	Headache, stiff neck, acute pain in lumbar spine<br><br>No SAEs	"Adverse effects identified in eight of the 24 RCTs were generally mild and self-limiting. However, the adverse effects could not be attributed to the trial treatments with certainty. Furthermore, the majority of the trials (15) failed to mention whether or not there were adverse effects. (...) However, judged on the evidence available adverse effects of these therapies seemed to be generally mild."<br>*(This conclusion applies to all 24 included RCTs - no conclusion was available for only the two RCTs on SMT. For two RCTs on SMT, only one RCTs reported AEs, the other did not mention AEs)*	Not reported
Lystad R. P. et. al (2011)	6 pCohorts, 3 RCTs (NA)	Pts with cervicogenic dizziness	SMT	"Minor adverse reactions"<br><br>No SAEs	"Only three studies commented on adverse reactions. Two RCTs [did not include SMT] reported no adverse reactions, and one prospective cohort study [included SMT] found minor adverse reactions associated with the interventions in eight of nineteen participants."	Not reported
Posadzki P. & Ernst E. (2011)	9 RCTs (NA)	Pts with cervicogenic headache	SMT	Hot skin, dizziness, headache, "minimal benign reactions lasting less than 24 hours"<br><br>No SAEs	"The majority of RCTs failed to provide details of adverse effects. (...) Four of the 9 RCTs reported adverse effects (AEs). Five RCTs failed to provide that information. The non-reporting of AEs is in violation of all guidelines of reporting clinical trials and, arguably, of medical ethics. It is also worth noting that several hundred severe complications after upper spinal manipulations have been reported (e.g., Ernst and Terrett). A particular concern relates to vascular accidents caused by arterial dissection after upper spinal manipulation. The estimates as to the incidence of these complications vary hugely. Underreporting of AEs in RCTs is likely to generate a false impression about the safety of SM."	Not reported
Posadzki P. & Ernst E. (2011)	3 RCTs (NA)	Pts with migraine headache	SMT	Neck pain and soreness. *(very sparse information with respect to AEs)*<br><br>No SAEs	"Two studies (out of three) reported adverse effects; and one RCT failed to provide such information. In the study by Parker et al. the likelihood of adverse effects was statistically significant in the SMT group (p<0.005)."	Not reported
Posadzki P. & Ernst E. (2011)	6 SRs (NA)	Pts suffering from headaches	SMT (may include some mobilization and other manual therapies)	Not specified/may be none<br><br>**SAEs**: Not specified/may be none	"Several of the included SRs fail to mention the important issue of adverse effects after SM."<br>*(Incomplete descriptions, 3 trials mention AEs, 3 trials do not mention AEs)*	Not reported
Posadzki P. & Ernst E. (2011)	16 RCTs (NA)	Pts with musculoskeletal pain	OMT (including SMT in some of the trials)	Tiredness, "mild adverse effects"<br><br>No SAEs	"In four trials only adverse effects were reported. Given the fact that 12 trials did not report adverse reactions at all safety of OMT remains unclear."	Not reported
Rubinstein S. M. et. al (2011)	26 RCTs (2435 pts in total)	Pts with chronic low-back pain	SMT (including a few studies on mobilization)	Muscle soreness, stiffness, transient increase in pain, aggravated conditions, tiredness, increased pain<br><br>No SAEs	"Slightly more than one-third of the studies reported on adverse events. Adverse events in the SMT group were limited to muscle soreness, stiffness, and/or transient increase in pain. None of the studies registered any serious complications in either the experimental or control group."	Not reported
Walker B. F.	10 RCTs	Pts with nonspecific low-	SMT	"Minor, transient, exacerbations of	"Adverse effects were reported in only two of the included studies [one of these trials included SMT]. From these two	Not reported

Author (year) ref	Included studies on SMT (pts. in total receiving SMT)	Population receiving SMT	Interventions including SMT	AEs reported* associated with SMT)	Conclusion on AEs from SMT (quote)	Quality of the evidence for AEs (tool)
et. al (2011)	(NA)	back pain		symptoms"<br><br>No SAEs	studies, 16 out of a total of 106 participants who received the chiropractic interventions reported minor, transient, exacerbations of symptoms. None of the included studies reported any serious adverse effects in participants that received the chiropractic interventions. However, relatively small and short-term RCTs included in this review are not the best study design for detecting adverse events, and longer term large observational studies are needed to provide a valid evaluation of adverse effects, particularly those that are uncommon or rare."	
Carlesso L. C. et. al (2010)	3 CSs, 14 RCTs (NA)	Pts with cervical pain or cervicogenic headache	Cervical SMT (including three studies on cervical mobilization)	Transient neurological symptoms, increased neck pain, headache, fatigue<br><br>No SAEs	"Seventeen of 76 identified citations resulted in no major AE. Two pooled estimates for minor AE found transient neurological symptoms [RR 1.96 (95% CI: 1.09-3.54) p<0.05]; and increased neck pain [RR 1.23 (95% CI: 0.85-1.77) p>.05] [both estimates are based on two trials, that used only SMT of the neck as intervention, n=285 and n=389 respectively]. Forty-four studies (58%) were excluded for not reporting AE. No definitive conclusions can be made due to a small number of studies, weak association, moderate study quality, and notable ascertainment bias."	Major/ catasthropic adverse events: - ; Minor adverse events - transient neurological symptoms: low; Minor adverse events - increased neck pain: low. (GRADE)
Carnes D. et. al (2010)	8 pCohorts, 31 RCTs (25179 pts in total)	Pts in studies reporting AEs following manual therapy	Manual therapy (including SMT in the majority of the trials)	Headaches *(very sparse information with respect to AEs)*<br><br>**SAEs**: Serious neurological complaints', 'unbearably severe side effects', 'significant adverse events', 'alarming' adverse events *(very sparse information with respect to SAEs)*	"Nearly half of patients after manual therapy experience adverse events that are short-lived and minor; most will occur within 24 h and resolve within 72 h. The risk of major adverse events is very low, lower than that from taking medication. We suggest that risk is inherent in all health interventions and should be weighed against patient-perceived benefit and alternative available treatments. (...) Of the eight studies [prospective cohorts], one (Thiel et al., 2007) reported 14 cases of `unbearably severe side effects' in 4712 treatments (0.13%). Thiel et al. (2007) reported an upper risk rate for `serious adverse events' using Hanley's `rule of three' (Hanley and Lippman-Hand, 1983) of approximately 0.01% (3/28,109 consultations). Combining all the data from the cohort studies (Table 1) we estimated, an upper 95% CI incidence risk rate of major adverse events (as per our definition) of 0.007% (0/42,451) after treatment or 0.01% (0/22,833) per patient. (...) There were no reports of any major adverse events in any trial [RCTs]. The 31 RCTs included 2281 participants who received manual therapy and 2779 who received other therapies. Fifteen trials reported that no adverse events occurred regardless of the intervention administered. We estimated an upper incidence rate of major adverse events of ~0.13% (0/2301) after manual therapy treatment."	Not reported
Ernst E. (2010)	23 CRs (26 pts in total)	Pts who died following treatments from a chiropractor	Treatments from a chiropractor (including SMT)	NA<br><br>**SAEs**: Death (including vascular accident leading to thrombosis and cerebral infarction)	"In conclusion, numerous deaths have been associated with chiropractic neck manipulations. There are reasons to suspect that under-reporting is substantial and reliable incidence figures do not exist. The risks of chiropractic neck manipulations by far outweigh their benefits. (...) The type of complication associated with death frequently related to a vascular accident leading to thrombosis and cerebral	Not reported

Author (year) ref	Included studies on SMT (pts. in total receiving SMT)	Population receiving SMT	Interventions including SMT	AEs reported* associated with SMT)	Conclusion on AEs from SMT (quote)	Quality of the evidence for AEs (tool)
					infarction."	
Hahne A. J. et. al (2010)	3 RCTs (NA)	Pts with lumbar disc herniation with associated radiculopathy	Manipulation (may be entirely SMT)	None	"Three trials [including one trial on SMT] reported at least 1 adverse event in conservative treatment groups. (...) In 1 trial [on SMT compared with mechanical traction], 2 of the 50 participants receiving mechanical traction fainted. (...) A further 4 trials reported that there were no adverse events associated with conservative treatment [including one trial on SMT]. (...) Six trials made no mention of adverse events [including one trial on SMT]. (...) no adverse events related to manipulation were reported by the trials in our review."	Not reported
Kaminskyj A. et. al (2010)	8 RCTs (NA)	Pts with asthma	SMT (including a few studies on chiropractic care or chiropractic manipulation, not further specified)	Exacerbations of asthma<br><br>No SAEs	"None of the studies indicated any adverse effects or evidence of harm (other than exacerbations of asthma) to patients treated by chiropractors. Studies by Balon and Nielsen were the only ones to mention adverse effects/reactions as part of the article and to formally state that there were no adverse events. All other articles included in this study did not mention adverse effects. None of the included articles included a comprehensive list of possible adverse effects from the intervention."	Not reported
Shin B.-C. et. al (2010)	12 CRs (18 pts in total)	Pts experiencing AEs following SMT	SMT	Herniated discs<br><br>**SAEs**: Cauda equina syndrome, dural tear, vertebral fracture, vertebral subluxation, stroke	"In conclusion, adverse effects after spinal manipulation have been reported in the Korean literature with some regularity. Their true incidence, however, remains unknown. (…) In cases in which the lumbar region had been manipulated, the adverse effects usually pertained to herniated discs or cauda equina syndrome. In cases in which the cervical region had been manipulated, the most serious complications were dural tear, vertebral fracture, vertebral subluxation and stroke. In the majority of cases, the onset of symptoms was soon after treatment. Most patients made full recoveries but, in several instances, lasting neurological deficits remained."	Not reported
Boudreau R. et. al (2009)	1 RCT, 3 SRs, 1 HTA (>52 pts in total)	Pts with acute or chronic lower back pain	SMT	Headache, tiredness (*most common; very sparse information with respect to AEs*)<br><br>**SAEs**: Not specified	"Evidence on adverse events was minimal, but the literature consistently reported that patients commonly experienced mild adverse events, and rarely experienced serious adverse events."	Not reported
Boudreau R. & Spry C. (2009)	1 CR (1 pts in total)	Pts with syringomyelia	SMT	None	"No adverse effects were observed."	Not reported
Brurberg K. G. et. al (2009)	1 CS, 1 RCT (>695 pts in total)	Infants suspected with kinematic imbalance due to suboccipital strain	SMT and osteopathy	Mild bradycardia<br><br>No SAEs	"In a large patient series it was reported about mild bradycardia following manual therapeutic KISS-treatment. The effect on heart rate was short-lived (3 to 25 seconds), and can hardly be defined as pathological."	Not reported
Gouveia L. O. et. al (2009)	100 CRs, 2 CCs, 3 rCohorts, 6 pCohorts, 12 surveys, 1 RCT (>2838 pts in total)	Pts in studies reporting AEs associated with chiropractic interventions	Chiropractic interventions (almost entirely SMT or cervical SMT)	RCT: Increased neck pain or stiffness, headache. CC: NA. pCohorts: local discomfort, exacerbation of pain, and radiation and headaches. (occurred most commonly in the first 24 hours after manipulation, were transient, mild, and	"Adverse reactions are frequent after spinal manipulation ranging from 33% to 60.9%, mostly increased pain or stiffness. However, the frequency of serious adverse events is not established varying between 5 strokes/100,000 manipulations to 1.46 serious adverse events/10,000,000 manipulations and 2.68 deaths/10,000,000 manipulations, with stroke being the most frequent. (...) There is no robust data concerning the incidence or prevalence of adverse reactions after chiropractic. Further investigations are urgently needed to	Not reported

Author (year) [ref]	Included studies on SMT (pts. in total receiving SMT)	Population receiving SMT	Interventions including SMT	AEs reported* associated with SMT)	Conclusion on AEs from SMT (quote)	Quality of the evidence for AEs (tool)
				benign). rCohorts: myelopathies, radiculopathies, vertigo, diminishment or loss of consciousness, radiculopathy, sudden onset of new and unusual headache and neck pain. CRs: herniated disc, radiculopathy, myelopathy.<br><br>**SAEs**: RCT: None CC: vertebrobasilar accidents, cervical artery dissection. pCohorts: None. rCohorts: strokes, transitory ischemic accidents, acute subdural hematoma, death, spinal cord injury (including myelopathy, tetraparesis, central cord syndrome, or paraparesis), cauda equina syndrome, Brown-Séquard syndrome, vertebral artery occlusion, strokes. CRs: strokes, spinal fluid leak presented as intracranial hypotension, spinal epidural hematoma, cauda equina syndrome, diaphragmatic palsy, pathologic fractures of vertebra.	assess definite conclusions regarding this issue."	
Hunt K. J. et. al (2009)	1 RCT (NA)	Pts with carpal tunnel syndrome	Chiropractic care (including SMT)	Sore neck<br><br>No SAEs	"In the intervention group, adverse effects were noted for one patient who complained of a 'temporary sore neck at the end of the treatment'. It is unclear from the report whether this was resolved at the end of the study."	Not reported
Khorsan B. et. al (2009)	6 CRs, 6 CSs, 2 CCs, 9 surveys, 1 RCT, 2 SRs, 4 narrative reviews, 1 cohort study, 1 'small non-randomized static-group comparison study (preexperimental design)' (>297 pts in total)	Pregnant women with back pain and other related symptoms	SMT (including some mobilization and OMT)	NA<br><br>**SAEs**: Cervical spine fracture (pt. had an underlying undetected spinal tumor, fracture resolved without residual effects)	"Case reports and narrative reviews were included in Table 4 to describe the nature and severity of reported adverse events related to SMT or OMT during pregnancy. The majority of studies, including case reports, did not include reporting of adverse effects in their manuscript. Two narrative reviews discussed possible contraindications to SMT during pregnancy and 3 clinical studies formally reported that no adverse events occurred. (...) high quality clinical trials on safety and effectiveness should be a priority."	Not reported
Reiman M. P. et. al	2 CSs, 3 pCohorts, 1 RCT, 1 descriptive	Pts with lumbar spinal stenosis	SMT (including one study on 'manual physical	Not specified<br><br>**SAEs**: Not specified/may be	"Initial group characteristics (C) and adverse effects (K) were also either not often described or difficult to ascertain."<br>*(From table: one study including SMT describes adverse*	Not reported

Author (year) [ref]	Included studies on SMT (pts. in total receiving SMT)	Population receiving SMT	Interventions including SMT	AEs reported* associated with SMT)	Conclusion on AEs from SMT (quote)	Quality of the evidence for AEs (tool)
(2009)	study, 1 prognostic cohort (>76 pts in total)		therapy to the thoracic and lumbar spine')	none	*events. No further details provided (e.g. which kinds of AEs or if any AEs were observed))*	
Miley M. L. et. al (2008)	4 CRs, 3 CCs, 1 survey, 1 SR, 8 prospective and retrospective case series studies, 5 reviews, 4 opinion and expert commentary pieces (NA)	Pts in studies reporting vertebral artery dissection and ischemic stroke following cervical SMT	Cervical SMT	NA<br><br>**SAEs**: Vertebral artery dissection and ischemic stroke	"The evidence that both supports and negates a causal association between VAD [vertebral arterial dissection] and CMT [cervical manipulative therapy] has been thoroughly reviewed and appraised. The evaluated evidence includes case-control studies, prospective and retrospective case series, case reports, surveys, and expert commentaries, which comprises a weak to moderately strong platform from which to draw our conclusions. In summary, we have found the burden of evidence to support a cause-and-effect relationship between CMT with VAD and subsequent stroke. Although we confidently make this assertion based on the evidence presented, we agree that a comprehensive prospective study must be conducted in a collective effort between all CMT practitioners to further examine this causal relationship, the incidence of VAD and stroke caused by CMT and the therapeutic efficacy of CMT. (...) Published estimates of the incidence of VAD and stroke after CMT range from 1 in 5.8 million to 1 in 500018,20. The best available estimate is from the case-control study by Rothwell et al, which concludes that for every 100,000 persons 45 years of age who receive CMT, approximately 1.3 cases of vertebral artery dissection or occlusion attributable to CMT would be observed within 1 week of manipulative therapy."	"Weak to moderately strong evidence exists to support causation between CMT and VAD and associated stroke." (Sir Bradford Hill's criteria for causation and the strength of the research designs)
Stuber K. J. & Smith D. L. (2008)	2 CSs, 1 rCS, 1 survey, 1 single-group pretest-posttest (285 pts in total)	Women with pregnancy-related low-back pain	Manipulation	None	"None of the studies indicated any adverse effects or evidence of harm to either the pregnant woman or unborn child from the treatments rendered. However, only the study by Lisi formally reported that there were no adverse events; the remaining studies did not comment one way or the other."	Not reported
Vernon H. & Humphreys B. K. (2008)	6 RCTs (178 pts in total)	Pts with chronic mechanical neck pain	SMT	"New discomfort" in neck, superficial phlebitis, more pain, mild exacerbation of pain<br><br>No SAEs	"Mild, temporary pain-related adverse effects were reported in 6–17% of subjects in three studies. No major adverse reactions (defined as any reaction requiring additional medical intervention at any time) were reported in any of these studies."	Not reported
Chou R. & Huffman L. H. (2007)	16 SRs, 2 trials (NA)	Pts with low-back pain	SMT	Worsening lumbar disc herniation *(very sparse information with respect to AEs)*<br><br>**SAEs**: Cauda equina syndrome *(very sparse information with respect to SAEs)*	"Five systematic reviews consistently found that serious adverse events after spinal manipulation (such as worsening lumbar disc herniation or the cauda equina syndrome) were very rare. One systematic review found no serious complications reported in more than 70 controlled clinical trials. Including data from observational studies, the risk for a serious adverse event was estimated as less than 1 per 1 million patient visits."	"good" (based on the type, number, size and validity of studies, strength of association, consistency of results within and between study designs, directness of evidence)
Ernst E.	28 CRs, 5 rCSs, 2 pCSs, 3 CCs, 3	Pts in studies reporting AEs	SMT	CRs: Oedema, nerve injury, disc herniation,	"Spinal manipulation, particularly when performed on the upper spine, is frequently associated with mild to moderate	Not reported

Author (year) ref	Included studies on SMT (pts. in total receiving SMT)	Population receiving SMT	Interventions including SMT	AEs reported* associated with SMT)	Conclusion on AEs from SMT (quote)	Quality of the evidence for AEs (tool)
(2007)	surveys, 1 SR, (>924 pts in total)	following SMT		haematoma, bone fracture. rCSs: Vertigo, disc prolapse, bone fractures, worsening of symptoms, radiculopathy, spinal cord injuries (myelopathy). pCSss: headache, stiffness, local discomfort, radiating discomfort, fatigue, radiating pain, tiredness. CC: NA. Surveys: Radiculopathies. SR: NA.<br><br>**SAEs**: CRs: Dissection of the vertebral arteries, dural tear. rCSs: Stroke, vertebral artery dissection, cerebrovascular accidents, worsening of symptoms, spinal cord injuries (quadriparesis, central cord syndrome or paraparesis), cauda equina syndrome. pCSs: None CCs: Carotid artery dissection, vertebral artery dissection, vertebrobasilar accidents, vascular accidents. Surveys: Cerebrovascular accidents. SR: Cervical artery dissection.	adverse effects. It can also result in serious complications such as vertebral artery dissection followed by stroke. Currently, the incidence of such events is not known. (...) The case reports confirm previous reports associating upper spinal manipulation with a range of complications. The most serious problems, which some experts now describe as 'well-recognized', are vertebral artery dissections due to intimal tearing as a result of overstretching the artery during rotational manipulation. This seems to occur most commonly at the level of the atlantoaxial joint. Intimal injury can be followed by intramural bleeding or pseudoaneurysm formation, which can result in thrombosis, embolism or arterial spasm. The retrospective case series confirm that spinal manipulation is associated with risks such as vascular accidents and non-vascular complications. (...) Most of the incidents reported in case series or surveys had not been previously reported, indicating that under-reporting may frequently be high. The two prospective case series corroborate the results from several earlier investigations showing that mild to moderate adverse effects occur in a large proportion of patients receiving spinal manipulation. These adverse effects are transient and non-serious but nevertheless seriously affect many patients. (...) Case-control and other studies confirm that upper spinal manipulation is associated with risks and that spinal manipulation is an independent risk factor for vertebral artery dissection. (...) The three surveys disclose more complications. They suggest that many therapists are now becoming aware of the risks of spinal manipulation. Two of the surveys also confirm that under-reporting is frequently close to 100%. (...) Dissection of the vertebral arteries was the most common problem [in the CRs]; other complications included dural tear, oedema, nerve injury, disc herniation, haematoma and bone fracture."	
Gross A. R. et. al (2007)	4 RCTs (NA)	Pts with mechanical neck disorders, neck disorders with headache or neck disorder with radicular findings	Manipulation	NA<br><br>No SAEs	"We found that minor, transient, and reversible side effects consisting of increased symptoms were occasionally reported. A valid estimate of clinically significant, uncommon, and rare adverse events cannot be made from these trials. Adverse effects of longterm steroid therapy and manipulation have been well described."<br>*(Does not specify in which studies the AEs were observed, or if these studies included SMT)*	Not reported
Hawk C. et. al (2007)	93 CRs, 29 CSs, 14 RCTs, 9 SRs, 1 cohort, 33 "other" (pilot studies, quasi-experimental (nonrandomized) designs, single-group interventions and other small experimental or pre-experimental	Pts with non-musculoskeletal conditions	Chiropractic care (including SMT in majority of the studies)	Lumbar soreness, muscle soreness, irritability, headache, neck pain, low-back pain, joint and muscle soreness<br><br>No SAEs	"The adverse effects reported for SMT for all age groups and conditions were rare and, when they did occur, transient and not severe."	Not reported

Author (year) ref	Included studies on SMT (pts. in total receiving SMT)	Population receiving SMT	Interventions including SMT	AEs reported* associated with SMT)	Conclusion on AEs from SMT (quote)	Quality of the evidence for AEs (tool)
	designs)<br>(NA)					
Luijsterburg P. A. J. et. al (2007)	2 RCTs<br>(175 pts in total)	Pts with lumbosacral radicular syndrome	Manipulation (not further specified)	None	None<br>*(One trial reported no AEs and the other trial did not report AEs)*	Not reported
Vernon H. & Humphreys B. K. (2007)	14 RCTs<br>(701 pts in total)	Pts with cervical pain	SMT (including a few studies on manipulation, not further specified)	Increased neck pain or headache, "minor side effects"<br><br>No SAEs	"There were no adverse reactions to any of the therapies [for acute neck pain] reported in any of these studies. This could be interpreted to mean that no adverse reactions actually occurred or that they were not monitored and, therefore, not reported (…) There were no major adverse events reported in any of these trials [for chronic neck pain]."	Not reported
Vernon H. et. al (2007)	9 RCTs<br>(593 pts in total)	Pts with chronic mechanical neck pain	SMT	"Minor side effects"<br><br>No SAEs	"In none of these trials were any major adverse reactions reported."<br>*(From table, reported for one of the included studies: "No major side effects in either group. For minor side effects in the first 4 wk: Manip: 16% Mob = 8.7% P = .051".)*	Not reported
Gemmell H. & Miller P. (2006)	4 RCTs, 1 randomized trial with a 2x2x2 factorial design<br>(>79 pts in total)	Pts with non-specific cervical pain	SMT	Not specified/may be none<br><br>**SAEs**: Not specified/may be none	"Only one paper reported on adverse effects from manual therapy."<br>*(No further details provided (e.g. which kinds of AEs or if any AEs were observed))*	Not reported
Proctor M. et. al (2006)	3 RCTs<br>(>162 pts in total)	Women with dysmenorrhea	SMT	Not specified<br><br>**SAEs**: Not specified/may be none	"Only one trial (n = 138) reported the number of adverse effects experienced. Results showed no significant differences in the adverse effects experienced by participants in the HVLA and sham treatment groups after one cycle of treatment (Peto OR 1.51, 95% CI 0.25 to 8.95)."	Not reported
Snelling N. J. (2006)	1 survey, 4 RCTs, 1 SR, 3 reports, 1 retrospective study<br>(>214 pts in total)	Pts with disc herniation	SMT	Additional disc herniation, radioculopathy<br>*(very sparse information with respect to AEs)*<br><br>**SAEs**: Cauda equina syndrome, spinal cord injury<br>*(very sparse information with respect to SAEs)*	"A review on safety of spinal manipulation in the treatment of disc herniation has recently been published, therefore this will be dealt with in less depth. (...) Evidence for harms is based primarily on case reports, and incidences would appear to be rare, though underreporting may be a significant problem. No data was available from the insurance companies on incidences of adverse events. (...) The most recent comprehensive review specific to this question, which draws together much of the published literature, estimates that the risk of causing further disc herniation or cauda equina syndrome by spinal manipulation in patients presenting with a herniated lumbar disc to be one in 3.7 million. (...) With respect to harms, none of the included trials suggested greater complications in the manipulation groups, however when an adverse event occurs rarely, data from trials are not very useful, as they would need to involve huge numbers of patients in order to demonstrate any increase in adverse events."	Not reported
Brown A. et. al (2005)	2 RCTs, 14 SRs, 2 non-randomized controlled trials<br>(NA)	Pts with low-back pain	SMT (including some studies on chiropractic care or manipulation, not further specified)	Not specified<br><br>**SAEs**: Cauda equina syndrome *(very sparse information with respect to SAEs)*	"The results of the review suggest that serious adverse events are unlikely to occur with chiropractic treatment for LBP. (…) Another systematic review noted that the development of cauda equina syndrome can be a serious complication of lumbar spinal manipulation, yet the incidence was low."	Not reported

Author (year) ref	Included studies on SMT (pts. in total receiving SMT)	Population receiving SMT	Interventions including SMT	AEs reported* associated with SMT)	Conclusion on AEs from SMT (quote)	Quality of the evidence for AEs (tool)
Ernst E. (2005)	14 CRs<br>(14 pts in total)	Pts with ophthalmological AEs following SMT	SMT of the upper spine	Ptosis, the ophthalmological consequences (nystagmus, Wallenberg's syndrome, loss of vision, hemianopsia, ophthalmoplegia, diplopia, Horner's syndrome, ptosis).<br><br>**SAEs**: Vertebral artery dissection, basilar artery infarction, stroke, dissection of carotid artery, cerebellar infarction, epidural haematoma	"Upper spinal manipulation is associated with ophthalmological adverse effects of unknown frequency. Ophthalmologists should be aware of its risks. Rigorous investigations must be conducted to establish reliable incidence figures. (…) The ophthalmological consequences included nystagmus, Wallenberg's syndrome, loss of vision, hemianopsia, ophthalmoplegia, diplopia, Horner's syndrome and ptosis. In many cases, visual deficits were the first signs. The onset of symptoms was frequently instant. In several instances, the eventual clinical outcome entailed a permanent deficit. In the majority of cases, the causality between USM and the ophthalmological adverse effect was certain or likely."	Not reported
Hondras M. A. et. al (2005)	2 RCTs<br>(NA)	Pts with asthma	SMT	None	"One of the included studies (Nielsen 1995) reported data on adverse events. (…) Adverse events: stated that no side-effects were reported by patients as a result of the manipulation."	Not reported
Lisi A. J. et. al (2005)	7 CRs, 5 CSs, 1 RCT, 2 cohort studies, 1 controlled clinical trials<br>(183 pts in total)	Pts with symptomatic lumbar disk diseases	SMT	Worsening of pain<br>*(very sparse information with respect to AEs)*<br><br>No SAEs	"Consistent descriptions of adverse effects among the included studies were lacking. This is summarized in Table 6. Consequently, no conclusions regarding safety could be made. (...) Moreover, several studies commonly cited as describing significant adverse effects after lumbar HVLASM in cases of disk pathology did not meet our inclusion criteria."<br>*(From table 6: 2 trials "stated that no adverse effects occurred", 4 trials "clearly described any worsening of pain during treatment period", 3 trials "clearly described no worsening of pain during treatment period")*	Not reported
Rubinstein S. M. et. al (2005)	2 CCs<br>(7 pts in total)	Pts in studies reporting cervical artery dissection following cervical SMT	Cervical SMT	NA<br><br>**SAEs**: Cervical artery dissection	"A strong association was found for manipulative therapy (ORadj , 3.8; 95% CI, 1.3 to 11). However, although an important confounder (ie, neck pain before the onset of stroke) was adjusted for in regression analysis, selection and information bias were most probably present. The study by Rothwell et al lacked control for confounding and included cases of occlusive stroke along with unconfirmed dissections. The number of cases identified in both studies were few (n=7) and in only 57% (n=4/7) of the cases was there a clear temporal association between the treatment and the onset of dissection (using 24 hours after the treatment as the cutoff point)."	Not reported
Brønfort G. et. al (2004)	2 RCTs<br>(85 pts in total)	Pts with tension-type headache	SMT	Neck soreness and stiffness<br><br>No SAEs	"The results of the trials included in this review [on non-invasive physical treatments, including SMT] do not suggest that any of these therapies are associated with important risks of severe adverse reactions. Side effects have been addressed mostly for spinal manipulation."	Not reported
Ernst E. (2004)	33 CRs, 14 retrospective investigations<br>(340 pts in total)	Pts experiencing cerebrovascular complications associated with spinal manipulation	SMT	Headache, confusion, stupor vertigo, visual disturbances, left-sided tinnitus, vertigo, nausea, vomiting, diplopia, throbbing headache, instant pain followed by headache nausea vomiting double vision and dural	"The most frequently reported complication was stroke due to arterial dissection after cervical spinal manipulation. Considering the popularity of spinal manipulative therapies, the overall incidence of such complications is probably low; however, no reliable figures can be generated through this or any other data available to date. It is concluded that serious cerebrovascular complications of spinal manipulation continue to be reported. Their incidence is unknown. Large and rigorous prospective studies are necessary in order to define	Not reported

Author (year) ref	Included studies on SMT (pts. in total receiving SMT)	Population receiving SMT	Interventions including SMT	AEs reported* associated with SMT)	Conclusion on AEs from SMT (quote)	Quality of the evidence for AEs (tool)
				tear<br><br>**SAEs**: Dissection of arteries (carotid and vertebral and intracranial) basilar artery infarction, Wallenberg's syndrome, transitory neurological deficits, emboli partial and complete loss of vision, ischaemic lesion in medulla oblongata, 'signs suggesting brain stem dysfunction', loss of consciousness, epidural haematoma, acute infarctions, stroke, cauda equina syndrome, radiculopathy, subarachnoid haemorrhage, dural tear, , Horner's syndrome, subdural haematoma, incomplete cervical cord injury, paralysis, disc herniation, 'locked-in syndrome', myelopathy, radiculopathy, loss of hearing, epileptic fit, rip fracture, paresthesiae, paraplegia, bone fractures, 'nerve damage', disk prolapse, paraparesis, spinal cord compression, vertebral artery occlusion, death	the risks of spinal manipulation accurately."	
Lenssinck M.-L. B. et. al (2004)	5 RCTs (NA)	Pts with tension-type headache	Manipulation (SMT or not specified)	Neck soreness and stiffness<br><br>No SAEs	"Only two studies reported side effects [one of these did not include SMT]. The study of Boline et al. (1995) provided information on the side effects of chiropractic spinal manipulation and amitriptyline. In proximally 4% of the patients receiving spinal manipulation side effects like short-term neck soreness and stiffness were reported after the first treatment."	Not reported
Oduneye F. (2004)	2 RCTs (128 pts in total)	Pts witch chronic cervical pain	SMT	Increased neck pain or headache, severe thoracic pain, increased radicular pain<br><br>No SAEs	"We found inadequate evidence to assess reliably, any adverse effects of spinal manipulation in people with chronic neck pain. (...) The first study did not report on the adverse effects of treatment. The second study reported that no permanent injuries occurred in any treatment group, and there was no significant difference between groups in the incidence of adverse effects at 12 month follow up (P=0.49). Increased neck pain or headache was experienced in 12% of participants (6/64 with spinal manipulation alone v 8/64 with spinal manipulation plus exercise v 9/63 with machine-assisted exercise). One participant receiving spinal manipulation alone experienced severe thoracic pain, and one	Not reported

Author (year) ref	Included studies on SMT (pts. in total receiving SMT)	Population receiving SMT	Interventions including SMT	AEs reported* associated with SMT)	Conclusion on AEs from SMT (quote)	Quality of the evidence for AEs (tool)
					participant receiving spinal manipulation plus exercise experienced increased radicular pain. Both of these adverse effects were self-limiting."	
Oliphant D. (2004)	2 surveys, 8 review articles, 9 prospective/retrospective studies (NA)	Pts in studies reporting AEs from lumbar SMT	Lumbar SMT	"Mild aggravation of symptoms", radiculopathy, disk prolapse, or not specified *(very sparse information with respect to AEs)*<br><br>**SAEs**: Worsening of lumbar disc herniation, cauda equina syndrome, or not specified	"The apparent safety of spinal manipulation, especially when compared with other accepted treatments for LDH, should stimulate its increased use in the conservative treatment plan of LDH. (...) Spinal manipulation for the treatment of LDH appears to be very safe, and there is no sound basis to recommend against a trial of spinal manipulation of patients with LDH, although limited lumbar flexion and gentle technique are suggested to further reduce the risk. (...) Disk herniation is the number one claim against chiropractors; yet, it appears likely that lumbar disk prolapse could occur only in an already fissured and fragmented disk. Even in patients presenting with LDH, the risk of spinal manipulation appears minimal, especially compared with other common treatments for LDH, such as NSAIDs and surgery, and spinal manipulation may be no more dangerous than activities of daily living, such as a cough or stumble. More research is needed to determine accurately the incidence of disk injury/increased disk symptoms following spinal manipulation; under what conditions, if any, spinal manipulation can actually cause a disk herniation; the benefit of spinal manipulation in the treatment of LDH compared with natural history, other conservative treatments, and surgery; and which patients will benefit most from which type of treatment. (...) In Koes et al review of trials of effectiveness of manipulation for acute and chronic low back pain, few papers specifically mentioned the absence of adverse effects, but most did not mention adverse effects at all. This may be because none occurred during these trials involving over 1500 patients or simply they were not recorded as part of the data. However, if any significant complications had been known to occur, they would probably have been mentioned, at least as a reason for dropout. (...) The numbers that these calculations have been based on can be argued to be rough estimates at best, and therefore with each calculation, the accuracy of this risk estimate may have been reduced. However, there has been an increased emphasis on evidence-based care. This risk was calculated according to the best evidence available, and the numbers used err in favor of overestimating the risk."	Not reported
Ernst E. (2003)	2 CRs, 1 SR (2 pts in total)	Children and adolescents experiencing SAEs following SMT	SMT	Holocord astrocytoma, respiratory distress, holocord astrocytoma with excessive acute necrosis *(very sparse information with respect to AEs)*<br><br>**SAEs**: Cerebrovascular accident, quadriplegia and seizures, vertebral arteries dissected causing ischaemia of the caudal brain stem with subarachnoid	"At present, it is impossible to provide reliable incidence figure [for the risk of unconventional therapies]. (...) Chiropractic upper spinal manipulation (e.g. high-velocity thrusts) has been repeatedly associated with serious adverse events, e.g. cerebrovascular accidents. A recent systematic review summarised 177 published cases of injury. The age range of the patients thus affected was 4 months to 87 years. American paediatricians described the case of an infant with congenital torticollis treated with chiropractic spinal manipulation. Within a few hours of this therapy the child began suffering from respiratory distress, quadriplegia and seizures. A holocord astrocytoma with excessive acute necrosis, believed to be caused by the neck manipulation, was found and resected. A 3-month-old girl was seen by a German	Not reported

Author (year) ref	Included studies on SMT (pts. in total receiving SMT)	Population receiving SMT	Interventions including SMT	AEs reported* associated with SMT)	Conclusion on AEs from SMT (quote)	Quality of the evidence for AEs (tool)
				haemorrhage, death	physiotherapist who treated her with forced rotation and retraction of the head. As a result, both vertebral arteries dissected causing ischaemia of the caudal brain stem with subarachnoid haemorrhage. The diagnosis was confirmed with MRI and the child died."	
Ernst E. J. (2002)	4 CRs, 3 SRs (>4 pts in total)	Elderly in studies reporting AEs following SMT	Cervical SMT	Bone fracture, pain and swelling in temporomandibular joint for one month, myelopathy, paresthesias in all extremities *(very sparse information with respect to AEs)*<br><br>**SAEs**: Multiple spinal compression fractures, thoracic epidural hematoma, bilateral vertebral artery dissections, brainstem stroke *(very sparse information with respect to SAEs)*	"A recent review of the published literature (1925-1997) located 177 case reports of serious complications after manipulations of the cervical spine. The age range of the patients thus affected was 4 months to 87 years. Osteoporosis should be regarded as a contra-indication to spinal manipulation. In addition to these probably rare events, spinal manipulation is associated with frequent (~50%) transient mild adverse effects."	Not reported
Ernst E. (2002)	31 CRs (42 pts in total)	Pts experiencing SAEs following cervical SMT	Cervical SMT	Paraesthesiae, pain and reduced mobility of right arm, diaphragmatic palsy, intimal tear of right vertebral artery, retinal artery occlusion, disc herniation, cervical myelopathy, spinal stenoses, spinal epidural haematoma, dissection of carotid artery, profuse vomiting, vertigo and Horner's syndrome, Brown–Séquard syndrome, radiculopathy of right arm, Dural tear, lesions of the cervical nerve root, cervical myelopathy, subdural haematoma<br><br>**SAEs**: Arterial dissection (usually of the vertebral arteries, causing stroke), serious sequelae (such as permanent visual field loss), permanent neurological deficit, dissection of right intracranial artery, cerebral infarct, cerebellar infarction, emboli, death	"In conclusion, serious complications of cervical spine manipulation appear to occur regularly. Their incidence is essentially unknown and should be established as a matter of urgency through adequately designed investigations. (…) Arterial dissection, usually of the vertebral arteries, causing stroke was the most common serious adverse event (at least 18 cases). In most instances, the acute onset of symptoms after the manipulation made a causal relationship likely. Symptoms often developed quickly — after or during therapy — and varied widely according to the exact nature of the injury. The eventual outcome was often not reported, but included serious sequelae, such as permanent visual field loss, permanent neurological deficit and death (serious sequelae in at least 17 cases)."	Not reported

Author (year) ref	Included studies on SMT (pts. in total receiving SMT)	Population receiving SMT	Interventions including SMT	AEs reported* associated with SMT)	Conclusion on AEs from SMT (quote)	Quality of the evidence for AEs (tool)
Gerritsen A. A. M. et. al (2002)	1 RCT (45 pts in total)	Pts with carpal tunnel syndrome	SMT	"Minor side effects"<br><br>No SAEs	"Minor side effects (e. g. nausea, abdominal discomfort, headache) were reported for diuretics, NSAIDs, oral steroids and chiropractic treatment."<br>*(Not specified which of the side effects are from the trial including chiropractic treatment (SMT))*	Not reported
Gross A. R. et. al (2002)	6 surveys, 7 RCTs, 6 SRs (NA)	Pts with mechanical neck disorders, neck disorders with headache of cervical origin or neck disorders with radicular signs or symptoms	SMT (including a few studies on mobilization)	Minimal benign reaction lasting less than 24 h, some/more/new discomfort, dizziness, visual disturbances and ear symptoms, headache, nausea, myelopathies, radiculopathy, disc prolapse and increased pain, dizziness, nausea, headache, nystagmus, vomiting, brachalgia, brachalgia with neurological deficit, loss of consciousness, acute wry neck, tiredness, hot skin, local discomfort, radiating discomfort<br><br>**SAEs**: Cerebral vascular accident (CVA), neurological complications (moderate to severe nature, and death)	"The true risks are unclear. Available estimates are as follows: the lowest reported estimate for risk of irreversible injury when applying manipulation is one in 20,000 (...) The accuracy of the rate is limited, as a result of the poor quality of the literature on which it is based. However, the weight of the evidence suggests that there is some risk."	"The accuracy of this estimate [estimate from the SRs] is low, as it is based on level V evidence" (NA)
Gross A. R. et. al (2002)	10 RCTs (NA)	Pts with mechanical neck disorders	Manipulation (may be entirely SMT)	Increased neck or headache pain, increased radicular pain, severe thoracic pain, persistent acute pain, "customary reaction of minimal benign reactions"<br><br>No SAEs	"Seven trials reported on adverse events [including two trials not using SMT]. The adverse events reported include more pain, discomfort, dizziness, visual disturbances and ear symptoms. Most studies did not appear to have any systematic method for recording adverse reactions. (...) Adverse events were inconsistently reported in trials. When reported, they were categorized as benign transient side-effects. There was no report of reversible or irreversible serious complications."	Not reported
Stevinson C. & Ernst E. (2002)	1 CR, 1 CS, 1 CC, 3 rCohorts, 5 surveys, 1 SR, 3 reviews, 1 retrospective analysis (>2357 pts in total)	Pts in studies reporting AEs associated with SMT	SMT	Local discomfort, headache, tiredness, radiating discomfort, dizziness, nausea, hot skin, disk herniation, arterial spasm<br>*(the seven first AEs, is the most common, ordered with respect to frequency*)<br><br>**SAEs**: Vertebrobasilar accidents (some causing death), progression of radicular symptoms to cauda equina syndrome, cerebral complications, dislocations and fractures	"In conclusion, serious complications of spinal manipulation seem to be rare, whereas less serious adverse events occur frequently. (…) However, without reliable data about the incidence of specific risks, it is difficult to achieve the correct balance between providing adequate information and causing unnecessary alarm."	Not reported

Author (year) [ref]	Included studies on SMT (pts. in total receiving SMT)	Population receiving SMT	Interventions including SMT	AEs reported* associated with SMT)	Conclusion on AEs from SMT (quote)	Quality of the evidence for AEs (tool)
				(often accompanied by spinal cord compression), progression to cauda equina syndrome (mostly occurring with manipulation to the lumbar region), vertebrobasilar accidents (mostly occurring after rotational cervical manipulation), dissection of the vertebral artery at the atlantoaxial joint, with intimal tear, intramural bleeding, or pseudoaneurysm leading to thrombosis or embolism. Arterial dissection and lesions of the brain stem. Cerebrovascular accidents, often with permanent neurologic deficits, including death		
Bronfort G. et. al (2001)	9 RCTs (400 pts in total)	Pts with chronic headache	SMT	Muscle soreness and neck stiffness (*most common*)<br><br>No SAEs	"In the studies comparing SMT [spinal manipulative therapy] with amitriptyline [two trials], more than half the patients taking amitriptyline reported side effects such as drowsiness, dry mouth, and weight gain, and approximately 10% were withdrawn from the studies due to drug intolerance. In comparison, only 5% of the patients receiving SMT reported side effects, the most frequent being muscle soreness and neck stiffness. These effects are common and considered normal reactions to spinal manipulation. No serious complications (i.e., vertebrobasilar accidents) were reported in any of the studies included in this review. The risk of serious complications from SMT is considered low."	Not reported
Ernst E. (2001)	5 prospective investigations (>2016 pts in total)	Pts experiencing SAEs following SMT	SMT	Transient exacerbation of symptoms, discomfort, reduction in the ability to work, local discomfort, headache, fatigue and discomfort outside the area of treatment, extracranial arterial dissections<br><br>No SAEs	"No reliable data exist about the incidence of serious adverse events. These data indicate that mild and transient adverse events seem to be frequent. Serious adverse events are probably rare but their incidence can only be estimated at present."	Not reported
Ernst E. & Harkness E. (2001)	8 RCTs (NA)	Pts in studies receiving SMT (includes pts with asthma, phobia, chronic LBP etc.)	SMT	Exacerbation of asthma, soreness in low-back region<br><br>No SAEs	"The risks of SM [spinal manipulation] are still under-researched. In the trials reviewed above, adverse effects were not mentioned in the weaker studies [three studies]; Nielsen et al. explicitly stated that no adverse events occurred, Balon et al. only noted exacerbation of asthma symptoms, and Hondras et al. found some minor soreness at the site of SM." *(AEs reported in 2 RCTs, no AEs reported in 2 RCTs, AEs not reported in 4 RCTs)*	Not reported
Ernst E.	7 RCTs	Pts in studies receiving SMT	SMT	Exacerbation of asthma symptoms, minor soreness	"In the trials reviewed above, adverse effects were not mentioned in the weaker studies [three studies], while Nielsen	Not reported

Author (year) ref	Included studies on SMT (pts. in total receiving SMT)	Population receiving SMT	Interventions including SMT	AEs reported* associated with SMT)	Conclusion on AEs from SMT (quote)	Quality of the evidence for AEs (tool)
(2000)	(NA)	(includes pts with chronic low-back pain, children with nocturnal enuresis, chronic asthmatic patients, pts suffering from phobias, dysmenorrhea)		at the site of SMT *(very sparse information with respect to AEs)*<br><br>No SAEs	et al. explicitly stated that no adverse events occurred, Balon et al. only noticed exacerbation of asthma symptoms and Hondras et al. found some minor soreness at the site of SM [spinal manipulation]. (...) Serious complications of SM seem to be very rare. They include vertebral artery dissection (upper spinal manipulation) and canda equina syndrome (lower spinal manipulation). At present the incidence of such serious adverse events can only be estimated."	
Magee D. J. et. al (2000)	1 small uncontrolled trial (10 pts in total)	Pts with soft tissue neck injury following trauma	SMT	None	"(…) no study showed any harmful effects for physical therapy intervention. [includes the one trial on SMT]"	Not reported
Fabio R. P. D. (1999)	116 CRs (177 pts in total)	Pts experiencing 'injuries' following cervical SMT	Cervical SMT (but "The specific type of manipulation was not described in 46% (n=82)")	Wallenberg syndrome, Horner syndrome, joint dislocation, other (including visual deficits, hearing loss, balance deficits, phrenic nerve injury) *(Ordered with respect to frequency)*<br><br>**SAEs**: Arterial dissection or spasm, brain-stem injury, cerebral/cerebellar injury, spinal cord injury, thrombosis, locked-in syndrome, brain death, death *(Ordered with respect to frequency)*	"Although the risk of injury associated with MCS [manipulation of the cervical spine] appears to be small, this type of therapy has the potential to expose patients to vertebral artery damage that can be avoided with the use of mobilization (nonthrust passive movements). The literature does not demonstrate that the benefits of MCS outweigh the risks. (...) Death occurred in 18% of the cases (n=32)."	Not reported
Haldeman S. M. et. al (1999)	115 CRs (115 pts in total)	Pts experiencing vertebrobasilar artery dissection following SMT	SMT	NA<br><br>**SAEs**: Vertebrobasilar artery dissection	"Recent reports of specific ultrastructural aberrations in connective tissue or a unique phenotypically mild Type I collagen tissue disease in patients with spontaneous cervical artery dissection raise the possibility that certain people have either an inherited or acquired disorder of unknown origin that increases the fragility of vertebral arteries to trauma. If this is determined to be true, it may eventually be possible by means of a laboratory test to screen patients who are at risk for vertebrobasilar artery dissection before they engage in vigorous sporting activities or undergo cervical manipulation. Until that happens, vertebrobasilar artery dissection after neck movement, trauma, or manipulation should be considered a rare, random, unpredictable complication associated with these activities."	Not reported
Vernon H. et. al (1999)	6 RCTs (176 pts in total)	Pts with tension-type, and cervicogenic headache	SMT	Neck stiffness<br><br>No SAEs	None *(AEs reported in 1 RCT, AEs not mentioned 5 RCTs)*	Not reported
Aker P. D. et. al (1996)	4 RCTs (NA)	Pts with mechanical neck	Manipulation (not further	Not specified	"Adverse effects have not been well documented. If we exclude data from the three trials in which patients with neck pain were not separated from those with low back pain	Not reported

Author (year) [ref]	Included studies on SMT (pts. in total receiving SMT)	Population receiving SMT	Interventions including SMT	AEs reported* associated with SMT)	Conclusion on AEs from SMT (quote)	Quality of the evidence for AEs (tool)
		pain	specified)	No SAEs	[including one trial on SMT], 1254 patients were randomised in 21 randomised controlled trials. Six trials reported a total of 16 patients with increased symptoms or side effects resulting from treatment. No serious complications or deaths were reported."<br>*(No further details provided, e.g. which trials reported AEs, and if these included trials on SMT. Also the type of AEs was not stated.)*	
Assendelft W. J. J. et. al (1996)	295 CRs, 3 surveys, 1 SR (>1795 pts in total)	Pts in studies reporting 'complications' following SMT	SMT	<u>CRs</u>: Disc herniation, other types of complications (dislocations, factures, spinal cord compression and neglegence or nondetection of preexisting serious conditions; 56 cases). The results include residual handicap (86 cases), complete recovery (44 cases)<br><br>**SAEs**: <u>CRs:</u> Vertebrobasilar accidents (165 cases), progression of radicular symptoms to cauda equina syndrome (61 cases), cerebral complications other than vertebrobasilar accidents (13 cases). The results include death (29 cases).	"It is difficult to estimate the incidence of SMT complications as they are probably underreported in the literature. Most non-VBA complications can be prevented by excluding patients with contraindications for SMT. (...) Refferral for SMT should not be made to practitioners applying rotatory cervical manipulation. (...) While complications of spinal manipulation have not yet been studied in prospective surveys, the incidence of serious complications is generally considered to be low. (...) Vertebrobasilar accidents occur mainly after a cervical manipulation with a rotatory component."	Not reported
Hurwitz E. L. et. al (1996)	43 CRs, 14 CSs, 10 RCTs, 1 cohort study, "and 145 articles on complications" (>935 pts in total)	Pts with cervical pain and headache	Cervical SMT	Vertebrobasilar accidents (VBA) (with consequences such as Wallenberg's syndrome), spinal cord compression, vertebrobasilar artery spasm or stenosis, other permanent functional impairment<br><br>**SAEs**: Vertebrobasilar accidents (VBA) (with consequences such as brain stem and/or cerebellar infarction, locked-in syndrome), vertebral fracture, tracheal rupture, diaphragm paralysis, internal carotid hematoma, cardiac arrest, brain stem and/or cerebellar infarctions, vertebrobasilar artery dissection, death, serious neurologic deficit, paralysis	"Articles documenting more than 110 cases of complications allegedly arising from cervical spine manipulation have been published in English. (...) Complications occurred in patients who had received manipulation uneventfully in the past, without obvious risk factors for cerebral vascular accidents (e.g., arteriosclerosis, hypertension, heavy smoker, oral contraceptive use), without previous trauma, and with negative results on positional tests designed to assess vertebral artery sufficiency.(...) Rotational manipulations were used in 45 of 55 (82%) of the cases for which the type of treatment was described. (...) In summary, of the 118 documented cases of VBA and other complications described above, 21 patients died and 52 survived with serious neurologic deficit, paralysis, or other permanent functional impairment. (...) No complications were reported among the subjects who received cervical spine manipulation in the studies reviewed for efficacy, a total of 892 patients. (...) Systematic reports of complication rates are necessary for calculation of a more precise estimate of risk. The true incidence of serious complications from cervical spine manipulation may be smaller or higher and is probably modified by clinical presentation, appropriate history taking and examination procedures, and the use of specific types of manual therapy."	Not reported

Author (year) ref	Included studies on SMT (pts. in total receiving SMT)	Population receiving SMT	Interventions including SMT	AEs reported* associated with SMT)	Conclusion on AEs from SMT (quote)	Quality of the evidence for AEs (tool)
Dabbs V. & Lauretti W. J. (1995)	5 surveys, 2 report, 1 review, and data from insurance company (NA)	Pts treated for cervical pain in studies providing an estimate for the risk of SAEs or death, or pts with cervical pain in studies on cervical SMT	Cervical SMT	NA<br><br>**SAEs**: Stroke, vertebral artery injury, death or not specified<br>*(very sparse information with respect to SAEs)*	"The best evidence indicates that cervical manipulation for neck pain is much safer than the use of NSAIDs, by as much as a factor of several hundred times. There is no evidence that indicates NSAID use is more effective than cervical manipulation for neck pain."	Not reported
Shekelle P. G. et. al (1992)	8 CRs, 25 clinical trials, 1 review, 1 community-based study, 1 personal communication (>1500 pts in total)	Pts with low-back pain	SMT	NA<br>*(very sparse information with respect to AEs)*<br><br>**SAEs**: Paraplegia from meningeal hematoma, cauda equina syndrome, death<br>*(very sparse information with respect to SAEs)*	"No systematic report of the frequency of complications from spinal manipulative therapy has been published. No complications were reported in the clinical trials of manipulation, which in total comprised more than 1500 patients treated with manipulation. All else that is known comes from case reports, and there is concern that these represent only a fraction of the total number of complications. A review of the world's literature by Ladermann showed 135 case reports of serious complications, including 18 deaths, due to manipulation. (...) Cervical manipulation had a greater number of complications, of a more serious nature, than did lumbar manipulation. (...) Estimating the frequency with which the cauda equina syndrome occurs in patients undergoing lumbar spinal manipulation is difficult (...) we estimate the rate of occurrence of the cauda equina syndrome as a complication of lumbar spinal manipulation to be on the order of less than one case per 100 million manipulations. Even if the number of cases of the cauda equina syndrome is underestimated by tenfold, the complication rate is still low. These data suggest that the risk of lumbar spinal manipulation is small and that it may vary by the clinical condition with which the patient presents. No firm conclusions about the precise level of the complication rate may be drawn, however, because there are few available data. Systematic reports of the rate of complications of spinal manipulation are needed to help estimate better the risk of this procedure."	Not reported

*Not an exhaustive listing of all AEs observed in the included studies of the SRs. In the case of an overwhelming amount of different AEs listed by the SRs, only the SRs own summarizations of the AEs are reported in this table together with the note '(*most common*)'. 'No SAEs' includes reporting that no AEs and/or SAEs were present, or only reporting AEs which were not SAEs.

AEs, adverse events; NA, no data available; CC, case-control study; CR, case report; CS, case series; HTA, health technology assessment; HVLA; high-velocity low amplitude; OMT, osteopathic manipulative treatment/therapy; pCohort, prospective cohort; pCS, prospective case series; pts, patients; rCohort, retrospective cohort; rCS, retrospective case series; RCT, randomised controlled trial; S, survey; SAEs, serious adverse events; SMT, spinal manipulative therapy; SR, systematic review.
