# Supplementary material for: The risk associated with spinal manipulation: an overview of reviews
Source: Syst Rev. 2017 Mar 24;6:64. doi: 10.1186/s13643-017-0458-y (PMC5366149; doi:10.1186/s13643-017-0458-y)
Supplement: Supplementary file 7 — Table showing which studies the estimates for the incidence of SAEs are based on. (DOCX 25 kb) [file 13643_2017_458_MOESM7_ESM.docx]

**Study Matrix**

Matrix showing the studies (left column) that the estimates (top row) are based on

| **Author (year)** | Todd A. J. et al. (2014) | Carnes D. et al. (2010) | Gouveia L. O. et al. (2009) | Miley M. L. et al. (2008) | Chou R. & Huffman L. H. (2007) | Snelling N. J. (2006) | Oliphant D. (2004) | Gross A. R. et al. (2002) | Stevinson C. & Ernst E. (2002) | Assendelft W. J. J. et al. (1996) | Hurwitz E. L. et al. (1996) | Dabbs V. & Lauretti W. J. (1995) | Shekelle P. G. et al. (1992) |
| --- | --- | --- | --- | --- | --- | --- | --- | --- | --- | --- | --- | --- | --- |
| SRs |  |  |  |  |  |  |  |  |  |  |  |  |  |
| Oliphant (2004)* |  |  |  |  | x | x |  |  |  |  |  |  |  |
| Stevinson & Ernst (2002)* |  |  |  |  | x |  |  |  |  |  |  |  |  |
| Assendelft et al. (1996)* |  |  |  |  |  |  | x | x |  |  |  |  |  |
| Hurwitz et al. (1996)* |  |  |  |  |  |  |  | x |  |  |  |  |  |
| Dabbs & Lauretti (1995)* |  |  |  |  |  |  |  | x | x |  |  |  |  |
| Shekelle et al. (1992)* |  |  |  |  |  |  | x |  |  | x |  |  |  |
| Reviews |  |  |  |  |  |  |  |  |  |  |  |  |  |
| Chestnut (2004) |  |  |  | x |  |  |  |  |  |  |  |  |  |
| Haldeman et al. (2002) |  |  |  | x |  |  |  |  |  |  |  |  |  |
| Pistolese (1998) | x |  |  |  |  |  |  |  |  |  |  |  |  |
| Haldeman et al. (1993) |  |  |  |  |  |  |  |  |  |  |  | x |  |
| Powell et al. (1993) |  |  |  |  |  |  | x |  | x |  |  |  |  |
| Haldeman & Rubinstein (1992) |  |  |  |  |  |  | x |  |  |  |  |  | x |
| Patjin (1991) |  |  |  |  |  |  | x |  |  |  | x | x |  |
| Wolff (1978) |  |  |  |  |  |  |  |  | x |  |  |  |  |
| pCohorts |  |  |  |  |  |  |  |  |  |  |  |  |  |
| Garner et al. (2007) |  | x |  |  |  |  |  |  |  |  |  |  |  |
| Rubinstein et al. (2007) |  | x |  |  |  |  |  |  |  |  |  |  |  |
| Thiel et al. (2007) |  | x |  |  |  |  |  |  |  |  |  |  |  |
| Cagnie et al. (2004) |  | x |  |  |  |  |  |  |  |  |  |  |  |
| Barrett and Breen (2000) |  | x |  |  |  |  |  |  |  |  |  |  |  |
| Leboeuf-Yde et al. (1997) |  | x |  |  |  |  |  |  |  |  |  |  |  |
| Senstad et al. (1996a) |  | x |  |  |  |  |  |  |  |  |  |  |  |
| Senstad et al. (1996b) |  | x |  |  |  |  |  |  |  |  |  |  |  |
| Surveys |  |  |  |  |  |  |  |  |  |  |  |  |  |
| Rivett & Milburn (1997) |  |  |  |  |  |  |  | x |  |  |  |  |  |
| Coulter et al. (1996) |  |  | x |  |  |  |  |  |  |  |  |  |  |
| Klougart et al. (1996) |  |  | x |  |  |  |  |  | x |  |  |  |  |
| Lee et al. (1995) |  |  | x |  |  |  |  |  |  |  |  | x |  |
| Haynes (1994) |  |  | x |  |  |  |  |  |  | x |  |  |  |
| Carey (1993) |  |  | x |  |  |  |  |  |  |  | x |  |  |
| Michaeli (1993) |  |  | x |  |  |  | x |  |  | x |  |  |  |
| Henderson & Cassidy (1988) |  |  |  |  |  |  |  |  |  |  |  | x |  |
| Dvorak (1985) |  |  | x |  |  |  |  |  | x | x | x | x |  |
| Gutmann (1983) |  |  | x |  |  |  |  |  |  |  | x | x |  |
| Reports |  |  |  |  |  |  |  |  |  |  |  |  |  |
| Eder & Tilscher (1990) |  |  |  |  |  |  |  |  |  |  |  | x |  |
| Jaskoviak (1980) |  |  |  |  |  |  |  |  |  |  |  | x |  |
| CCs |  |  |  |  |  |  |  |  |  |  |  |  |  |
| Rothwell et al. (2001) |  |  |  | x |  |  |  |  | x |  |  |  |  |
| Retrospective studies |  |  |  |  |  |  |  |  |  |  |  |  |  |
| Stern et al. (1995) |  |  |  |  |  |  | x |  |  |  |  |  |  |
| Community-based study |  |  |  |  |  |  |  |  |  |  |  |  |  |
| Shekelle et al. (1991) |  |  |  |  |  |  |  |  |  |  | x |  | x |
| Prospective studies |  |  |  |  |  |  |  |  |  |  |  |  |  |
| Nyiendo & Haldeman (1987) |  |  |  |  |  |  | x |  |  |  |  |  |  |
| Senstad et al. (1997) |  |  |  |  |  |  | x |  |  |  |  |  |  |
| Kirkaldy-Willis & Cassidy (1985) |  |  |  |  |  |  | x |  |  |  |  |  |  |
| Hadler et al. (1987) |  |  |  |  |  |  | x |  |  |  |  |  |  |
| BenEliyahu (1996) |  |  |  |  |  |  | x |  |  |  |  |  |  |
| Barrett & Breen (2000) |  |  |  |  |  |  | x |  |  |  |  |  |  |
| Discussion papers |  |  |  |  |  |  |  |  |  |  |  |  |  |
| Doyle (2011) | x |  |  |  |  |  |  |  |  |  |  |  |  |
| Personal communication |  |  |  |  |  |  |  |  |  |  |  |  |  |
| Haldeman and Rubinstein |  |  |  |  |  |  |  |  |  |  |  |  | x |
| Letters |  |  |  |  |  |  |  |  |  |  |  |  |  |
| Hosek et al. (1981) |  |  |  |  |  |  |  |  | x |  |  |  |  |
| RCTs |  |  |  |  |  |  |  |  |  |  |  |  |  |
| Any RCTs (number) |  | x (31) |  |  |  |  | x (2) |  |  |  |  |  |  |

* These systematic reviews are already included in this overview.

CC, case-control study; pCohort, prospective cohort; RCT, randomised controlled trial; SR, systematic review.
