# Supplementary material for: The risk associated with spinal manipulation: an overview of reviews
Source: Syst Rev. 2017 Mar 24;6:64. doi: 10.1186/s13643-017-0458-y (PMC5366149; doi:10.1186/s13643-017-0458-y)
Supplement: Supplementary file 8 — Table showing the calculations of RRs of having the opinion that spinal manipulative therapy is safe or harmful, respectively, if a ‘yes’ was obtained in the individual AMSTAR items, for the 33 reviews, whose objective was to investigate adverse events. (PDF 186 kb) [file 13643_2017_458_MOESM8_ESM.pdf]

Opinions Regarding the Safety of Spinal Manipulation Therapy

The risk ratio of having the opinion that spinal manipulation therapy is safe or harmful, respectively, if a 'yes' was obtained in the individual AMSTAR items (33 reviews, whose objective was to investigate adverse events).

	**Risk ratio (RR)**			
	RR (95% CI) for communicating that SMT is safe	P values	RR (95% CI) for communicating that SMT is harmful	P values
AMSTAR #1	Not estimable†	-	Not estimable†	-
AMSTAR #2	Not estimable†	-	0.7 (0.2 to 2.4)	0.516
AMSTAR #3	1.1 (0.1 to 8.2)	0.943	1.5 (0.4 to 5.2)	0.516
AMSTAR #4	Not estimable†	-	1.8 (0.7 to 4.6)	0.318
AMSTAR #5	Not estimable†	-	0.8 (0.2 to 4.4)	0.824
AMSTAR #6	1.1 (0.1 to 8.2)	0.943	3.2 (0.5 to 20.8)	0.131
AMSTAR #7	4.0 (0.8 to 20.1)	0.079	Not estimable†	-
AMSTAR #8	Not estimable†	-	Not estimable†	-
AMSTAR #9	2.5 (0.4 to 15.8)	0.364	0.8 (0.2 to 4.4)	0.824
AMSTAR #10	Not estimable*	-	Not estimable*	-
AMSTAR #11	Not estimable*	-	Not estimable*	-

AMSTAR, A Measurement Tool to Assess Systematic Reviews; CI, confidence interval; RR, risk ratio; SMT, spinal manipulation therapy.
*No SRs had a "yes" for this item.
†No SRs had a "yes" for this item and communicated "safe".
(For descriptions of each AMSTAR item, see foot note for Table 2)
